# Metformin rescues migratory deficits of cells derived from patients with periventricular heterotopia

**DOI:** 10.15252/emmm.202216908

**Published:** 2023-08-23

**Authors:** Cedric Bressan, Marta Snapyan, Marina Snapyan, Johannes Klaus, Francesco di Matteo, Stephen P Robertson, Barbara Treutlein, Martin Parent, Silvia Cappello, Armen Saghatelyan

**Affiliations:** ^1^ CERVO Brain Research Center Quebec City QC Canada; ^2^ Université Laval Quebec City QC Canada; ^3^ University of Ottawa Ottawa ON Canada; ^4^ Max Planck Institute of Psychiatry Munich Germany; ^5^ Biomedical Center (BMC) Ludwig Maximilian University of Munich Munich Germany; ^6^ Dunedin School of Medicine University of Otago Dunedin New Zealand; ^7^ Max Planck Institute of Molecular Cell Biology and Genetics Dresden Germany

**Keywords:** autophagy, DCHS1, FAT4, human neural progenitor cells, periventricular neuronal heterotopia, Autophagy & Cell Death, Genetics, Gene Therapy & Genetic Disease, Neuroscience

## Abstract

Periventricular neuronal heterotopia (PH) is one of the most common forms of cortical malformation in the human cortex. We show that human neuronal progenitor cells (hNPCs) derived from PH patients with a DCHS1 or FAT4 mutation as well as isogenic lines had altered migratory dynamics when grafted in the mouse brain. The affected migration was linked to altered autophagy as observed *in vivo* with an electron microscopic analysis of grafted hNPCs, a Western blot analysis of cortical organoids, and time‐lapse imaging of hNPCs in the presence of bafilomycin A1. We further show that deficits in autophagy resulted in the accumulation of paxillin, a focal adhesion protein involved in cell migration. Strikingly, a single‐cell RNA‐seq analysis of hNPCs revealed similar expression levels of autophagy‐related genes. Bolstering AMPK‐dependent autophagy by metformin, an FDA‐approved drug, promoted migration of PH patients‐derived hNPCs. Our data indicate that transcription‐independent homeostatic modifications in autophagy contributed to the defective migratory behavior of hNPCs *in vivo* and suggest that modulating autophagy in hNPCs might rescue neuronal migration deficits in some forms of PH.

## Graphical abstract

Time‐lapse imaging of hNPCs derived from patients with periventricular neuronal heterotopia grafted in the mouse brain revealed abnormal migratory dynamics due to the longer duration of the stationary phases. These changes were linked to altered autophagic flux leading to the accumulation of focal adhesion molecules. Bolstering autophagy by metformin, an FDA‐approved drug, rescued neuronal migration.

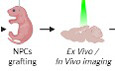

**The paper explained**

**Problem**

Periventricular neuronal heterotopia (PH) is characterized by the abnormal migration and retention of neuronal cells close to the ventricular area. PH can be caused by mutations in the cadherin ligand–receptor pair *DCHS1* and *FAT4* genes. The dynamics of cell migration *in vivo*, the underlying mechanisms, as well as whether migratory deficits may be rescued by modulating other migration‐promoting pathways remain unknown.

**Results**

Xenografting human neuronal progenitor cells (hNPC) in the neurogenic brain regions of immunodeficient mice revealed that hNPC with a *FAT4* or *DCHS1* mutation or isogenic lines with *FAT4* or *DCHS1* knock‐down displayed abnormal migratory dynamics *in vivo* because of the shorter duration of the migratory phases. Western blot analyses and single‐cell mRNA sequencing of cortical organoids as well as immunohistochemistry and electron microscopic analyses of grafted hNPCs *in vivo* revealed a homeostatic dysregulation of autophagy leading to the accumulation of focal adhesion molecules. Metformin, an FDA‐approved drug, was used to trigger autophagy. It increased autophagic flux and restored the migration of hNPCs derived from PH patients.

**Impact**

Our results indicate that the modulation of autophagy can rescue the migratory deficits observed in neuronal cells derived from PH patients. As metformin is already used for the treatment of diabetes and in pediatric clinical trials, our results open up the possibility of pharmacological interventions in PH patients to counteract, at least partially, the migratory deficits of neuronal progenitors causing a critical malformation. The time windows and associated clinical improvements remain to be evaluated.

## Introduction

Periventricular heterotopia (PH) is a genetically heterogeneous cortical malformation resulting from the retention of early post‐mitotic neuroblasts in the ventricular area (Buchsbaum & Cappello, 2019). Mutations in the protocadherin ligand–receptor pair *DCHS1* and *FAT4* have been shown to underlie certain forms of PH in humans (Cappello *et al*, 2013). DCHS1 and FAT4 are involved in the organization of the apical membrane of neuronal progenitors (Ishiuchi *et al*, 2009). Reducing DCHS1 and FAT4 expression in the developing mouse cortex increases progenitor proliferation, with a concomitant effect on cell differentiation (Cappello *et al*, 2013). *In vitro* studies using human neuronal progenitor cells (hNPCs) and cortical organoids derived from induced pluripotent cells (iPSCs) of patients with a *DCHS1* or *FAT4* mutation have further shown that neuronal migration is altered in a subset of cells underlying this form of PH (Klaus *et al*, 2019). *In vitro* models, however, do not fully recapitulate the complexity of brain neural networks and the influence of different cellular elements and molecular factors on cell migration. Furthermore, the underlying mechanisms of altered neuronal migration of hNPCs with a *DCHS1* or *FAT4* mutation need to be explored. Here, we xenotransplanted hNPCs into the mouse postnatal neurogenic niche and assessed neuronal migration by time‐lapse imaging in acute brain slices. We show that hNPCs derived from patients with a *DCHS1* or *FAT4* mutation, as well as isogenic lines with a *DCHS1* or *FAT4* knock‐down (KD), had altered migratory properties due to the longer duration of the stationary phases. As autophagy is an important homeostatic process that allows migrating cells to cope with migration‐promoting and migration‐inhibiting cues (Bressan *et al*, 2020; Bressan & Saghatelyan, 2020), and as FAT4 and DCHS1 are linked to dysregulated autophagy in other tissues (Wei *et al*, 2019; Sun & Zhang, 2020; Yang *et al*, 2022), we assessed the role of autophagy in the altered migratory phenotypes of cells derived from patients with PH. We show that hNPCs with a *DCHS1* or *FAT4* mutation displayed a transcription‐independent impairment of autophagy that led to abnormal recycling and accumulation of the focal adhesion molecule paxillin. We further show that increasing autophagy by metformin rescued the migratory deficits of cells derived from PH patients.

## Results

**Figure 1:**
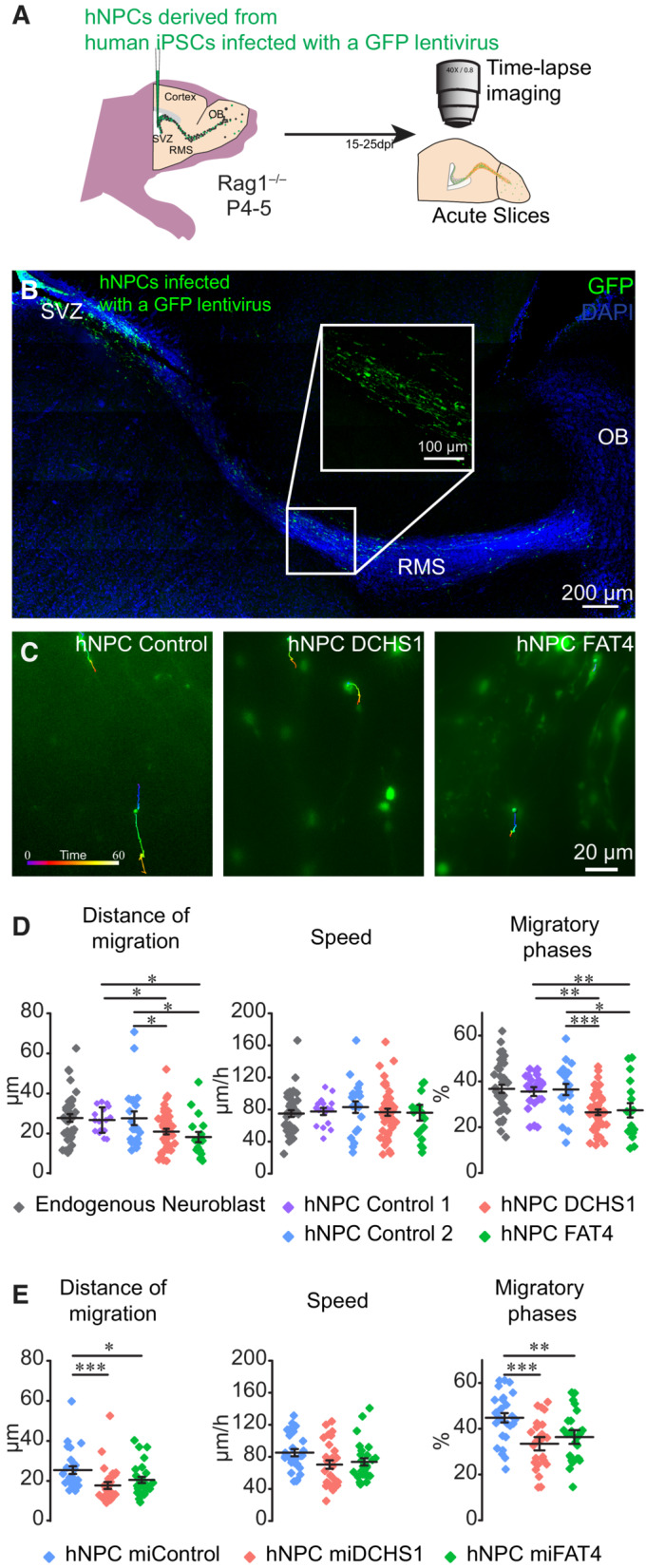
Xenotransplantation of hNPCs into the mouse brain revealed abnormal migratory dynamics of cells with a *DCHS1* or *FAT4* mutation Experimental design for grafting hNPCs derived from healthy individuals, from subjects with a *DCHS1* or *FAT4* mutation and from isogenic lines with *DCHS1* or *FAT4* knock‐down that were infected with a GFP‐expressing lentivirus.Sagittal section of a mouse forebrain showing the distribution of GFP^+^ control hNPCs grafted into the SVZ of Rag1^−/−^ mice at 20 days post‐injection.Example of time‐lapse imaging of control hNPCs and of hNPCs with a *DCHS1* or *FAT4* mutation in the RMS following xenotransplantation into the SVZ.Quantification of the distance of migration (27.8 ± 1.8 μm for endogenous neuroblasts, 25.8 ± 1.6 μm for control hNPCs line 1, 27.6 ± 3.4 μm for control hNPCs line 2 vs. 20.4 ± 1.4 μm or 18.2 ± 2.6 μm for hNPCs with a *DCHS1* or *FAT4* mutation, respectively), speed of migration (73.7 ± 3.4 μm/h for endogenous neuroblasts, 75.4 ± 4.5 μm/h for control hNPCs line 1, 83 ± 7.3 μm/h for control hNPCs line 2 vs. 76.9 ± 4.5 μm/h or 75.9 ± 9.8 μm/h for hNPCs with a *DCHS1* or *FAT4* mutation, respectively), and percentage of migratory phases (37.6 ± 1.8% for endogenous neuroblasts, 35.6 ± 1.9% for control hNPCs line 1, 36.5 ± 2.4% for control hNPCs line 2 vs. 28.7 ± 1.3% or 27.4 ± 3.1% for hNPCs with a *DCHS1* or *FAT4* mutation, respectively) of hNPCs derived from control subjects and from patients with a *DCHS1* or *FAT4* mutation grafted in Rag1^−/−^ mice. *n* = 46 cells from two mice for endogenous neuroblasts, *n* = 16 and 21 cells from three and 12 mice for control hNPCs line 1 and line 2, respectively, *n* = 47 cells from seven mice for hNPCs with a *DCHS1* mutation, and *n* = 17 cells from seven mice hNPCs with a *FAT4* mutation. *P < 0.05, **P < 0.005, ***P < 0.001 with a one‐way ANOVA followed by a *post hoc* Fisher's LSD test. Individual values and mean ± SEM for all the time‐lapse imaging experiments are shown.Quantification of migratory dynamics in isogenic lines with a *DCHS1* or *FAT4* KD. The distance of migration (25.4 ± 1.9 μm for control hNPCs vs. 16.8 ± 1.7 μm or 19.6 ± 1.5 μm for hNPCs with a *DCHS1* or *FAT4* KD, respectively), speed of migration (83.0 ± 4.4 μm/h for control hNPCs vs. 68.2 ± 5.3 μm/h or 71.5 ± 4.6 μm/h for hNPCs with a *DCHS1* or *FAT4* KD, respectively), and percentage of migratory phases (44.8 ± 2.1% for control hNPCs vs. 33.0 ± 1.9% or 35.9 ± 2.0% for hNPCs with a *DCHS1* or *FAT4* KD, respectively) of isogenic hNPCs with a *DCHS1* or *FAT4* KD grafted in Rag1^−/−^ mice. *n* = 27 cells from five mice for control hNPCs, *n* = 28 cells from five mice for hNPCs with a *DCHS1* KD, and *n* = 27 cells from four mice for hNPCs with a *FAT4* KD. *P < 0.05, **P < 0.005, ***P < 0.001 with a one‐way ANOVA followed by a *post hoc* Fisher's LSD test. Individual values and mean ± SEM for all the time‐lapse imaging experiments are shown. Source data are available online for this figure.

To assess the migration of hNPCs derived from patients with PH *in vivo*, we used previously characterized iPSCs derived from fibroblasts collected from two control individuals (Klaus *et al*, 2019) and from two patients with PH who carry compound heterozygous mutation in *FAT4* and a homozygous mutation in *DCHS1*, respectively (Cappello *et al*, 2013; Klaus *et al*, 2019). hNPCs derived from these iPSCs were grafted into the subventricular zone (SVZ) of immunodeficient Rag1^−/−^ mice at postnatal days 4–5 (P4‐5; Fig 1A). Grafting hNPCs infected with a GFP‐expressing lentivirus into the SVZ neurogenic niche allowed us to follow the migratory behavior of cells in an *in vivo* context. Xenotransplanted hNPCs derived from two different control individuals migrated along the rostral migratory stream (RMS) together with endogenous mouse neuroblasts and displayed similar migratory dynamics (Fig 1B–D). No differences in the overall distance or speed of migration or the time spent in the migratory phases were observed between control hNPCs and mouse endogenous neuroblasts (Fig 1C and D). Interestingly, however, the migration of hNPCs with a *DCHS1* or *FAT4* mutation was markedly affected (Fig 1C and D, Movie EV1), exhibiting a shortened migration distance due to the shorter time they spent in the migratory phases (Fig 1D). No changes were observed in the speed of migration, which was calculated only during the migratory phases (Fig 1D). To provide further support for these data, we used isogenic lines with a *DCHS1* or *FAT4* knock‐down (KD). We used another control line that had been previously characterized (Deneault *et al*, 2019; Zaslavsky *et al*, 2019) and xenotransplanted hNPCs that were nucleofected with control miRNA or with miRNAs against either *DCHS1* or *FAT4* (Fig 1E). The migratory dynamics of control hNPCs (Fig 1E) were undistinguishable from those observed from two other control lines (Fig 1D). However, hNPCs with a *DCHS1* or *FAT4* KD migrated significantly shorter distances because of decrease in the percentage of the migratory phases, without any noticeable change in the speed of migration (Fig 1E). Altogether, these results are in line with previous observations in cerebral organoids, showing that the resting periods in a subset of cells with a *DCHS1* or *FAT4* mutation are longer during cell migration (Klaus *et al*, 2019) and suggest that abnormal migratory dynamics of cells derived from PH patients can also be observed *in vivo*.

**Figure 2:**
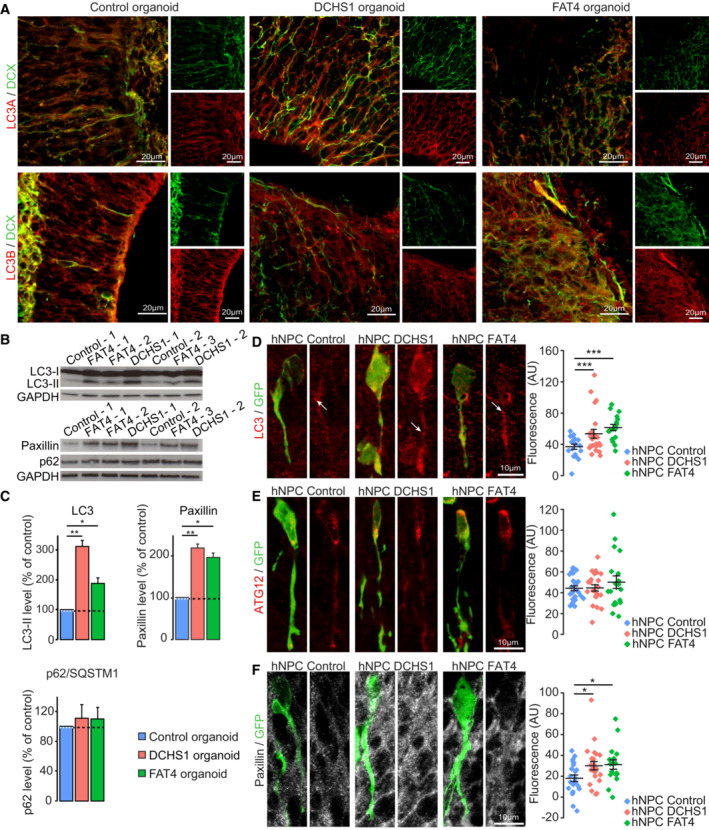
Expression of autophagy‐related proteins and paxillin in organoids and *in vivo* A
Immunostaining either against MAP1LC3A or MAP1LC3B (red) and DCX (green) in organoids derived from the hNPCs of control individuals and from subjects with a *FAT4* or *DCHS1* mutation.B
Western blot for the lipidated form of MAP1LC3 (LC3‐II), p62/SQSTM1, and paxillin in human organoids derived from the hNPCs of control individuals and from subjects with a *FAT4* or *DCHS1* mutation. GAPDH was used as a loading control.C
Quantification of LC3‐II, p62/SQSTM1, and paxillin. *P < 0.05, **P < 0.005, ***P < 0.001 with a one‐way ANOVA followed by a *post hoc* Fisher's LSD test. *n* = 2–3 biological replicates. The data are presented as mean ± SD.D–F
Immunohistochemistry and quantification of LC3 (D), Atg12 (E), and paxillin (F) in hNPCs derived from an unaffected subject and from patients with a *DCHS1* or *FAT4* mutation. The hNPCs were xenotransplanted into the brains of immunodeficient mice 15 days before analysis. Note the increased levels of LC3 and paxillin but not Atg12 in hNPCs derived from patients with a *DCHS1* or *FAT4* mutation. The arrows indicate the punctuate labeling of LC3. Atg12 quantification: *n* = 26, 23, and 19 cells from three mice per condition for control hNPCs and for hNPCs with a *DCHS1* or *FAT4* mutation, respectively. LC3 quantifications: *n* = 18 from three mice for control hNPCs, and *n* = 23 and 17 cells from three and two mice, respectively, for hNPCs with a *DCHS1* or *FAT4* mutation, respectively. Paxillin quantifications: *n* = 23 from three mice for control hNPCs, and *n* = 23 and 17 cells from three and two mice, respectively, for hNPCs with a *DCHS1* or *FAT4* mutation, respectively. *P < 0.05, **P < 0.005, ***P < 0.001 with a one‐way ANOVA followed by a *post hoc* Fisher's LSD test. The data are presented as mean ± SEM. Source data are available online for this figure.

We previously showed that macroautophagy (hereafter autophagy) is homeostatically adapted in response to different migration‐promoting and migration‐inhibiting cues in order to cope with the migratory behavior of neuroblasts by regulating the pace and periodicity of the migratory and stationary phases (Bressan *et al*, 2020). Autophagy is a self‐catabolic pathway that sequesters intracellular materials inside double‐membrane vesicles (autophagosomes) that are then degraded following the fusion of the autophagosomes with lysosomes (Klionsky *et al*, 2016). It has been also reported that FAT4 and DCHS1 may regulate autophagy in cancer cell lines and in renal tubular epithelial cells (Wei *et al*, 2019; Sun & Zhang, 2020; Yang *et al*, 2022). We thus used organoids derived from control cells and from cells with a *DCHS1* or *FAT4* mutation to study the expression and level of microtubule‐associated protein light chain 3 (MAP1LC3), a key protein involved in autophagic vesicle formation (Klionsky *et al*, 2016). MAP1LC3 is present in a non‐lipidated cytoplasmic LC3‐I form and in a lipidated autophagosome membrane‐associated LC3‐II form (Klionsky *et al*, 2016). Immunostaining for MAP1LC3A and MAP1LC3B in organoids revealed that they were expressed in migrating doublecortin (DCX^+^) cells (Fig 2A). Interestingly, a Western blot analysis for MAP1LC3B in organoids revealed a 2‐ to 3‐fold increase in the lipidated LC3‐II form in organoids derived from cells with a *DCHS1* or *FAT4* mutation (Fig 2B and C). As an increase in LC3‐II levels could reflect either an increase in autophagic flux or an autophagy deficiency because of impairment of autophagosome fusion with lysosomes leading to the accumulation of autophagosomes (Klionsky *et al*, 2016), we investigated proteins that may be sequestered and recycled by the autophagic process, such as p62/SQSTM1, a major autophagy substrate (Pankiv *et al*, 2007), or paxillin, a focal adhesion protein involved in neuronal cell migration (Kenific *et al*, 2016; Sharifi *et al*, 2016). Both p62/SQSTM1 and paxillin are direct targets of LC3‐II, and previous work has shown that paxillin turnover by autophagy is independent of p62/SQSTM1 (Kenific *et al*, 2016; Sharifi *et al*, 2016). Interestingly, while the level of p62/SQSTM1 was unaffected (Fig 2B and C), cerebral organoids with a *DCHS1* or *FAT4* mutation exhibited a marked increase in paxillin levels (Fig 2B and C). This points to an impaired autophagy process that may underlie abnormal recycling of paxillin, leading to the altered migratory behavior of hNPCs. This is in line with previous studies, showing that paxillin is a direct target of LC3‐II and is recycled by autophagy to promote neuronal migration (Kenific *et al*, 2016; Sharifi *et al*, 2016; Bressan *et al*, 2020).

To provide support for dysregulated autophagy and paxillin levels in hNPCs *in vivo*, we performed immunolabeling for LC3 and paxillin as well as for Atg12, a marker of autophagosome formation, in fixed brain tissues derived from mice that had been previously xenotransplanted with hNPCs derived from unaffected subject and from patients with a *DCHS1* or *FAT4* mutation (Fig 2D–F). We quantified the immunofluorescent intensity in multiple optical sections of 3D reconstructed GFP^+^ hNPCs and observed increased LC3 levels (Fig 2D) in hNPCs with a *DCHS1* or *FAT4* mutation. Interestingly, the level of Atg12 remained unchanged, indicating that the formation of autophagosomes was unaffected (Fig 2E). We next assessed the level of paxillin (Fig 2F). Our results revealed a significant increase in paxillin immunolabeling in hNPCs with a *DCHS1* or *FAT4* mutation (Fig 2F). These data are in line with our Western blot analysis of organoids derived from NPCs with a *DCHS1* or *FAT4* mutation (Fig 2B and C) and indicate marked changes in autophagy.

**Figure 3:**
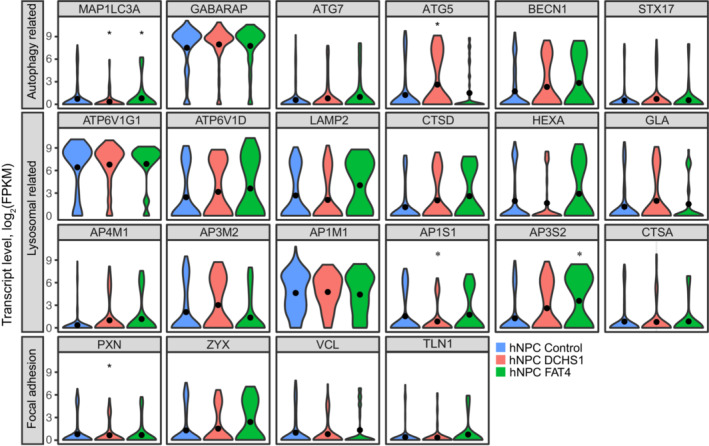
scRNA‐seq analysis of hNPCs in organoids scRNA‐seq analysis of gene expression levels in hNPCs with a *FAT4* or *DCHS1* mutation compared to control cells. Violin plots for the individual genes are shown. The genes are grouped based on their biological function.Source data are available online for this figure.

To determine whether the changes in LC3 levels were due to modifications in the transcription of genes involved in autophagy activation, we analyzed previously published single‐cell RNA‐seq (scRNA‐seq) dataset of control, *DCHS1*, and *FAT4* organoids (Klaus *et al*, 2019). The expression of genes involved in autophagy activation was analyzed in neuronal progenitors identified based on known marker expression (Klaus *et al*, 2019). The analysis revealed no statistical differences in the expression of most genes involved in autophagy (Fig 3). Of the 22 target autophagy genes analyzed, only MAP1LC3A, one of the many ATG8 orthologs identified in humans (Mizushima, 2020), exhibited a very small but statistically significant decrease in expression in hNPCs with a *DCHS1* mutation compared to control hNPCs (Fig 3). Intriguingly, the expression of this gene increased slightly in hNPCs with a *FAT4* mutation (Fig 3). These slight but opposite changes in the expression of the *MAP1LC3A* gene in hNPCs derived from PH patients cannot explain the marked increase in LC3‐II protein levels observed in our Western blot analyses (Fig 2B and C). In addition to *MAP1LC3A*, we also observed a small increase in the level of *ATG5*, but only in hNPCs with a *DCHS1* mutation (Fig 3). As an affected autophagy process may arise from the impaired biogenesis and/or function of lysosomes, we next investigated the expression of lysosomal genes in our scRNA‐seq dataset. Strikingly, as with autophagy‐related genes, no changes in the genes involved in lysosomal biogenesis/function were observed (Fig 3). Of the 16 genes examined, we only observed a slight change in the expression of two genes coding for subunits of the adaptor‐related protein complex that are involved in the vesicular transport of lysosomal enzymes from the Golgi to lysosomes (Robinson & Bonifacino, 2001). *AP1S1* exhibited a small dysregulation in cells with a *DCHS1* mutation but not in cells with a *FAT4* mutation, whereas an upregulation was observed in *AP3S2* in cells with a *FAT4* mutation but not in cells with a *DCHS1* mutation (Fig 3).

As our Western blot analysis shows that the expression and/or recycling of paxillin was altered, we also analyzed the expression levels of focal adhesion molecules in hNPCs with a *FAT4* or *DCHS1* mutation. Of the focal adhesion molecules genes that we analyzed, only the expression level of *PXN* (paxillin) decreased slightly but only in hNPCs with a *DCHS1* mutation (Fig 3), which is inconsistent with the large 2‐fold increase in paxillin levels in organoids from hNPCs with a *DCHS1* or *FAT4* mutation or the immunohistological assessment of paxillin levels *in vivo* in xenotransplanted hNPCs with a *DCHS1* or *FAT4* mutation (Fig 2F). Overall, our results from the scRNA‐seq analysis suggest that the vast majority of genes involved in autophagy activation, lysosomal biogenesis and function, and focal adhesions were largely unaffected despite the very large increases in LC3‐II and paxillin protein levels observed in the Western blot analysis of organoids or *in vivo* in xenotransplanted hNPCs. This points to altered protein homeostasis rather than changes in gene expression.

**Figure 4:**
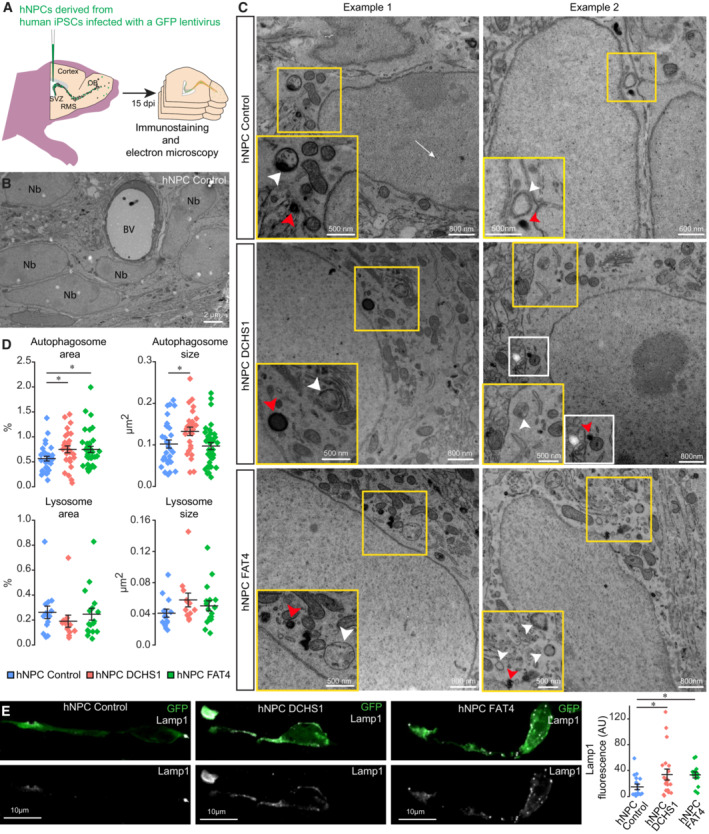
Altered autophagy of grafted hNPCs with a *DCHS1* or *FAT4* mutation revealed by an electron microscopy analysis Experimental design of the EM analysis of grafted hNPCs.Low‐magnification EM images showing immunogold‐labeled hNPCs in Rag1^−/−^ mice. BV indicates a blood vessel. Nb indicates a neuroblast.High‐magnification EM images showing immunogold‐labeled hNPCs in Rag1^−/−^ mice. Autophagosomes are indicated by white arrowheads, while lysosomes are indicated by red arrowheads. White arrows point to GFP immuno‐gold labels.Quantification of the percentage of the total area of autophagosomes (0.57 ± 0.04% for control hNPCs vs. 0.75 ± 0.07% and 0.74 ± 0.06% for hNPCs with a *DCHS1* or *FAT4* mutation, respectively), size (0.10 ± 0.01 μm^2^ for control hNPCs vs. 0.13 ± 0.01 μm^2^ and 0.10 ± 0.01 μm^2^ for hNPCs with a *DCHS1* or *FAT4* mutation, respectively), total area of lysosomes (0.26 ± 0.05% for control hNPCs vs. 0.21 ± 0.05% and 0.25 ± 0.05% for hNPCs with a *DCHS1* or *FAT4* mutation, respectively), and size (0.04 ± 0.01 μm^2^ for control hNPCs vs. 0.06 ± 0.01 μm^2^ and 0.05 ± 0.01 μm^2^ for hNPCs with a *DCHS1* or *FAT4* mutation, respectively). To quantify autophagosomes: *n* = 31 cells from three mice for control hNPCs and *n* = 27 and 35 cells from three and two mice for hNPCs with a *DCHS1* or *FAT4* mutation, respectively. To quantify lysosomes: *n* = 14 cells from three mice for control hNPCs and *n* = 13 and 17 cells from three and two mice for hNPCs with a *DCHS1* or *FAT4* mutation, respectively. *P < 0.05 with a one‐way ANOVA followed by a *post hoc* Fisher's LSD test. Individual values and mean ± SEM for all analyzed cells are shown.Immunohistochemistry and quantification of LAMP1 in hNPCs derived from an unaffected subject and from patients with a *DCHS1* or *FAT4* mutation. hNPCs were xenotransplanted into the brains of immunodeficient mice 15 days before the analysis. *n* = 18 from three mice for control hNPCs, and *n* = 22 and 15 cells from three and two mice, respectively, for hNPCs with a *DCHS1* or *FAT4* mutation, respectively. *P < 0.05 with a one‐way ANOVA followed by a *post hoc* Fisher's LSD test. Individual values and mean ± SEM for all analyzed cells are shown. Source data are available online for this figure.

To determine how autophagy is affected at the ultrastructural level in migrating hNPCs with a *FAT4* or *DCHS1* mutation, we grafted hNPCs into the SVZs of P4‐5 mice and analyzed immunogold‐labeled GFP^+^‐grafted cells in the RMS by electron microscopy (EM). Autophagosomes can be recognized by EM by their double‐membrane vesicles (Klionsky *et al*, 2016). The presence of autophagic vesicles in cells with a *DCHS1* or *FAT4* mutation confirmed that the autophagosome formation machinery was still functional in these cells (Fig 4A–C), which is in line with the Atg12 immunolabeling and scRNA‐seq gene expression data showing largely similar expression levels of autophagy‐related genes (Figs 2 and 3). The size of autophagosomes in cells with a *DCHS1* but not a *FAT4* mutation even increased slightly (Fig 4D). Interestingly, however, the total area occupied by autophagosomes in migrating hNPCs was significantly higher in hNPCs with either a *DCHS1* or *FAT4* mutation than in control hNPCs (Fig 4D). The increase in the area occupied by all the autophagosomes was consistent with our immunofluorescent analysis showing an increase in LC3 labeling and might result from an accumulation of autophagosomes due to defective fusion with lysosomes in hNPCs with a *DCHS1* or *FAT4* mutation.

We next assessed the size and total area occupied by lysosomes in hNPCs derived from an unaffected subject and from patients with a *DCHS1* or *FAT4* mutation. While the total area occupied by lysosomes did not change, a tendency, albeit not significant, toward larger lysosomes in hNPCs with a *DCHS1* or *FAT4* mutation was observed (Fig 4D). It should be noted, however, that fewer lysosomes than autophagosomes were observed in the ultrathin sections of the hNPCs (compare total areas of autophagosomes and lysosomes in Fig 4D), and many hNPCs lacked lysosomes in the cell soma. This may have prevented an accurate estimation of the total area of lysosomes in hNPCs with a *DCHS1* or *FAT4* mutation. We thus performed immunolabeling for LAMP1, a lysosome marker, and quantified the total immunofluorescent intensity in 3D reconstructed GFP^+^ hNPCs *in vivo*. We observed an increase in LAMP1 immunofluorescent intensity in hNPCs with a *DCHS1* or *FAT4* mutation (Fig 4E). These data again point to an affected autophagy process leading to the accumulation of both autophagosomes and lysosomes due to defective fusion.

**Figure 5:**
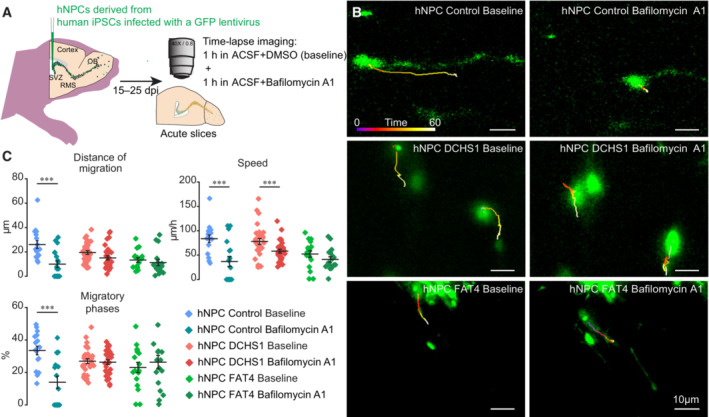
Altered autophagy of grafted hNPCs with a *DCHS1* or *FAT4* mutation revealed by time‐lapse imaging in the presence of bafilomycin A1 Experimental design for time‐lapse imaging of control hNPCs and of hNPCs with a *DCHS1* or *FAT4* mutation under baseline conditions and following an incubation with bafilomycin A1 to inhibit the fusion of autophagosomes with lysosomes.Example of time‐lapse imaging of migrating control hNPCs and of hNPCs with a *DCHS1* or *FAT4* mutation in the RMS following grafting in the SVZ under baseline conditions and in the presence of bafilomycin A1.Quantification of the distance of migration (control hNPCs: 26 ± 3.1 μm vs. 9.9 ± 2.7 μm for baseline and bafilomycin A1, respectively; hNPCs with a *DCHS1* mutation: 19.0 ± 1.5 μm vs. 14.6 ± 1.7 μm for baseline and bafilomycin A1, respectively, and hNPCs with a *FAT4* mutation: 12.8 ± 1.9 μm vs. 10.7 ± 2.4 μm for baseline and bafilomycin A1, respectively); speed of migration (control hNPCs: 83.7 ± 7.6 μm/h vs. 36.9 ± 10.3 μm/h for baseline and bafilomycin A1, respectively; hNPCs with a *DCHS1* mutation: 76.8 ± 6.1 μm/h vs. 56.8 ± 3.9 μm/h for baseline and bafilomycin A1, respectively; and hNPCs with a *FAT4* mutation: 50.9 ± 6.8 μm/h vs. 39.9 ± 5.5 μm/h for baseline and bafilomycin A1, respectively); percentage of migratory phases (control hNPCs: 33.5 ± 2.6% vs. 14.0 ± 3.7% for baseline and bafilomycin A1, respectively; hNPCs with a *DCHS1* mutation: 26.5 ± 1.5% vs. 26.0 ± 1.4% for baseline and bafilomycin A1, respectively; and hNPCs with a *FAT4* mutation: 22.7 ± 3.1% vs. 26.0 ± 4.2% for baseline and bafilomycin A1, respectively). *n* = 17 cells from 11 mice, 30 cells from seven mice, and 17 cells from five mice for control hNPCs and for hNPCs with a *DCHS1* or *FAT4* mutation, respectively. ***P < 0.001 with a Student's *t*‐test. Individual values and mean ± SEM for all the time‐lapse imaging experiments are shown. Source data are available online for this figure.

To test this hypothesis, we used bafilomycin A1, a compound that inhibits the fusion of autophagosome with lysosomes by lowering the pH of lysosomes (Yamamoto *et al*, 1998). We performed time‐lapse imaging of hNPC migration in acute brain slices with and without bafilomycin A1. After 1 h of baseline imaging, migrating NPCs were exposed for 1 h to 4 μM bafilomycin A1 (Fig 5A, Movie EV2). As expected, the control hNPCs exhibited a marked decrease in the distance of migration and the speed and percentage of the migratory phases, with some cells stopping migration completely (Fig 5B and C, Movie EV2). Interestingly, however, the migration distances of hNPCs with either a *DCHS1* or *FAT4* mutation were largely unaffected by bafilomycin A1 (Fig 5B and C) and were comparable to the migratory dynamics of control hNPCs in the presence of bafilomycin A1. These data indicate that the fusion of autophagosomes with lysosomes was already altered in migrating hNPCs derived from patients with PH.

**Figure 6:**
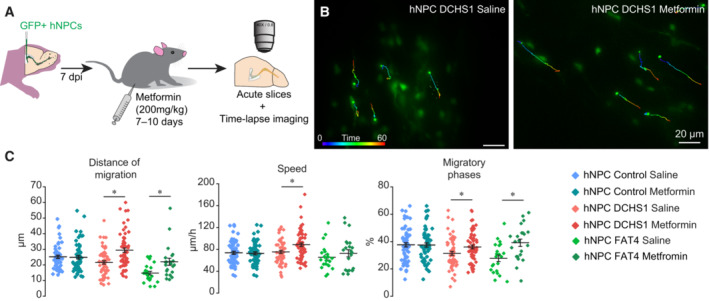
Metformin rescues the migratory deficits of hNPCs with a *DCHS1* or *FAT4* mutation A, B
Experimental design (A) and example (B) of time‐lapse imaging of control hNPCs and of hNPCs with a *DCHS1* or *FAT4* mutation following the i.p. administration of metformin.C
Quantification of the distance of migration (control hNPCs: 25.2 ± 1.1 μm vs. 24.9 ± 1.2 μm, following the saline or metformin treatment, respectively; hNPCs with a *DCHS1* mutation: 21.6 ± 1.1 μm vs. 29.4 ± 1.4 μm following the saline or metformin treatment, respectively; hNPCs with a *FAT4* mutation: 14.8 ± 1.2 μm vs. 22.0 ± 2.3 μm for the saline or metformin treatment, respectively); speed of migration (control hNPCs: 74.5 ± 2.9 μm/h vs. 73.7 ± 2.7 μm/h following the saline or metformin treatment, respectively; hNPCs with a *DCHS1* mutation: 73.4 ± 2.8 μm/h vs. 86.6 ± 3.2 μm/h for the saline or metformin treatment, respectively; hNPCs with a *FAT4* mutation: 65.5 ± 4.9 μm/h vs. 72.8 ± 6.6 μm/h following the saline or metformin treatment, respectively); percentage of migratory phases (control hNPCs: 37.8 ± 1.3% vs. 37.6 ± 1.5% following the saline or metformin treatment, respectively; hNPCs with a *DCHS1* mutation: 31.4 ± 1.4% vs. 36.1 ± 1.4% following the saline or metformin treatment, respectively; hNPCs with a *FAT4* mutation: 27.8 ± 2.3% vs. 39.2 ± 2.7% following the saline or metformin treatment, respectively). *n* = 54 from three mice and 62 cells from three mice for control hNPCs following the saline or metformin treatment, respectively, *n* = 60 cells from four mice and 65 cells from four mice for hNPCs with a *DCHS1* mutation following the saline or metformin treatment, respectively. *n* = 22 cells from six mice for hNPCs with a *FAT4* mutation following the saline and metformin treatment. *P < 0.05 with a Student's *t*‐test. Individual values and mean ± SEM for all the time‐lapse imaging experiments are shown. Source data are available online for this figure.

**Figure 7:**
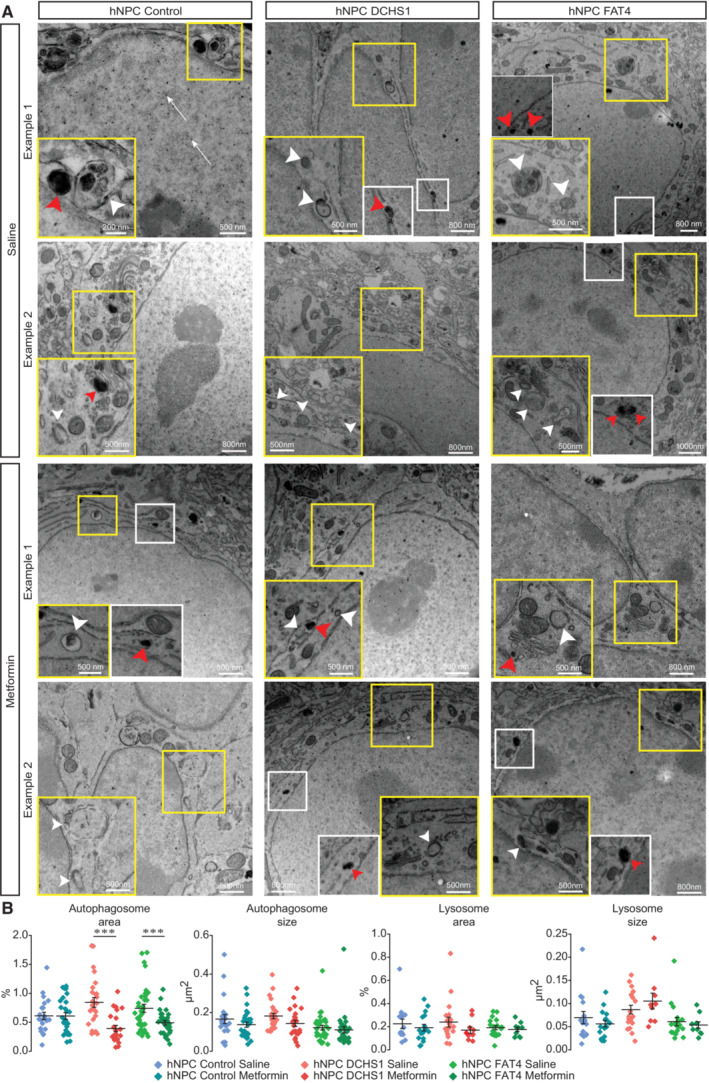
Metformin‐induced rescue of migratory deficits of hNPCs with a *DCHS1* or *FAT4* mutation is associated with changes in autophagy Example of EM images showing migrating hNPCs in the RMS for control hNPCs and for hNPCs with a *DCHS1* or *FAT4* mutation following the saline or metformin treatment. Autophagosomes are indicated by white arrowheads, while lysosomes are indicated by red arrowheads.Quantification of the percentage of the total area and size of autophagosomes and lysosomes for control hNPCs and for hNPCs with a *DCHS1* or *FAT4* mutation following the saline or metformin treatment, respectively. For autophagosomes: *n* = 22 cells from two mice and 28 cells from three mice for control hNPCs following the saline or metformin treatment, respectively; *n* = 26 cells from three mice and 23 cells from two mice for hNPCs with a *DCHS1* mutation following the saline or metformin treatment, respectively; and *n* = 33 cells from five mice and 29 cells from five mice for hNPCs with a *FAT4* mutation following the saline or metformin treatment, respectively. For lysosomes: *n* = 15 cells from two mice and 17 cells from three mice for control hNPCs following the saline or metformin treatment, respectively; *n* = 19 cells from three mice and 10 cells from two mice for hNPCs with a *DCHS1* mutation following the saline or metformin treatment, respectively; and *n* = 18 from five mice and 10 cells from five mice for hNPCs with a *FAT4* mutation following the saline or metformin treatment, respectively. ***P < 0.001 with a one‐way ANOVA followed by a *post hoc* Fisher's LSD test. Individual values and mean ± SEM for all analyzed cells are shown. Source data are available online for this figure.

We next determined whether the deficit in migration observed in cells with a *DCHS1* or *FAT4* mutation can be partially restored by modulating autophagic flux. Metformin, an FDA‐approved drug that activates autophagy by targeting the AMP kinase (AMPK) pathway (Wang *et al*, 2017, 2019; Tan *et al*, 2018; Ma *et al*, 2022), may be an interesting candidate for upregulating autophagy flux in hNPCs with a *DCHS1* or *FAT4* mutation in order to promote their migration. We thus administrated metformin (200 mg/kg, i.p.) for 7–10 days *in vivo* in pups grafted with hNPCs, followed by time‐lapse imaging of hNPC migration in acute brain slices (Fig 6A–C). Metformin injections had no effect on the migratory parameters of control hNPCs (Fig 6C). However, for hNPCs with a *DCHS1* or *FAT4* mutation, the metformin treatment increased the distance of migration up to the level observed with control hNPCs (Fig 6B and C). To determine whether the metformin‐induced increase in the migration of hNPCs with a *DCHS1* or *FAT4* mutation resulted from upregulated autophagy, we performed an EM analysis and quantified the size and overall areas occupied by autophagosomes and lysosomes. Interestingly, the metformin treatment restored the overall area occupied by autophagosomes in hNPCs with a *DCHS1* or *FAT4* mutation, indicating that it had triggered autophagy (Fig 7A and B). Altogether, these results indicate that metformin, by increasing the autophagic flux in hNPCs with a *DCHS1* or *FAT4* mutation, might restore effective migration.

## Discussion

Altogether, our results show that the *DCHS1* and *FAT4* mutations impaired autophagy, leading to alterations in paxillin recycling and the abnormal migratory behavior of xenotransplanted hNPCs *in vivo*. Alterations in autophagy may arise either from an affected gene expression profile of autophagy‐related and/or lysosomal genes or from transcription‐independent changes in the effectiveness and dynamics of the process itself. We recently showed that autophagy is a key homeostatic process that adapts the migratory behavior of neuronal precursors in response to different migration‐promoting or migration‐inhibiting molecular cues (Bressan *et al*, 2020; Bressan & Saghatelyan, 2020). Our scRNA‐seq analysis also shows that the vast majority of the autophagy‐related and lysosomal genes analyzed remained unchanged in hNPCs derived from PH patients with a *DCHS1* or *FAT4* mutation. The only gene that was altered in cells with a *FAT4* or *DCHS1* mutation was *MAP1LC3A*, which is the predominant form of *MAP1LC3* in the brain (He *et al*, 2003). The changes were, however, very subtle and opposing, increasing in hNPCs with a *FAT4* mutation and decreasing in hNPCs with a *DCHS1* mutation compared to the control cells. Interestingly, however, the protein levels of lipidated MAP1LC3 increased in cerebral organoids derived from hNPCs with a *DCHS1* or *FAT4* mutation and *in vivo* in xenotransplanted hNPCs with a *DCHS1* or *FAT4* mutation. Given that (i) autophagy is activated to adapt the responses of migrating cells to different molecular cues (Bressan *et al*, 2020; Bressan & Saghatelyan, 2020), (ii) the genes involved in autophagy or lysosomal biogenesis were largely unaffected by the *DCHS1* or *FAT4* mutations, and (iii) blocking autophagosome and lysosome fusion only affected the migration of control hNPCs but not of hNPCs with a *DCHS1* or *FAT4* mutation, the changes in autophagy described here reflect a transcription‐independent homeostatic adaptation of hNPCs to the altered migratory behavior of cells.

Interestingly, it has been previously suggested that DCHS1 and FAT4 may regulate cell proliferation, migration, and polarity in a transcription‐independent manner. These two cadherins are involved in bone morphogenesis in the early pre‐chondrogenic mesenchyme, cell proliferation during early chondrogenesis, and the collective migration of murine facial branchiomotor neurons that is independent of the activation of the transcriptional targets of FAT4 and DCHS1 such as the Hippo pathway transcriptional co‐activators Yap and Taz (Zakaria *et al*, 2014; Kuta *et al*, 2016; Mao *et al*, 2016). Furthermore, in addition to the lack of activation of Yap and Taz, FAT4‐ and DCHS1‐mediated effects on cell proliferation in developing vertebrae occur without any changes in the overall transcriptional network required for early chondrogenesis (Kuta *et al*, 2016). The lack of FAT4 and DCHS1 also induces a delay in the tangential migration of murine facial branchiomotor neurons during embryogenesis, without affecting the transcription of genes involved in cell migration and axonal patterning and guidance (Zakaria *et al*, 2014). These transcription‐independent effects of DCHS1 and FAT4 can occur through alterations in the intracellular localization of organelles and cytoskeleton dynamics. The lack of FAT4 or DCHS1 results in the random localization of the Golgi apparatus and the appearance of shortened, less‐organized actin filaments in migrating branchiomotor neurons (Zakaria *et al*, 2014). Similarly, in embryonic zebrafish and Drosophila wings, the loss of FAT or DCHS affects cell polarity and microtubule dynamics either by controlling the subcellular localization of the PAR‐1 kinase (Harumoto *et al*, 2010) or by directly interacting with the Ttc28 protein (Chen *et al*, 2018). In Drosophila, these cadherins also control the collective migration of larval epidermal cells by directing lamellipodia formation through phosphatidylinositol 3‐kinase (Arata *et al*, 2017) and by remodeling gap junctions and localizing microtubules in the epidermis independently of Yorkie‐mediated transcription (Marcinkevicius & Zallen, 2013). These transcription‐independent changes in cellular polarity, organelle distribution, and cytoskeleton dynamics may disrupt neuronal migration and trigger an adaptive cellular response to cope with altered cell migration. We have previously shown that autophagy, a major homeostatic pathway in migrating cells, is dynamically regulated in response to migration‐promoting or migration‐inhibiting cues to regulate cell migration by recycling focal adhesion molecules (Bressan *et al*, 2020; Bressan & Saghatelyan, 2020). Both FAT4 and DCHS1 can regulate autophagy (Wei *et al*, 2019; Sun & Zhang, 2020). FAT4 activation promotes autophagy in lung cancer cells (Yang *et al*, 2022), whereas its downregulation alters autophagic flux and leads to the accumulation of autophagosomes in photoreceptor neurons in Drosophila, thereby affecting cellular homeostasis (Napoletano *et al*, 2011). These effects are reminiscent of our observations, showing that altered autophagic flux was altered in hNPCs with a *DCHS1* or *FAT4* mutation.

In addition to these transcription‐independent changes induced by DCHS1 and FAT4, several other migration‐associated genes have been shown to be affected in organoids derived from hNPCs with a *DCHS1* or *FAT4* mutation (Klaus *et al*, 2019). While these genes may affect neuronal migration in their own specific way, neuronal migration relies on the involvement of multiple pathways. It is thus conceivable that the modulation of migration‐promoting pathways that are not transcriptionally affected by PH‐inducing genetic mutations may be used to rescue, at least partially, the migratory deficits of hNPCs with a *DCHS1* or *FAT4* mutation. Given that autophagy is dynamically regulated in migrating cells to cope with the pace and periodicity of neuronal migration (Bressan *et al*, 2020) and that changes in autophagy are largely transcription‐independent, we hypothesized that upregulating autophagic flux by promoting the fusion of autophagosomes with lysosomes and triggering focal adhesion molecules recycling may, at least partially, rescue the migratory deficits in hNPCs with a *DCHS1* or *FAT4* mutation. Interestingly, a 7‐ to 10‐day treatment with metformin decreased the accumulation of autophagosomes and, more importantly, restored the migration of hNPCs with a *DCHS1* or *FAT4* mutation, indicating that the defective migration of hNPCs derived from PH patients may be rescued by modulating autophagy. It remains to be determined how paxillin recycling and LC3/paxillin co‐localizations are affected by metformin treatment. Metformin has previously been described as an AMPK‐dependent autophagy enhancer due to its ability to activate AMPKα2 (Wang *et al*, 2019). Metformin, at low doses, also inhibits mTORC1 signaling by activating lysosomal AMPK (Ma *et al*, 2022). In line with our results are observations showing that other forms of PH caused by *NEDD4L* mutations deregulate mTORC signaling (Broix *et al*, 2016). Treating *NEDD4L* mutant cells with rapamycin, an mTORC inhibitor and autophagy activator, partially rescues the migratory capacity of the cells and corrects the positioning of these cells in the cortical plate (Broix *et al*, 2016). Other forms of PH linked to mutations in filamin 1 (FLN1), endothelin‐converting enzyme‐2 (ECE2), and secreted LGALS3BP, among others, have been described (Buchsbaum & Cappello, 2019; Buchsbaum *et al*, 2020; Kyrousi *et al*, 2021). The role of autophagy in these forms of PH remains to be determined. However, our data, together with previous observations (Broix *et al*, 2016), indicate that autophagy plays an important role in the abnormal migratory phenotypes of hNPCs, at least in some forms of PH, and that modulating this pathway may be a promising avenue for rescuing cellular migration and neuronal positioning defects in these patients.

## Materials and Methods

### Animals

The experiments were performed using Rag1^−/−^ pups injected at postnatal days 3–5 (P3‐5). All experiments were approved by the Université Laval animal protection committee. The mice were housed one to five per cage on a 12 h light/dark cycle at a constant temperature (22°C) with food and water *ad libitum*.

### iPSC cultures

iPSCs were generated as previously described (Klaus *et al*, 2019). We used two control and two patient‐derived lines with a mutation in DCHS1 or FAT4 that were previously described and characterized by Klaus *et al* (2019). The control line 1 is Riken HPS0076: 409B2 (https://cellbank.brc.riken.jp/cell_bank/CellInfo/?cellNo=HPS0076), while the control line 2 is https://hpscreg.eu/cell‐line/ISFi001‐A. We also used an additional control line (SK0019‐002) that was previously described (Deneault *et al*, 2019; Zaslavsky *et al*, 2019). This line was used for miRNA‐induced *DCHS1* or *FAT4* knock‐down. The generation of iPSC lines was performed with patient consent (Klaus *et al*, 2019), and xenotransplantation of hNPCs was approved (protocol number 2018‐239) by the ethics committee of the “Centre intégré universitaire de santé et de services sociaux de la Capitale‐Nationale,” Quebec City, Canada.

iPSCs were cultured in mTeSR1 medium (Stem Cell Technologies, Canada) on hESC‐Qualified Matrigel^®^ (Corning) following the manufacturer's protocol (Stem Cell Technologies). Cell passaging was performed without manual scraping using ReleSR™ (Stem Cell Technologies) for 4 min to select the colonies.

### Generation of hNPCs

hNPCs were generated in STEMdiff™ Neural System Medium in AggreWell™ 800 microwell culture plates (Stem Cell Technologies) using the embryoid body (EB) protocol. Briefly, iPSC colonies were detached and were cultured in AggreWell™ 800 plates for 5 days in STEMdiff™ Neural Induction Medium in the presence of SMADi (Stem Cell Technologies). On Day 5, the EB was seeded in a 6‐well plate coated with Matrigel™ hESC‐Qualified Matrix in STEMdiff™ Neural Induction Medium and SMADi. The medium was replaced daily. On Day 12, neural rosettes were selected using STEMdiff™ Neural Rosette Selection Reagent (Stem Cell Technologies) according to the manufacturer's protocol and were replated in STEMdiff™ Neural Induction Medium and SMADi in the wells of a 6‐well plate. The wells had been previously coated with Matrigel™ hESC‐Qualified Matrix (Corning). On Day 17, the cells were considered to be ready for passage, and 600,000 cells per well were plated in STEMdiff™ Neural Progenitor Medium in a 24‐well plate. The wells had been previously coated with Matrigel™ hESC‐Qualified Matrix (Corning). Subsequent passages were made once a week using Accutase^®^ solution when the cells reached confluence. Y‐27632 (10 μM) was added during passaging.

### Nucleofection of microRNA against DCHS1 and FAT4

hNPCs were nucleofected with previously characterized and validated miRNAs against *DCHS1* and *FAT4* (Klaus *et al*, 2019). The hNPCs were placed in a Nucleofector^®^ (Lonza) and were nucleofected using a P3 Primary Cell 4D X Kit L (Lonza, V4XP‐3012) according to the manufacturer's protocol using the DS113 program. For each reaction, 5 μg of plasmid DNA was used for about 5 × 10^6^ cells. The NPCs were cultured for at least 3–5 days following the nucleofection before being xenotransplanted.

### Organoid preparation

Cerebral organoids were prepared as previously described (Klaus *et al*, 2019). Briefly, organoids were kept in 10‐cm dishes on an orbital shaker at 37°C in a 5% CO_2_/ambient oxygen atmosphere. The medium was changed every 3–4 days.

### Immunostaining of organoid sections

Frozen organoid sections (15‐μm‐thick) were cut using a cryostat (Leica). The sections were incubated with the following primary antibodies: anti‐MAP1ALC3A (1:100, Abgent, USA, AP1805a, RRID:AB_2137587), anti‐MAP1ALC3B (1:200, NB100‐2220, RRID:AB_10003146), and anti‐DCX (1:1,000, Millipore, USA, AB2253, RRID:AB_1586992). Images were acquired using an inverted Zeiss microscope (LSM 700, AxioObserver) equipped with a 40× objective (NA: 1.4).

### Western blotting

Western blotting was performed as described previously (Bressan *et al*, 2020). The concentration of total protein was measured using the Bradford assay (BioRad). Proteins were separated on 16% NuPage gels (Invitrogen) in SDS running buffer and were transferred to nitrocellulose membranes (Life Technologies). The following primary antibodies were used: anti‐LC3B (1:1,000, Novus), anti‐paxillin (1:1,000, BD Biosciences, 610051, RRID: AB_397463), anti‐p62/SQSTM1 (1:500, ProteinTech, 18420‐1‐AP, RRID: AB_10694431) and anti‐GAPDH (1:5,000, Thermo Fisher Scientific, MA5‐15738, RRID: AB_10977387).

### *In vivo* xenotransplantation

One day prior to grafting, the hNPCs were infected with a GFP‐expressing lentivirus (SignaGen) by adding the virus to the culture medium followed by centrifuging at 1,000 *g* for 1 h (Topol *et al*, 2015). The following day, the cells were detached using Accutase™ solution and were resuspended in DMEM/F12 supplemented with Y‐27632 (10 μM) at a density of 100,000 cells/μl. The pups were anesthetized on ice (5 to 10 min), and 100,000 hNPCs were injected in the SVZ at each of the following coordinates (with respect to the lambda): AP 3.05 mm, ML 0.85 mm, and DV 1.9 to 1.5 mm (100 nl/0.1 mm).

### Immunolabeling of xenotransplanted hNPCs

Two weeks following the hNPC grafting, the pups were perfused transcardially with ice‐cold sodium phosphate‐buffered saline (0.1 M PBS, pH 7.4) followed by ice‐cold 4% PFA to which 0.1% glutaraldehyde had been added. The brains were extracted and were post‐fixed overnight. Sagittal brain sections (50‐μm‐thick) were cut with a vibratome (VT1200 S; Leica, Germany) and were incubated with the following primary antibodies diluted in 0.2% Triton X‐100 with 4% milk: anti‐LC3B (1:500, Novus, RRID:AB_10003146), anti‐paxillin (1:100, BD Biosciences, 610051, RRID: AB_397463), anti‐GFP (1:1,000, Avés, GFP‐1020, RRID: AB_10000240), anti‐Atg12 (1:500, Abcam, #ab155589), and anti‐Lamp1 (5 μg, R&D system, AF4800, RRID: AB_1026176). Images were acquired using an upright Zeiss microscope (LSM 700, AxioObserver) equipped with a 63× oil immersion objective (NA: 1.4). To quantify LAMP1, LC3, ATG12, and paxillin immunolabeling, the region of interest (ROI) was drawn manually on each optical section of individual cells based on the GFP fluorescence using ImageJ software. For LC3, Atg12, and LAMP1, the quantification was performed in the entire cell including the cell soma, leading process, and growth cone. The mean fluorescent intensity in each ROI was obtained in ImageJ and was summed across all optical sections to obtain the level of fluorescent intensity for individual cells. For paxillin quantifications, given that paxillin recycling is higher at the distal processes of migrating cells comprising the growth cone and that autophagy specifically affects paxillin recycling in the distal processes of mouse neuroblasts migrating in the RMS (Bressan *et al*, 2020), we focused on an analysis of paxillin immunolabeling in the distal processes of xenotransplanted hNPCs. For paxillin immunolabeling, we subtracted the background fluorescent intensity measured near GFP^+^ distal processes from the fluorescent signal.

### Time‐lapse imaging

To analyze hNPC migration, the mice were sacrificed and acute sections were prepared as described previously (Bakhshetyan & Saghatelyan, 2015). Briefly, the mice were anesthetized with ketamine (100 mg/kg) and xylazine (10 mg/kg) and were perfused transcardially with modified oxygenated artificial cerebrospinal fluid (ACSF). The brains were then quickly extracted, and 250‐μm‐thick sections were cut using a vibratome (HM 650 V; Thermo Fisher Scientific). The sections were kept at 37°C under oxygenation in ACSF. Time‐lapse images were acquired using a BX61WI (Olympus) upright microscope equipped with a 40× water immersion objective (NA = 0.8) and a CCD camera (CoolSnap HQ). A mercury arc lamp was used as the illumination source (Olympus). Images were acquired every 30 s for 1 h with multiple z stacks. Migratory parameters (distance of migration, speed, and percentage of migratory phases) were obtained using Imaris8 (Biplane) and OriginLab software, as described previously (Bressan *et al*, 2020). We used bafilomycin A1 (Cayman Chemical) and metformin (Sigma) to modulate autophagy. A bafilomycin A1 stock solution was prepared in DMSO and was then added to the ACSF at a 4 μM final concentration. We first recorded hNPC migration under baseline conditions in ACSF supplemented with DMSO followed by time‐lapse imaging in the presence of 4 μM bafilomycin A1. Metformin was injected intraperitoneally (i.p.) at final concentration of 200 mg/kg of body weight of the animal. The treatments were performed daily starting 7 days post‐grafting of hNPCs and lasted for 7–10 days. Control mice were injected with the same volume of saline.

### Electron microscopy

Two weeks following NPC grafting, the pups were perfused transcardially with ice‐cold sodium phosphate‐buffered saline (PBS 0.1 M, pH 7.4) followed by ice‐cold 4% PFA to which 0.1% glutaraldehyde had been added. The brains were extracted and post‐fixed overnight. Sagittal brain sections (50‐μm‐thick) were cut with a vibratome (VT1200 S; Leica, Germany) and were sequentially incubated in a 0.1 M sodium borohydride solution for 30 min and in a blocking solution composed of 2% normal goat serum and 0.5% gelatin for 1 h. They were then incubated with a rabbit anti‐GFP antibody (1:1,000, 24 h, room temperature‐RT, Novus, NB 600‐308) and with a Nanogold® goat anti‐rabbit antibody (1:50, 24 h, 4°C, Nanoprobe, #2004, RRID:AB_2631182). The immunogold staining was amplified using an HQ Silver Enhancement Kit (Nanoprobe, #2012). The sections were then incubated for 1 h at room temperature with a solution containing equal volumes of 3% potassium ferrocyanide (MP Biomedicals, LLC, N152560) and 4% aqueous osmium tetroxide (EMS) in 0.1 M phosphate buffer (PB) following which they were incubated for 20 min in a 1% thiocarbohydrazide solution (EMS) and then in a 2% aqueous osmium tetroxide solution for 30 min following which they were dehydrated, embedded in Durcupan, and placed between Aclar sheets (EMS) at 55°C for 3 days. Ultrathin sections (50‐nm‐thick) were obtained with an ultramicrotome (model EM UC7, Leica) and were collected on bare 150‐mesh copper grids. Images were acquired using a transmission electron microscope (Tecnai 12; Philips Electronic, 100 kV) equipped with an integrated digital camera (XR‐41, Advanced Microscopy Techniques Corp). GFP^+^ cell bodies containing immunogold particles were randomly selected along the RMS. Autophagosomes and lysosomes were identified in GFP^+^ cell bodies according to well‐established criteria (Eskelinen, 2008) and were measured using ImageJ software.

### Statistical analysis

Data are expressed as mean ± SEM. The individual values of all experiments are also shown in the corresponding figures. The investigator was not blinded to the experimental conditions, and no animal was excluded from analysis. Statistical significance was determined using an unpaired two‐sided Student's *t*‐test or a one‐way ANOVA followed by a *post hoc* Fisher's LSD test, depending on the experiment, as indicated. Equality of variance for the unpaired *t*‐test was verified using the F‐test. The exact values of *n* and its representation (cells, animals) for all experiments are indicated in the Figure Legends. The levels of significance were as follows: *P < 0.05, **P < 0.01, and ***P < 0.001.

## Author contributions

**Cedric Bressan:** Conceptualization; data curation; formal analysis; validation; investigation; visualization; methodology; writing – original draft; writing – review and editing. **Marta Snapyan:** Data curation; formal analysis; validation; investigation; visualization; methodology. **Marina Snapyan:** Data curation; formal analysis; validation; investigation; visualization; methodology. **Johannes Klaus:** Resources. **Francesco di Matteo:** Resources. **Stephen P Robertson:** Resources; validation; writing – review and editing. **Barbara Treutlein:** Resources; validation. **Martin Parent:** Formal analysis; supervision; funding acquisition; validation; investigation; visualization; methodology; writing – review and editing. **Silvia Cappello:** Resources; data curation; supervision; funding acquisition; validation; investigation; visualization; methodology; writing – review and editing. **Armen Saghatelyan:** Conceptualization; resources; data curation; formal analysis; supervision; funding acquisition; validation; investigation; visualization; methodology; writing – original draft; project administration; writing – review and editing.

## Disclosure and competing interests statement

The authors declare that they have no conflict of interest.

## Supporting information



Movie EV1

Movie EV2

Source Data for Figure 1

Source Data for Figure 2

Source Data for Figure 3

Source Data for Figure 4

Source Data for Figure 5

Source Data for Figure 6

Source Data for Figure 7

## Data Availability

This study includes no data deposited in external repositories.
