# Supplementary figures and images for: Metformin rescues migratory deficits of cells derived from patients with periventricular heterotopia
Source: EMBO Mol Med. 2023 Aug 23;15(10):e16908. doi: 10.15252/emmm.202216908 (PMC10565636; doi:10.15252/emmm.202216908)

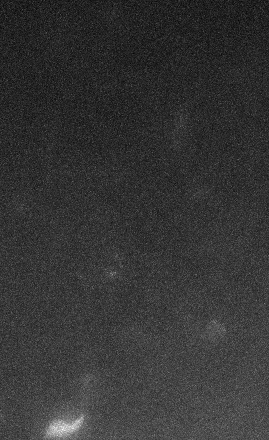

Supplement: Supplementary file 3 — Source Data for Figure 1 [file EMMM-15-e16908-s003.zip › Figure 1/1C/Example of time-lapse imaging - Control.tif]

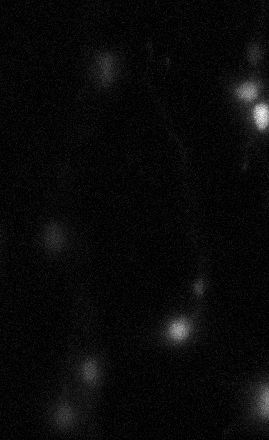

Supplement: Supplementary file 3 — Source Data for Figure 1 [file EMMM-15-e16908-s003.zip › Figure 1/1C/Example of time-lapse imaging - DCHS1.tif]

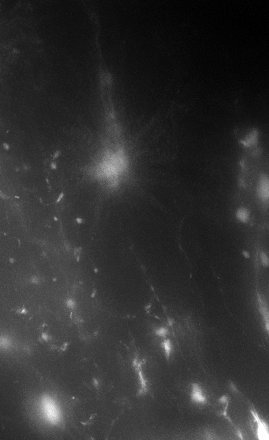

Supplement: Supplementary file 3 — Source Data for Figure 1 [file EMMM-15-e16908-s003.zip › Figure 1/1C/Example of time-lapse imaging - FAT4.tif]

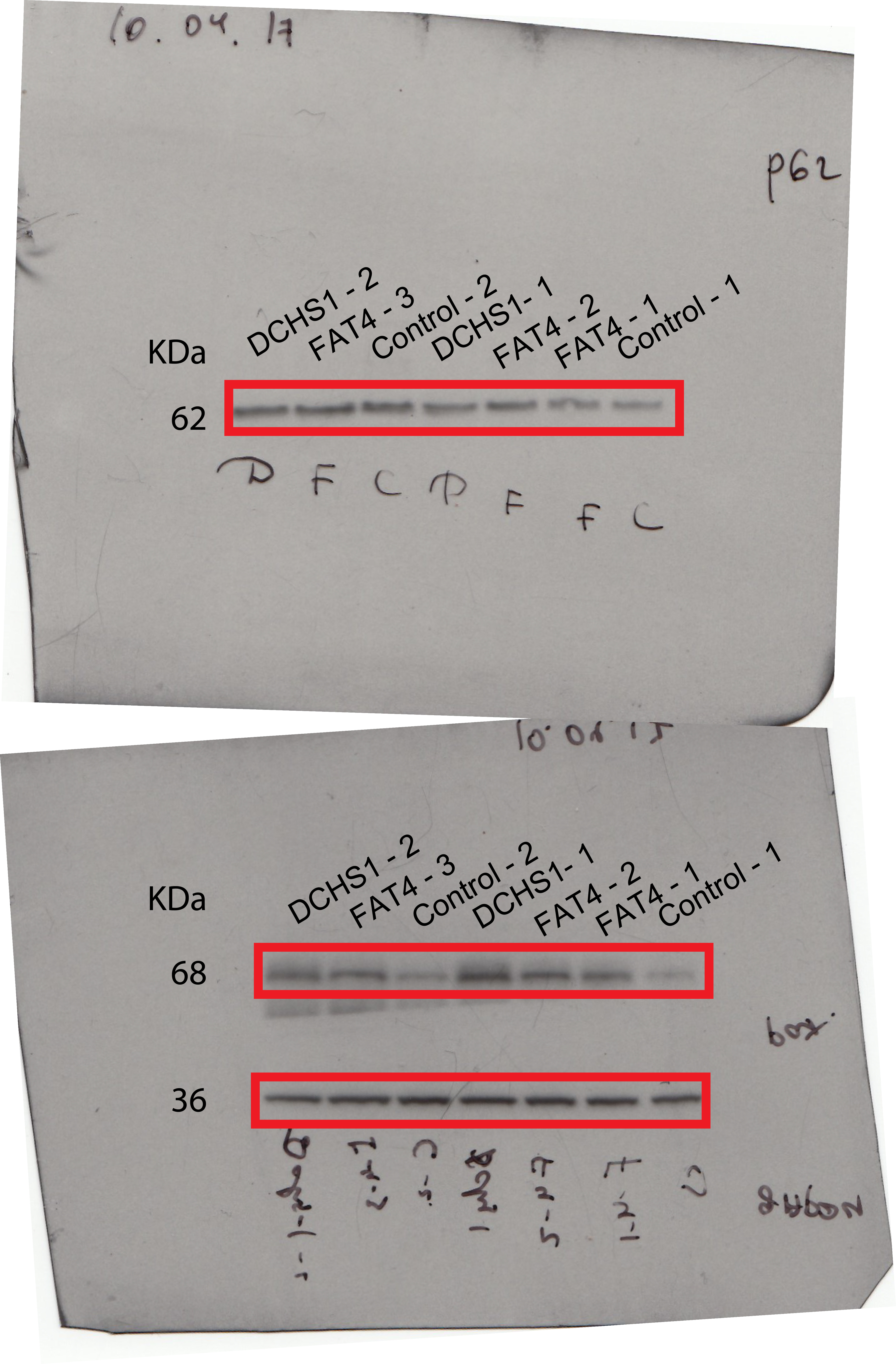

Supplement: Supplementary file 4 — Source Data for Figure 2 [file EMMM-15-e16908-s008.zip › Figure 2/2B/Gel100417.jpg]

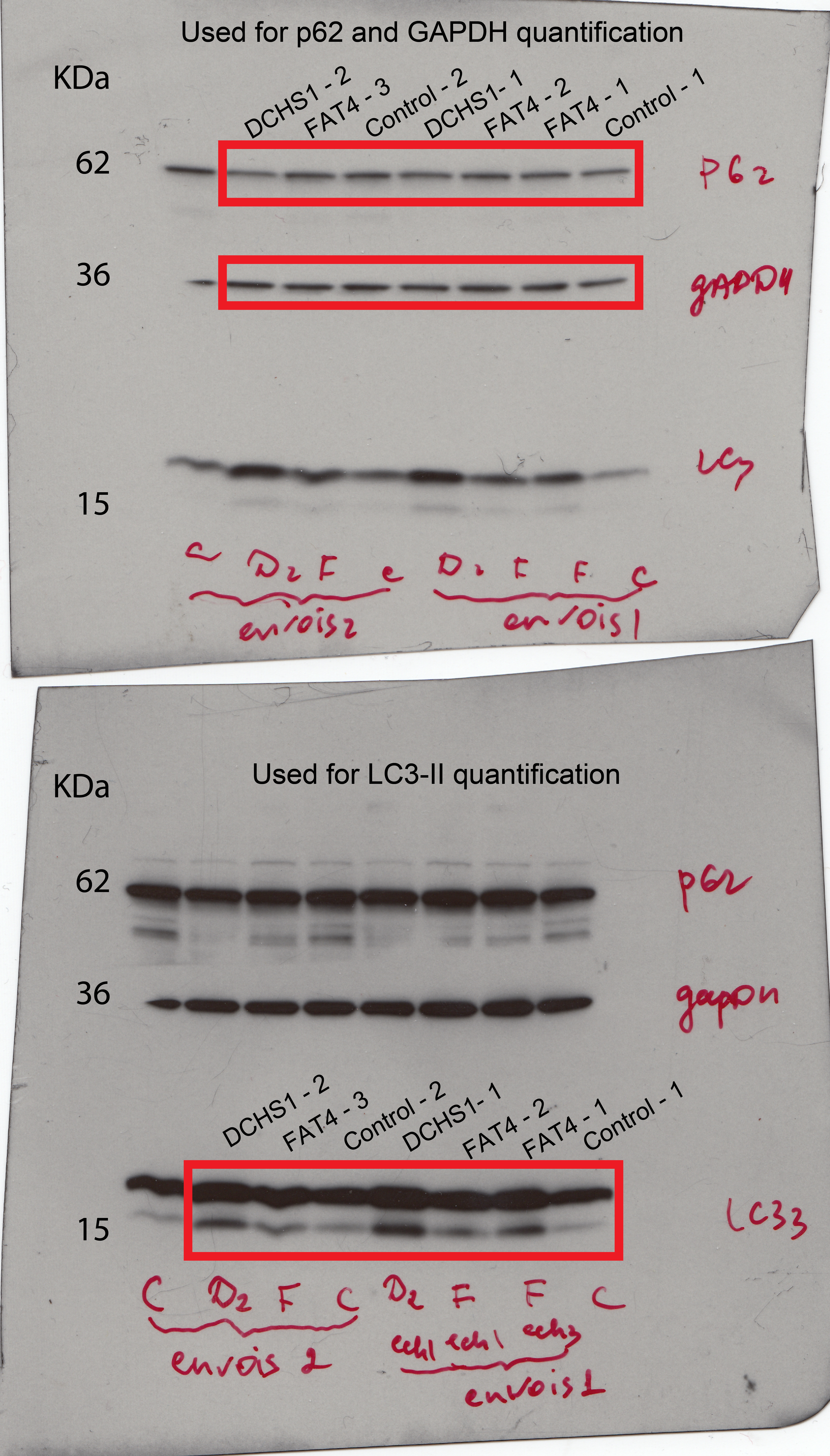

Supplement: Supplementary file 4 — Source Data for Figure 2 [file EMMM-15-e16908-s008.zip › Figure 2/2B/Gel280317.jpg]

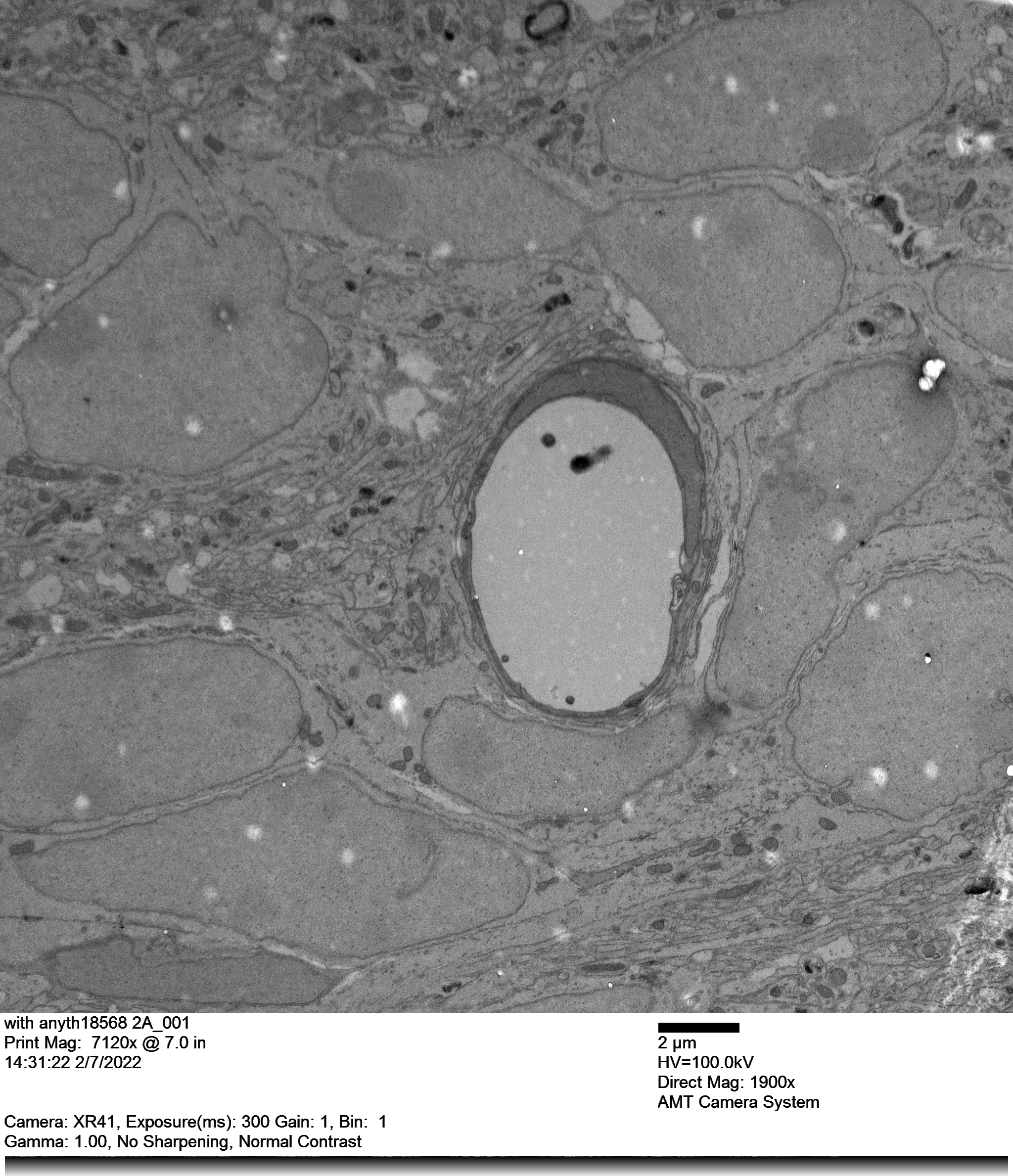

Supplement: Supplementary file 6 — Source Data for Figure 4 [file EMMM-15-e16908-s010.zip › Figure 4/4B/hNPC Control-image1.TIF]

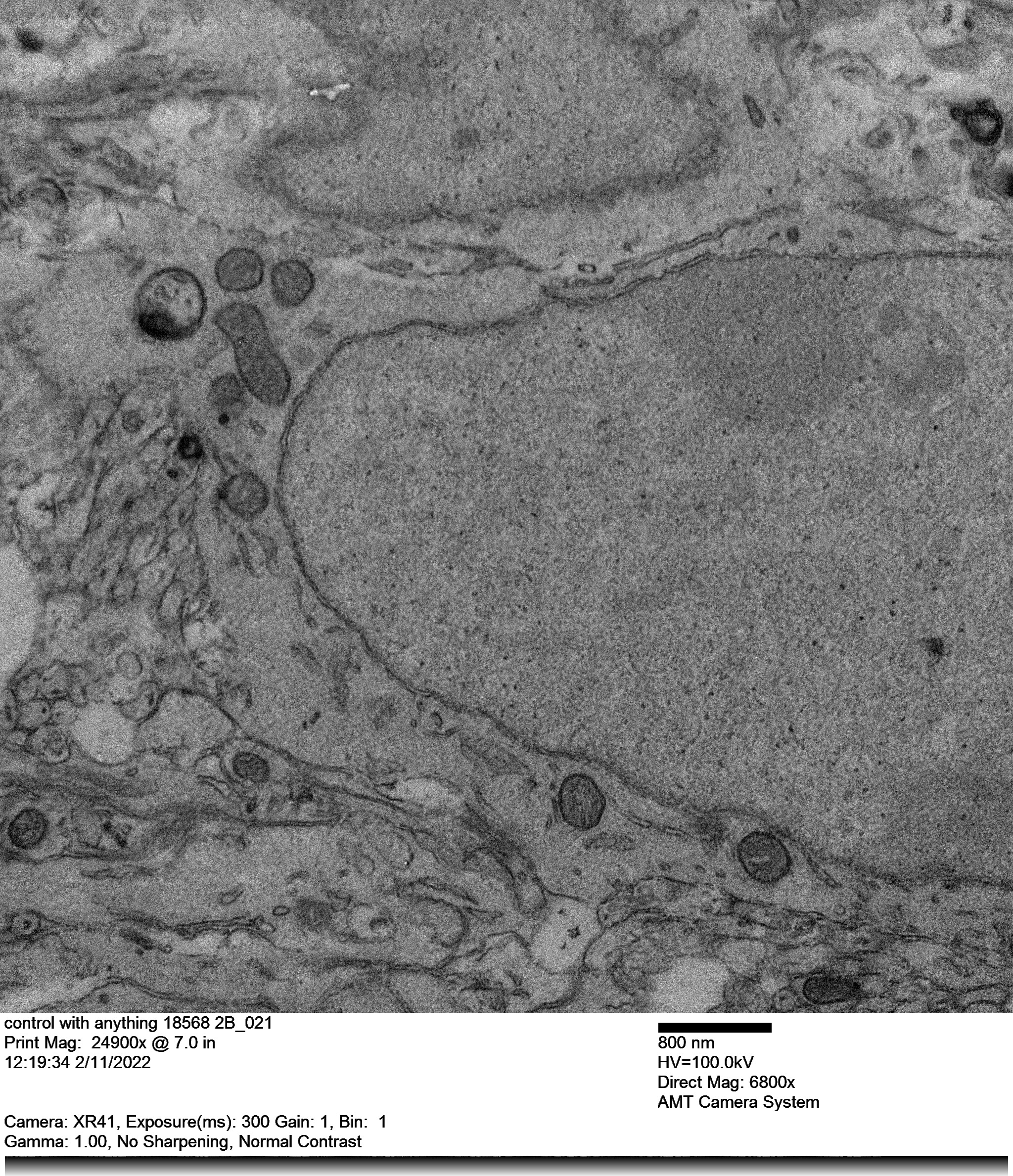

Supplement: Supplementary file 6 — Source Data for Figure 4 [file EMMM-15-e16908-s010.zip › Figure 4/4C/hNPC Control-example1.TIF]

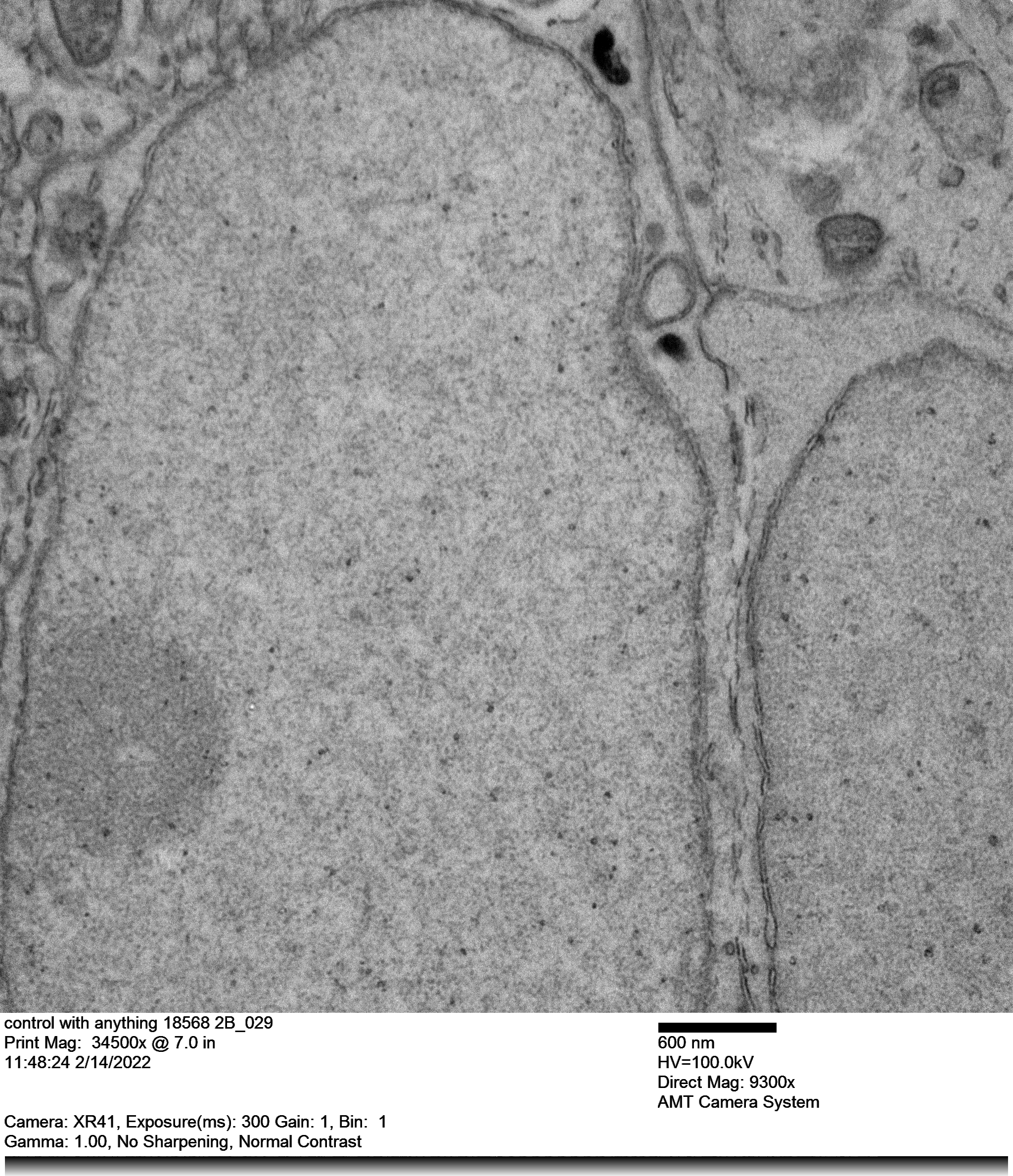

Supplement: Supplementary file 6 — Source Data for Figure 4 [file EMMM-15-e16908-s010.zip › Figure 4/4C/hNPC Control-example2.TIF]

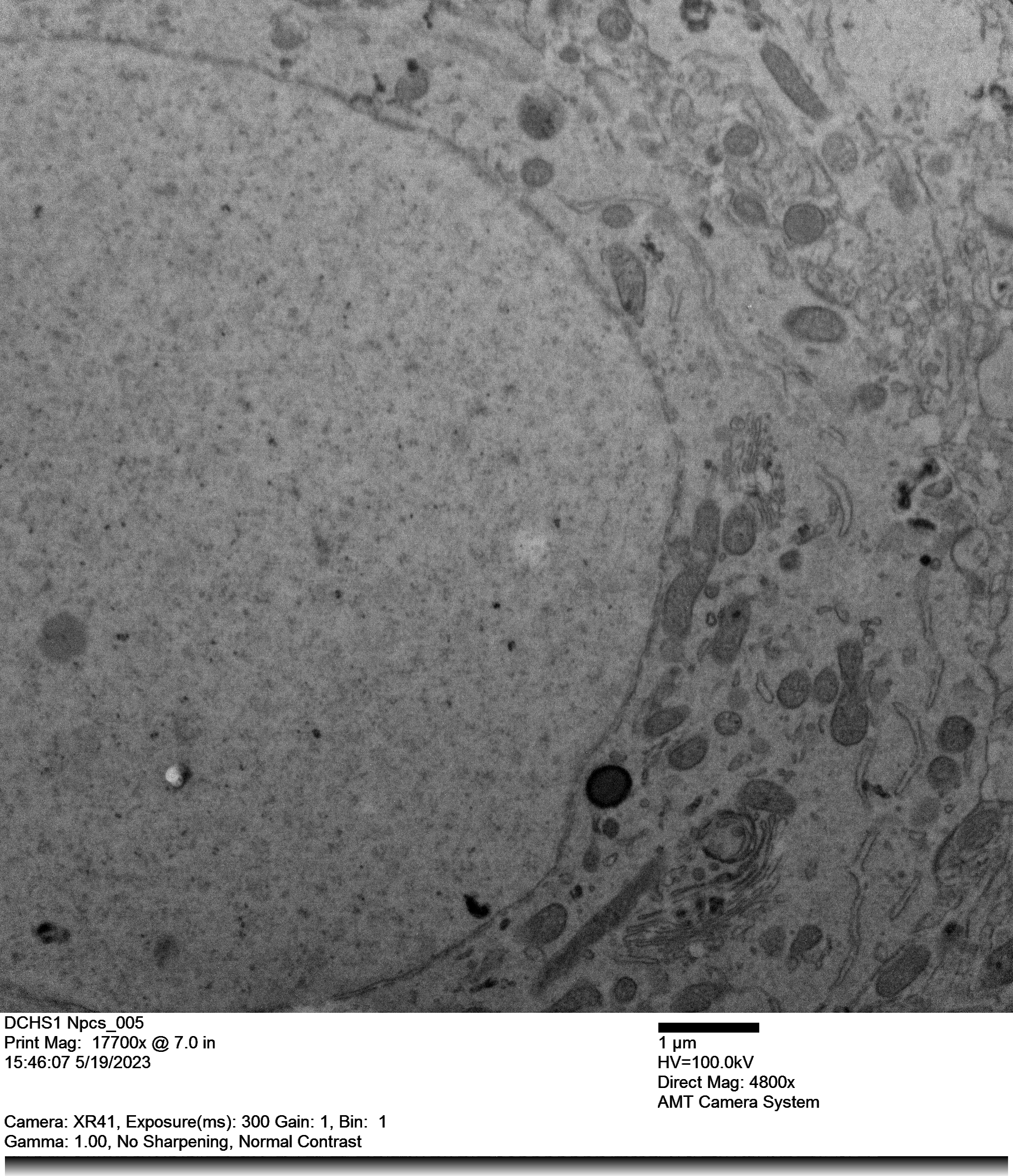

Supplement: Supplementary file 6 — Source Data for Figure 4 [file EMMM-15-e16908-s010.zip › Figure 4/4C/hNPC DCHS1-example1.TIF]

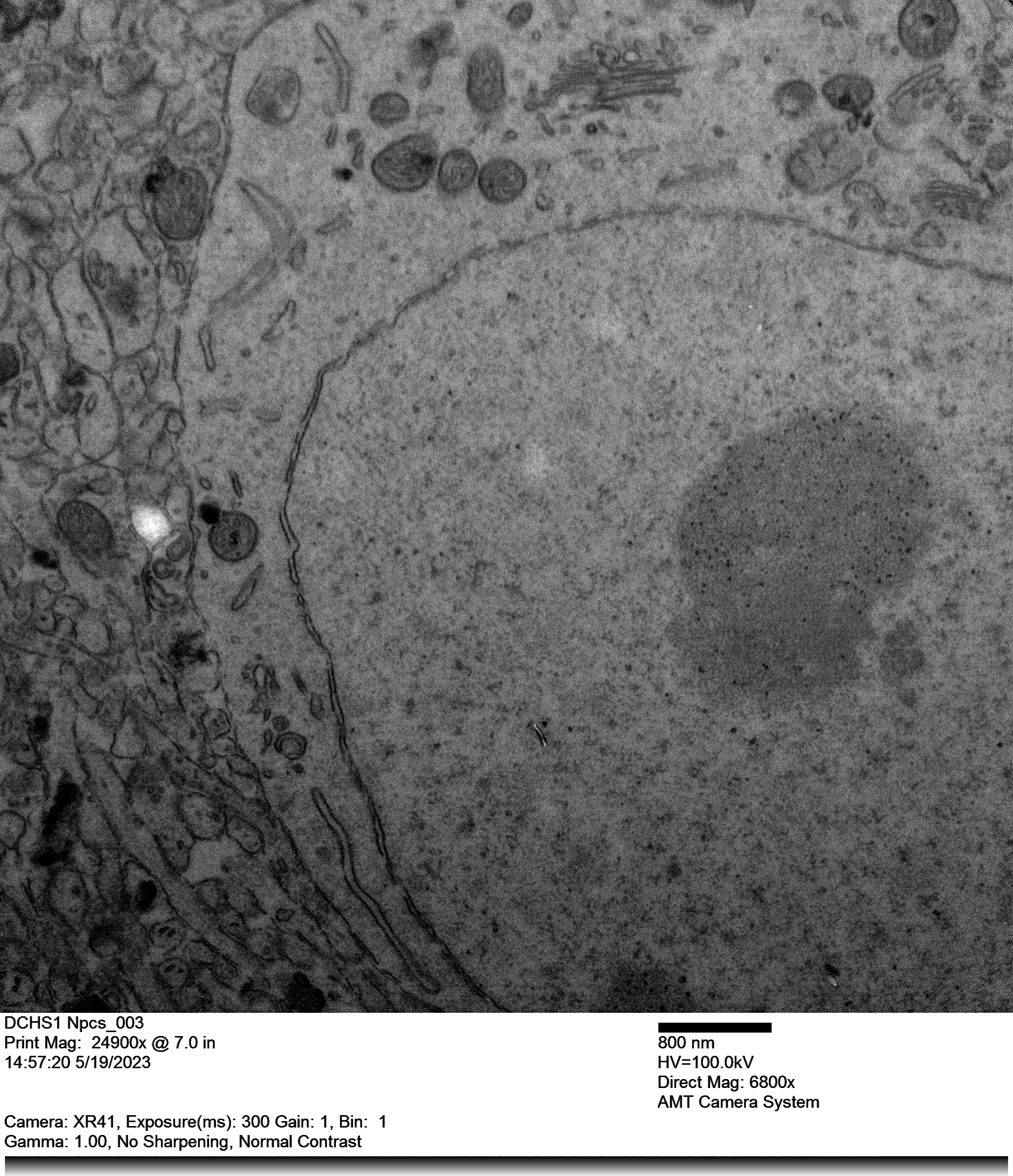

Supplement: Supplementary file 6 — Source Data for Figure 4 [file EMMM-15-e16908-s010.zip › Figure 4/4C/hNPC DCHS1-example2.TIF]

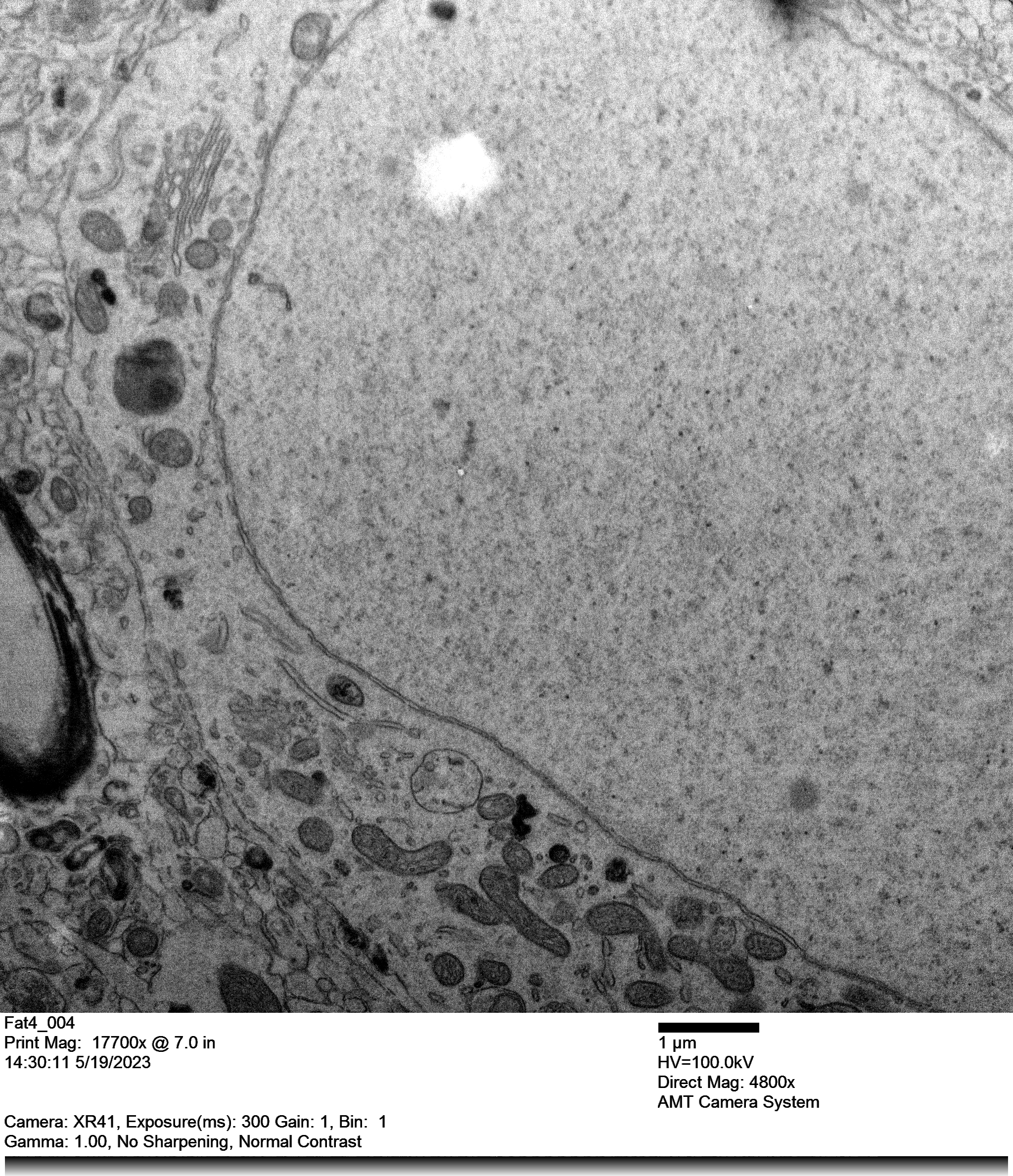

Supplement: Supplementary file 6 — Source Data for Figure 4 [file EMMM-15-e16908-s010.zip › Figure 4/4C/hNPC FAT4-example1.TIF]

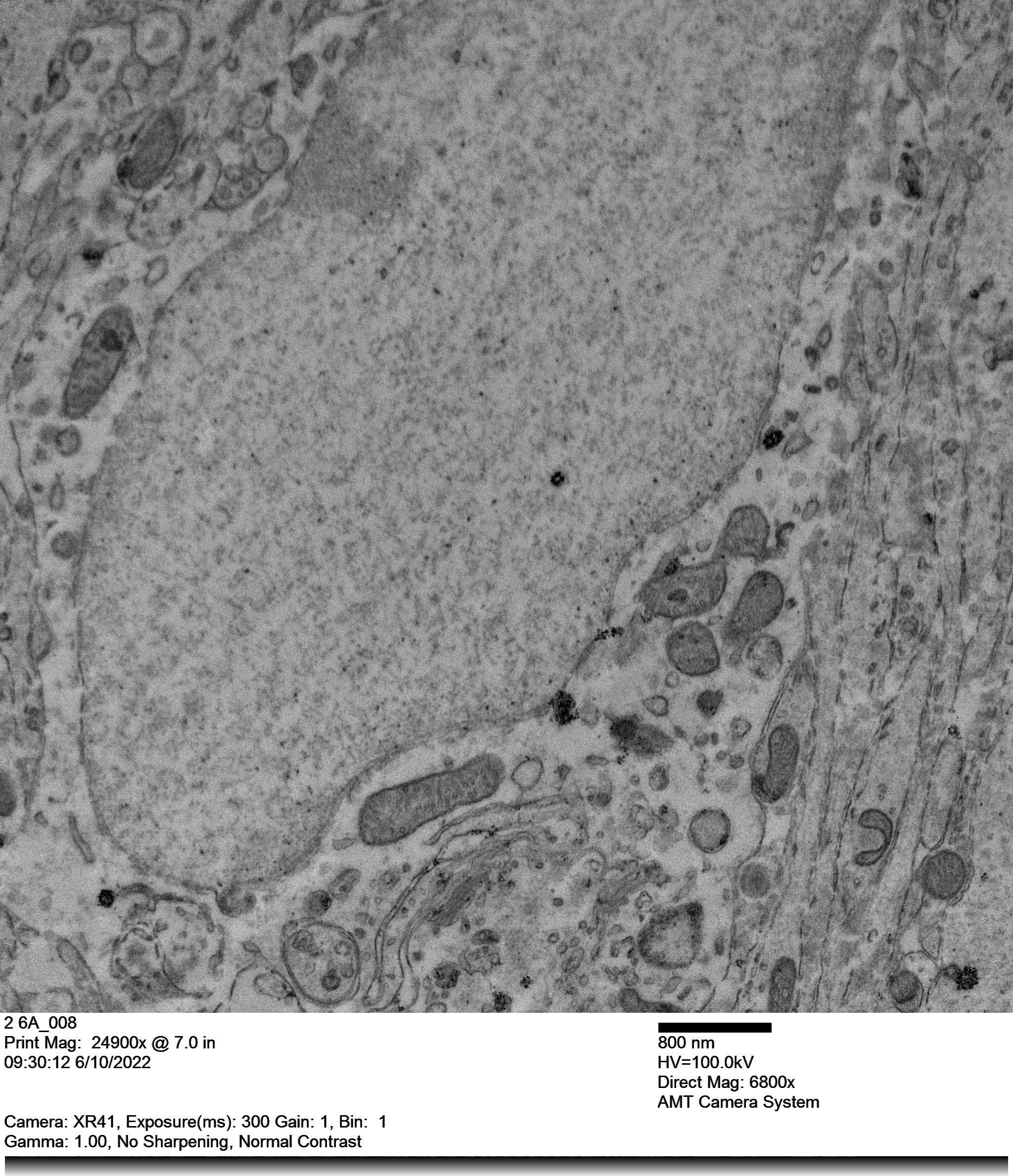

Supplement: Supplementary file 6 — Source Data for Figure 4 [file EMMM-15-e16908-s010.zip › Figure 4/4C/hNPC FAT4-example2.TIF]

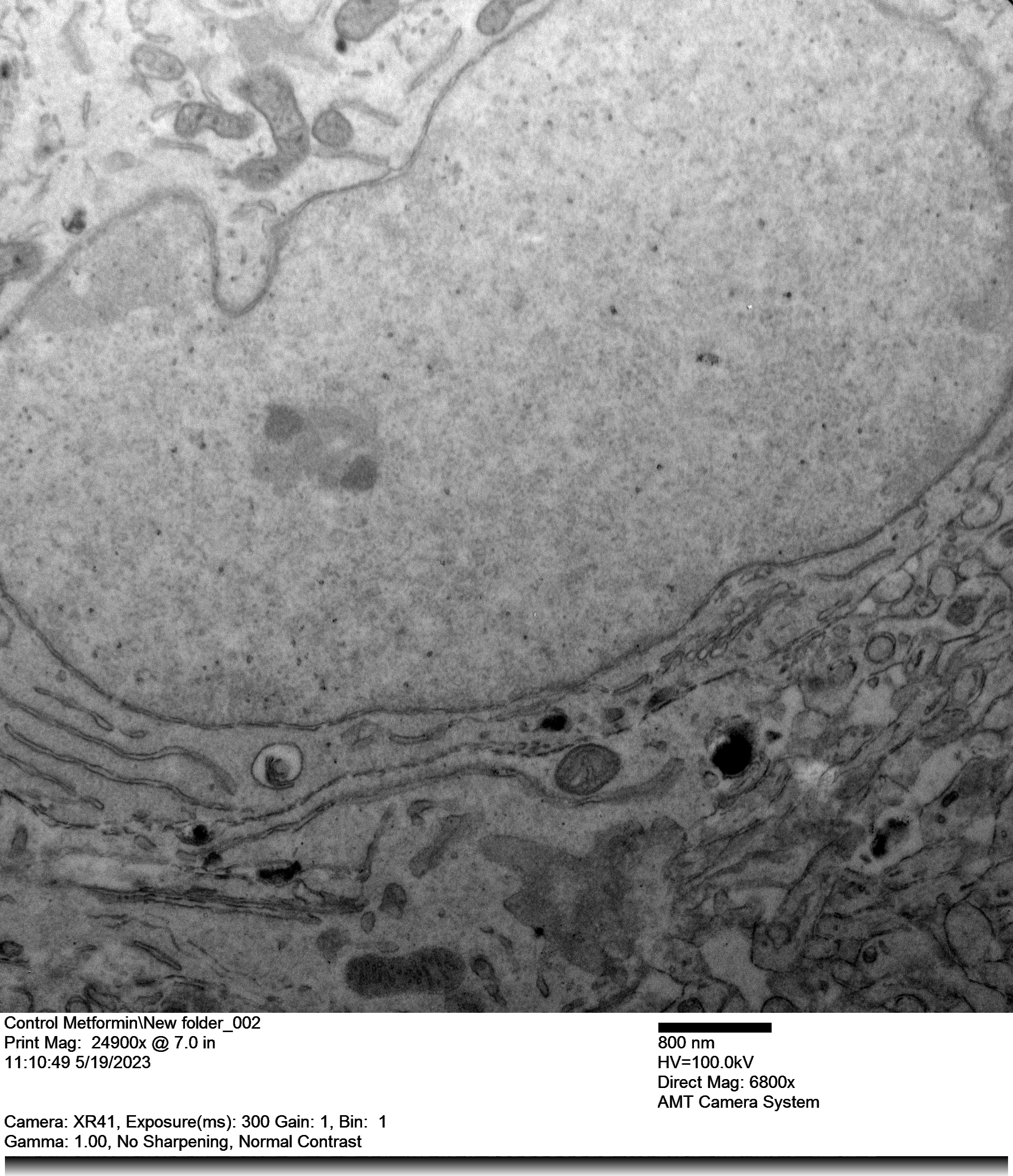

Supplement: Supplementary file 9 — Source Data for Figure 7 [file EMMM-15-e16908-s004.zip › Figure 7/7A/hNPC Control Metformin - example1.TIF]

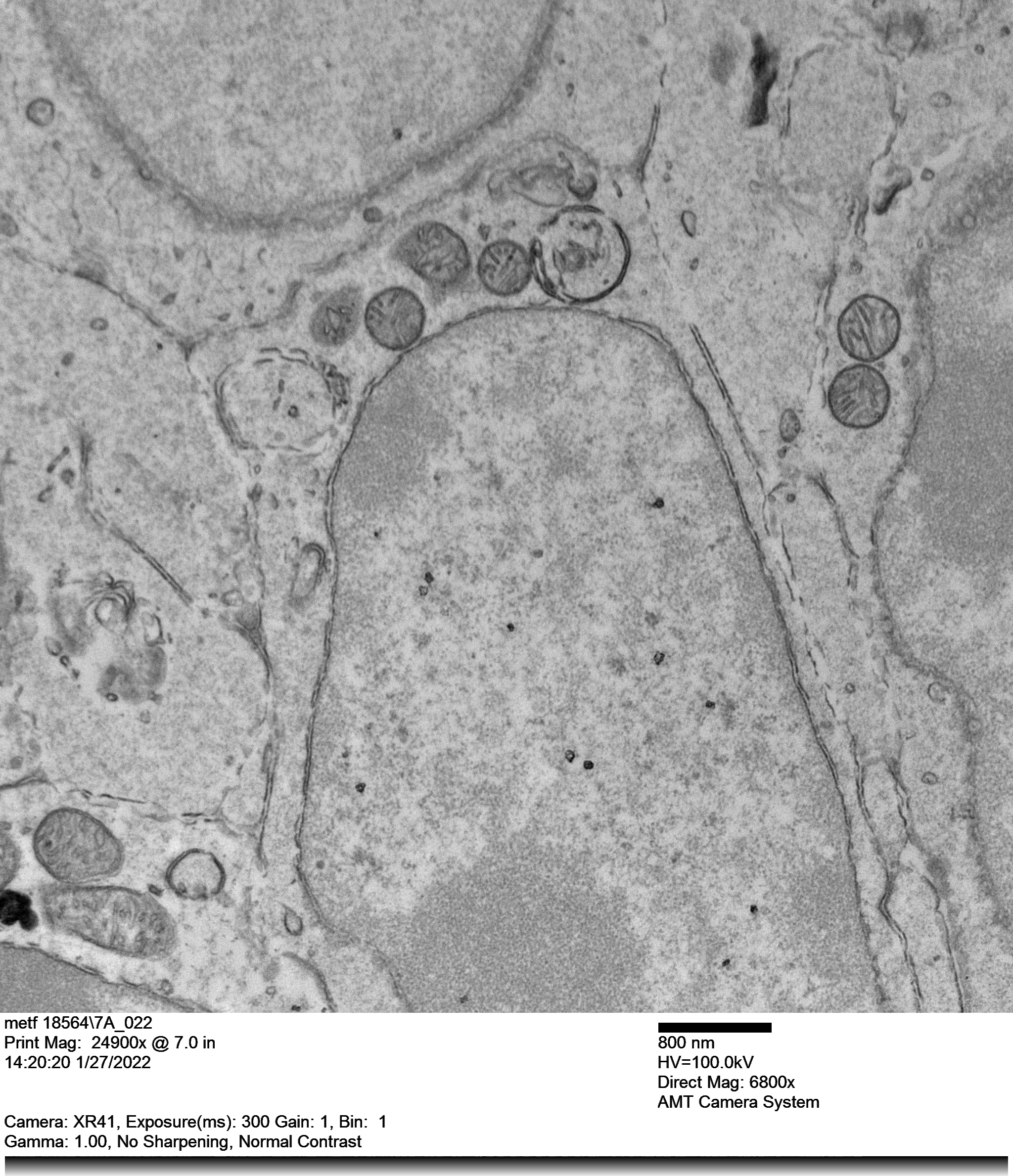

Supplement: Supplementary file 9 — Source Data for Figure 7 [file EMMM-15-e16908-s004.zip › Figure 7/7A/hNPC Control Metformin-example2.TIF]

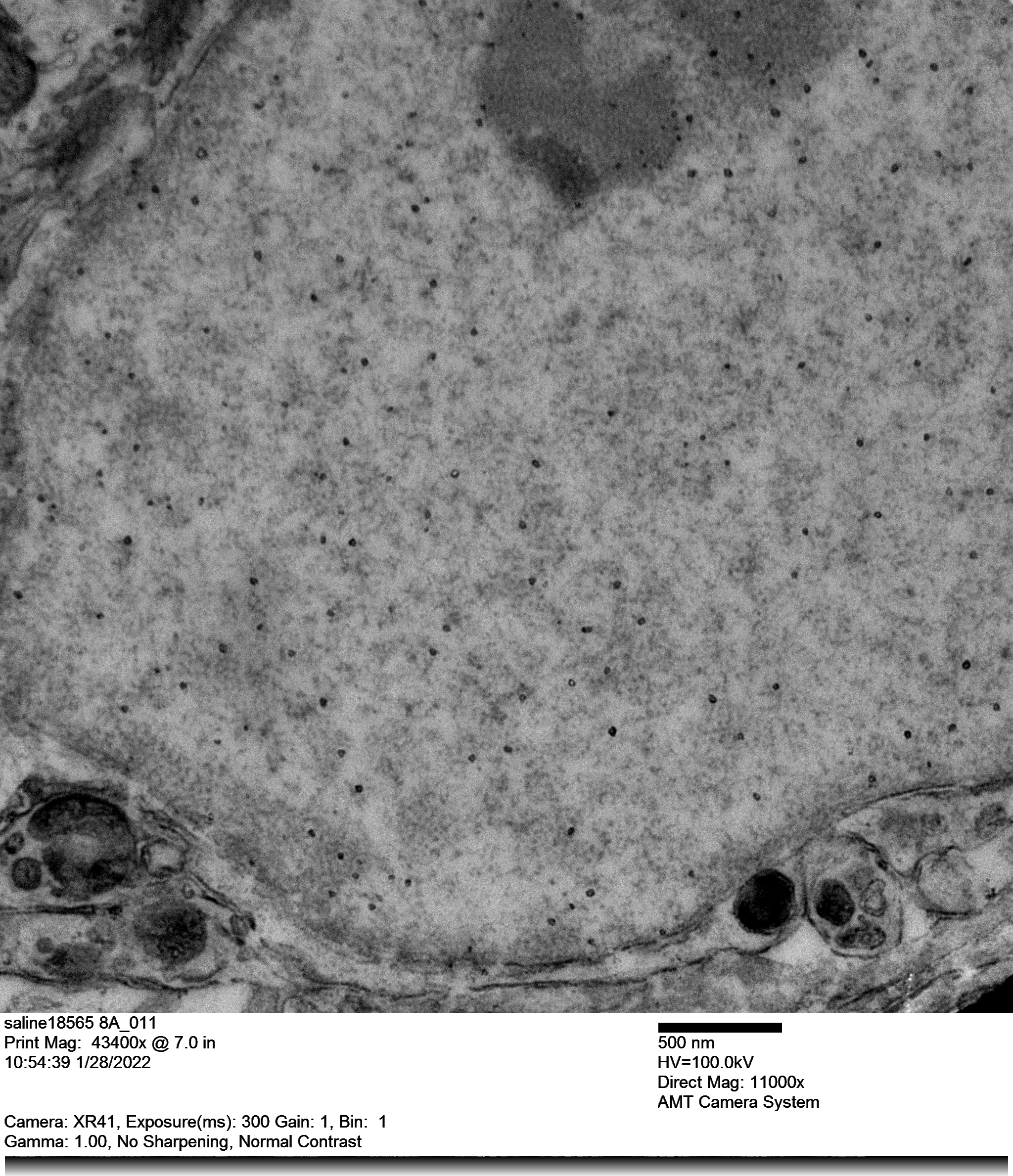

Supplement: Supplementary file 9 — Source Data for Figure 7 [file EMMM-15-e16908-s004.zip › Figure 7/7A/hNPC Control Saline - example1.TIF]

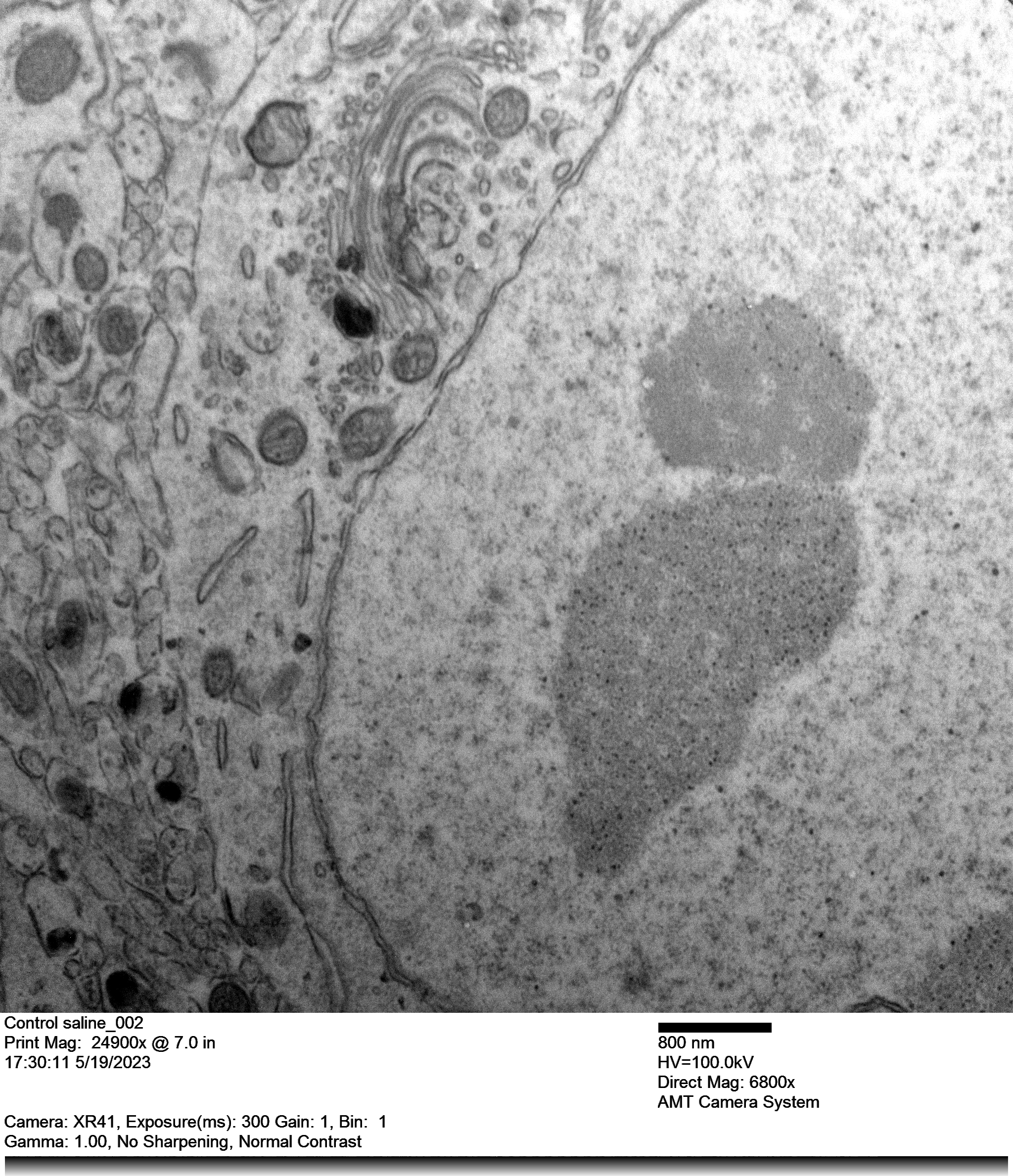

Supplement: Supplementary file 9 — Source Data for Figure 7 [file EMMM-15-e16908-s004.zip › Figure 7/7A/hNPC Control Saline - example2.TIF]

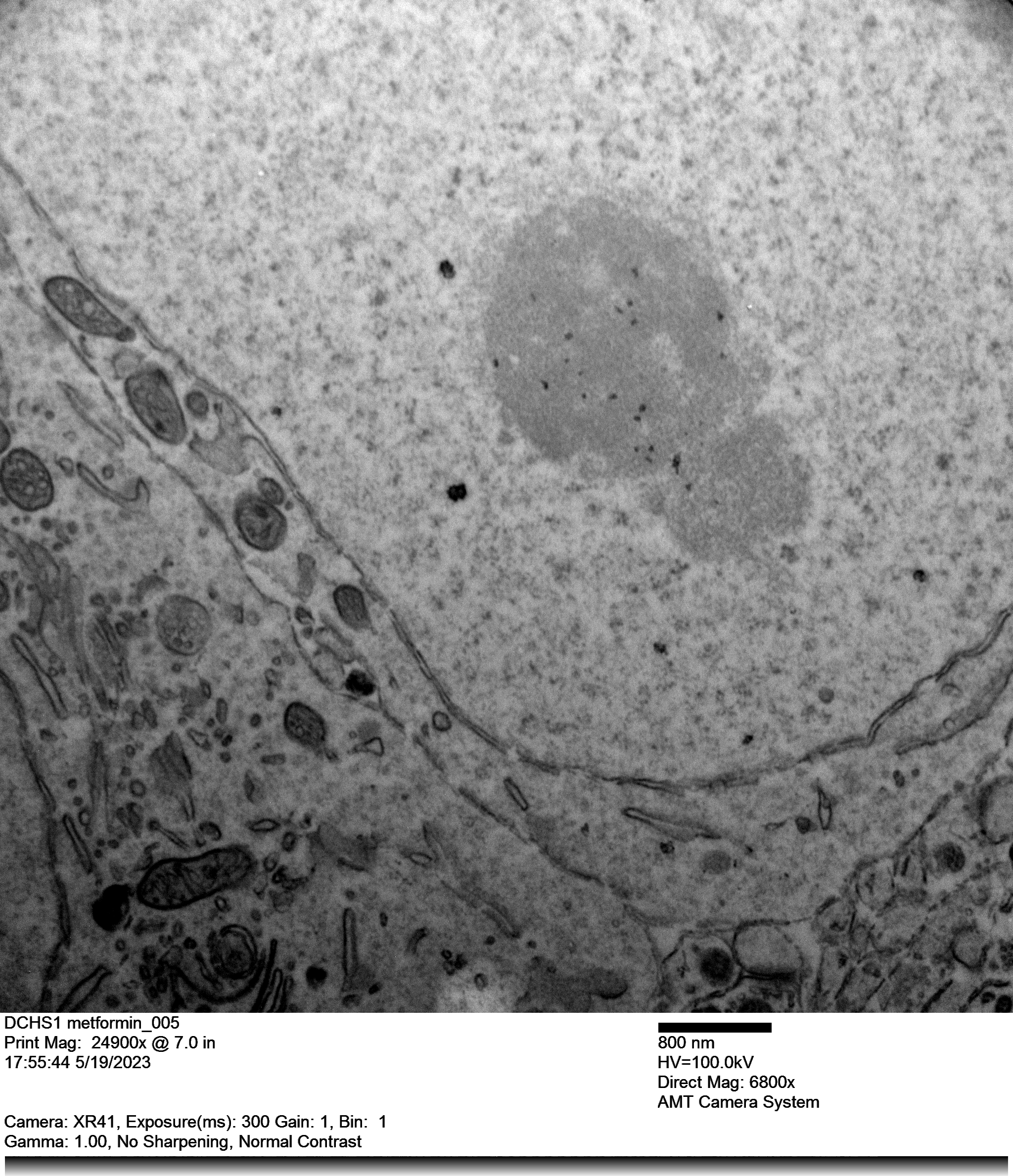

Supplement: Supplementary file 9 — Source Data for Figure 7 [file EMMM-15-e16908-s004.zip › Figure 7/7A/hNPC DCHS1 metformin - example1.TIF]

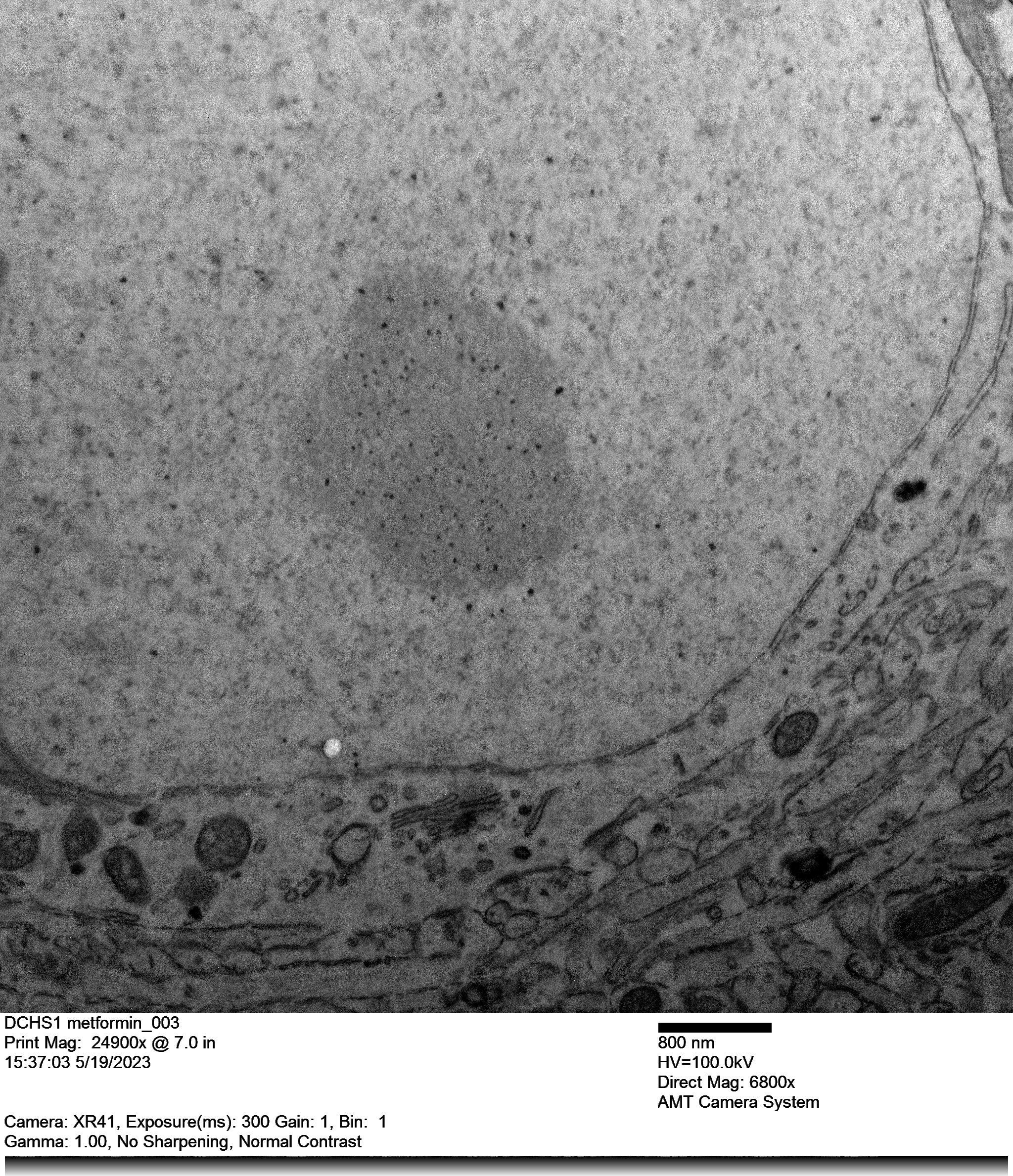

Supplement: Supplementary file 9 — Source Data for Figure 7 [file EMMM-15-e16908-s004.zip › Figure 7/7A/hNPC DCHS1 metformin - example2.TIF]

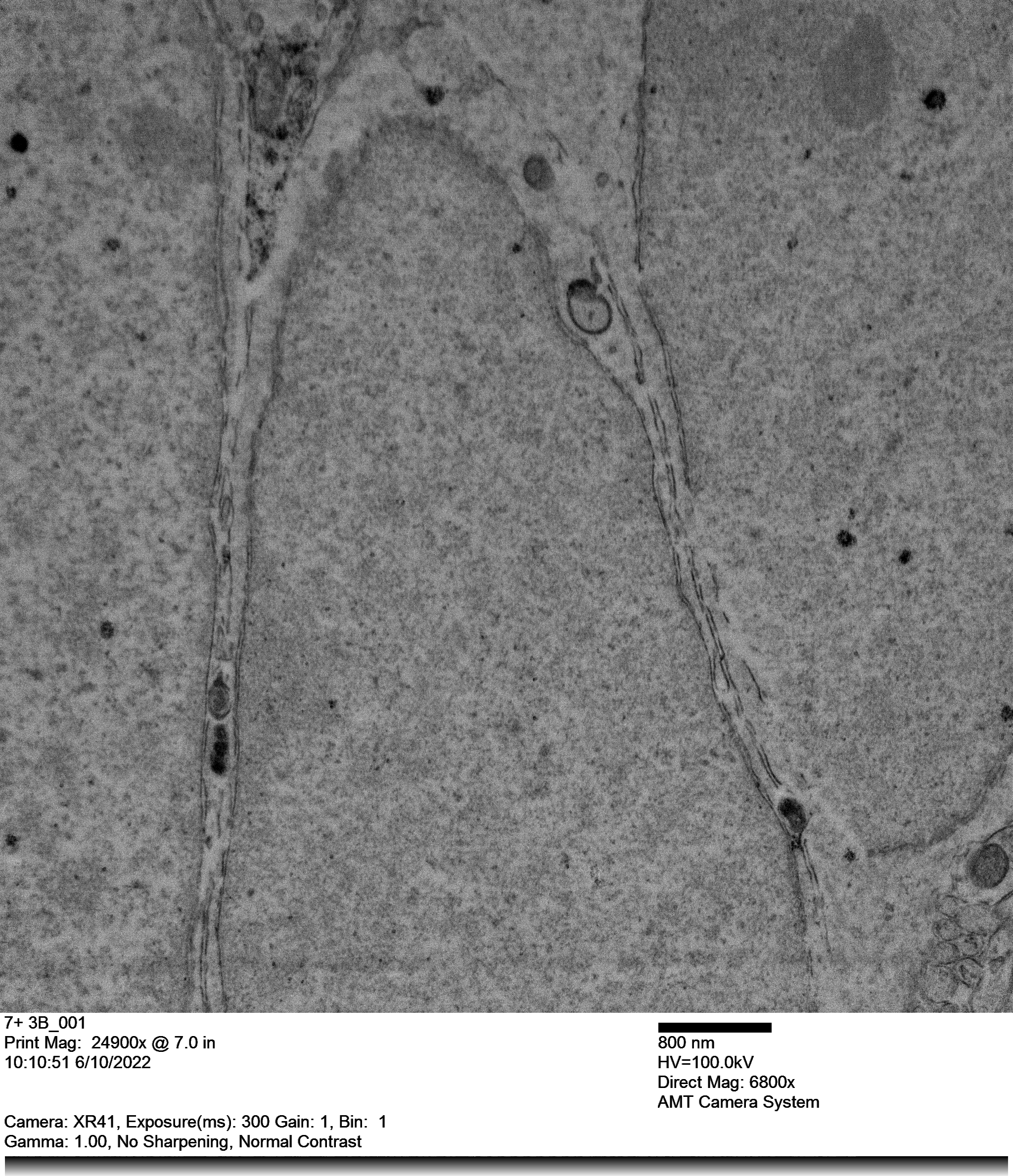

Supplement: Supplementary file 9 — Source Data for Figure 7 [file EMMM-15-e16908-s004.zip › Figure 7/7A/hNPC DCHS1 Saline - example1.TIF]

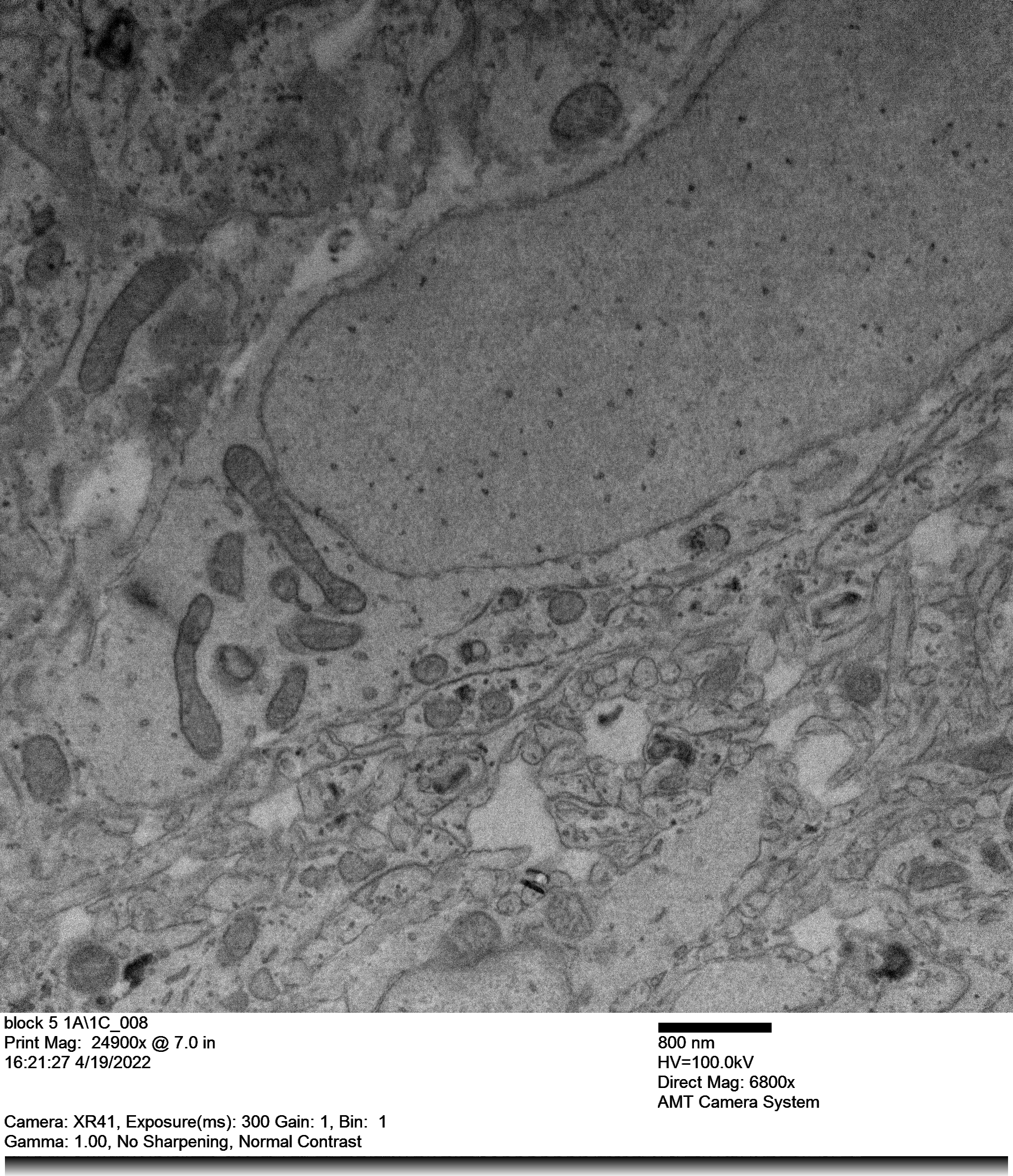

Supplement: Supplementary file 9 — Source Data for Figure 7 [file EMMM-15-e16908-s004.zip › Figure 7/7A/hNPC DCHS1 Saline-example2.TIF]

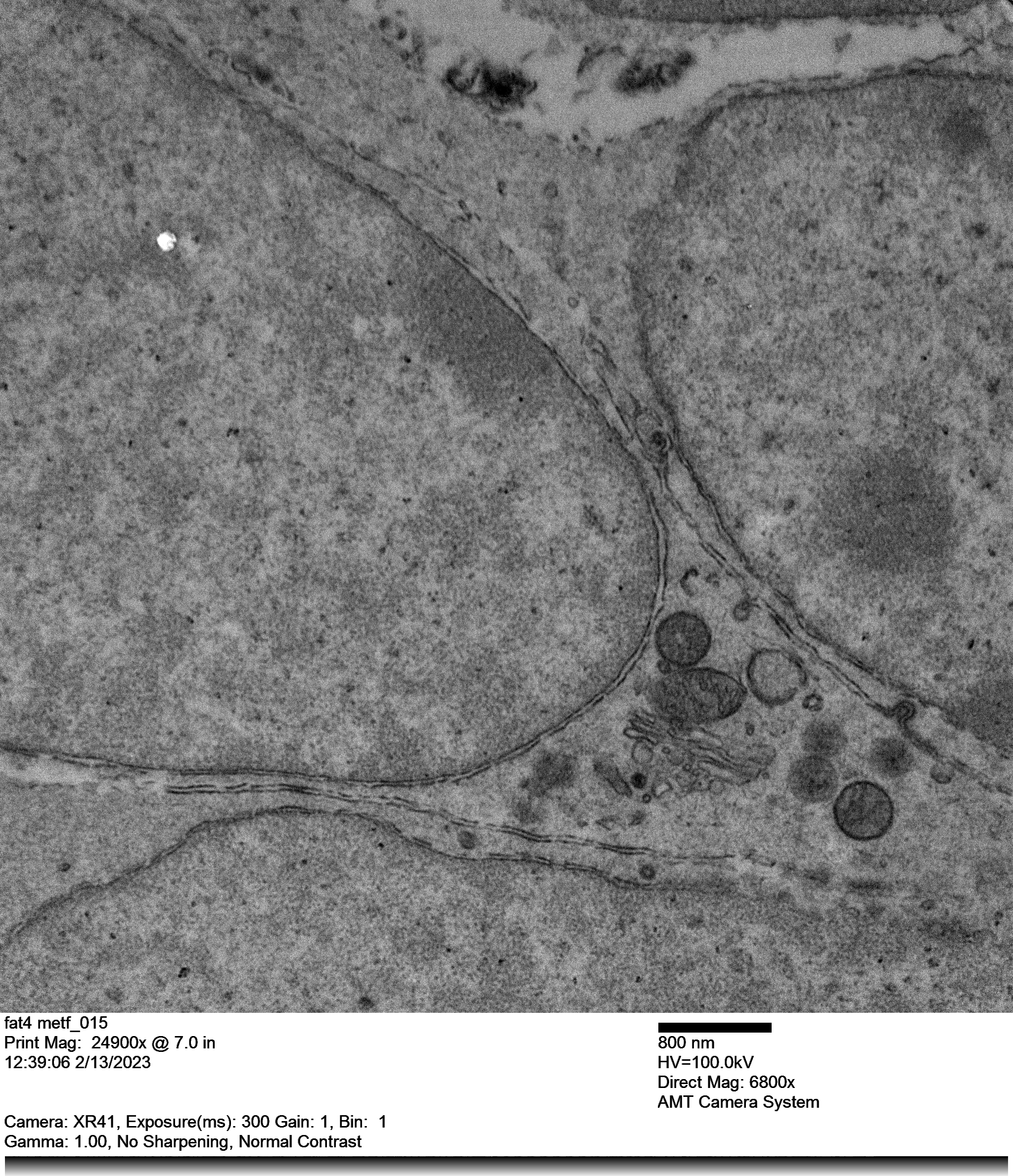

Supplement: Supplementary file 9 — Source Data for Figure 7 [file EMMM-15-e16908-s004.zip › Figure 7/7A/hNPC FAT4 Metformin - example1.TIF]

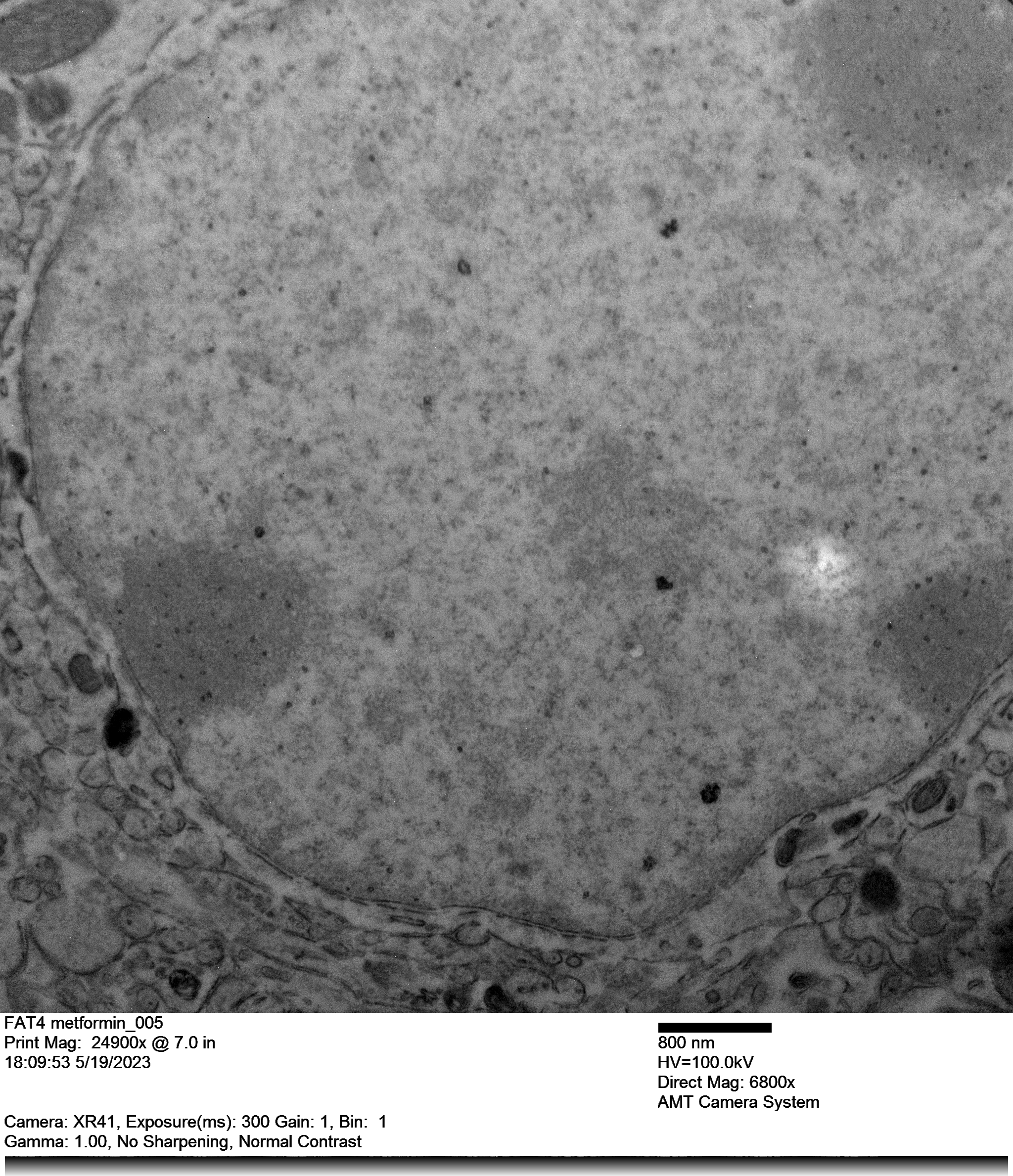

Supplement: Supplementary file 9 — Source Data for Figure 7 [file EMMM-15-e16908-s004.zip › Figure 7/7A/hNPC FAT4 Metformin-example2.TIF]

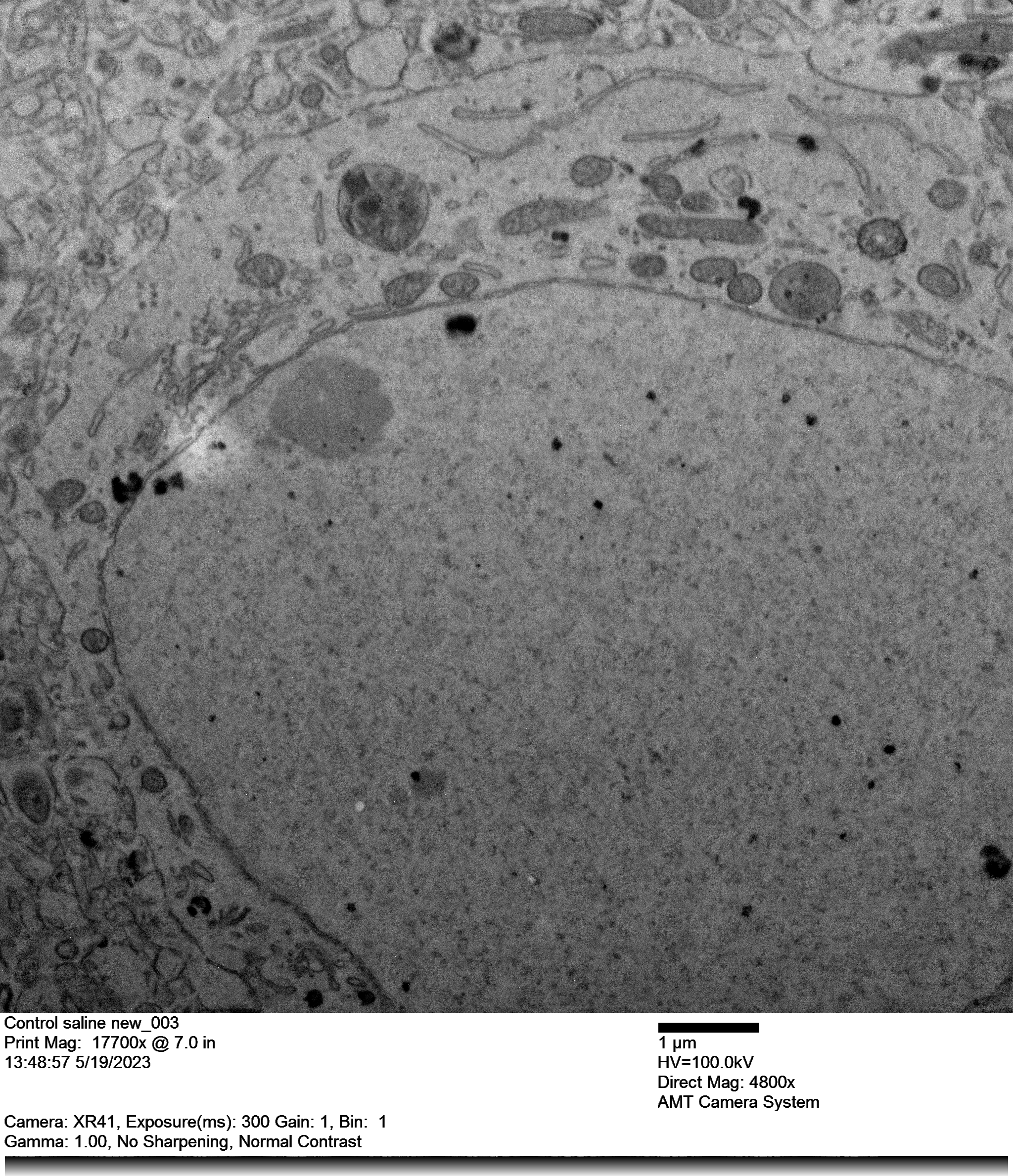

Supplement: Supplementary file 9 — Source Data for Figure 7 [file EMMM-15-e16908-s004.zip › Figure 7/7A/hNPC FAT4 Saline - example1.TIF]

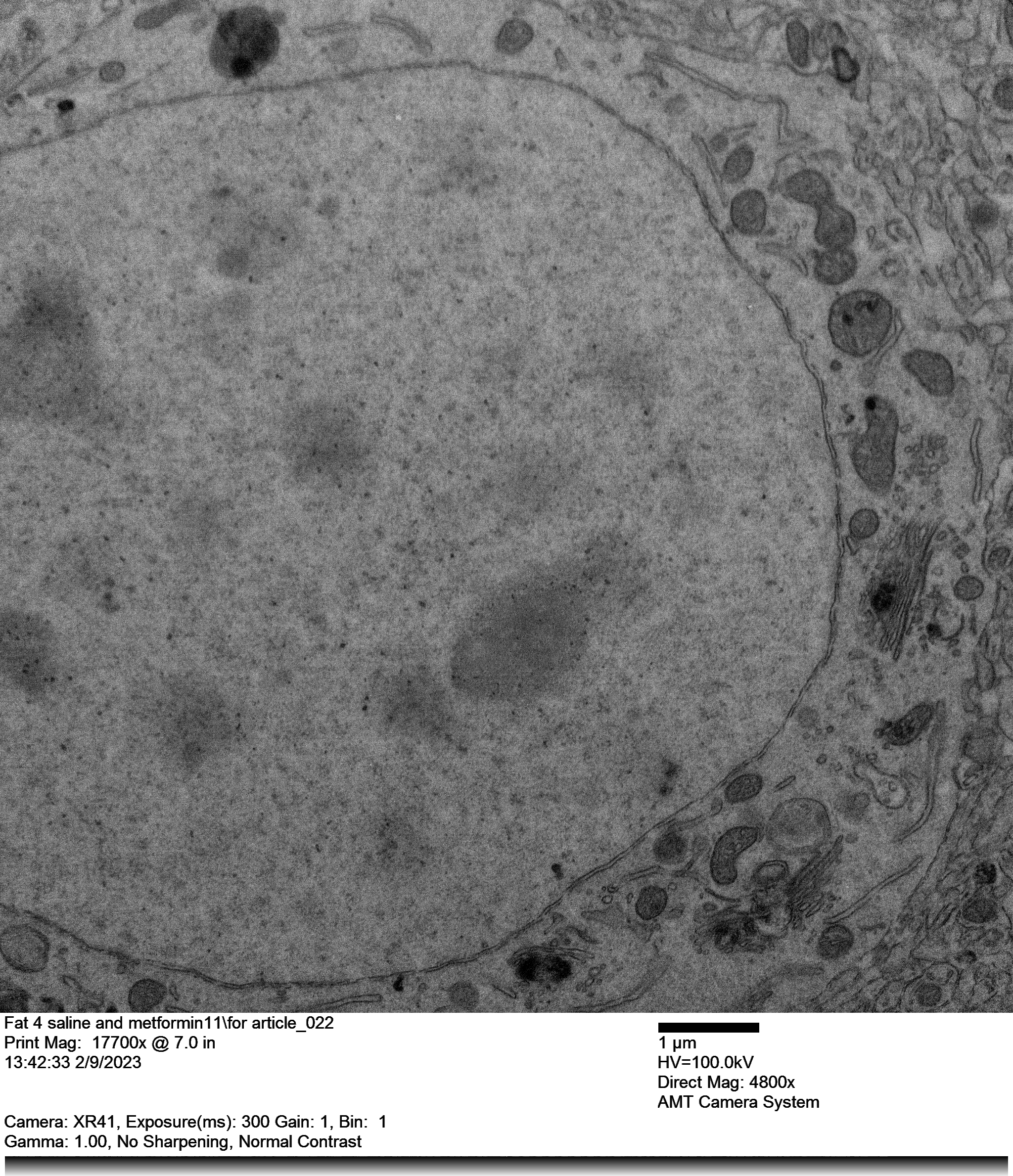

Supplement: Supplementary file 9 — Source Data for Figure 7 [file EMMM-15-e16908-s004.zip › Figure 7/7A/hNPC FAT4 Saline-example2.TIF]
