# Supplementary material for: Metformin rescues migratory deficits of cells derived from patients with periventricular heterotopia
Source: EMBO Mol Med. 2023 Aug 23;15(10):e16908. doi: 10.15252/emmm.202216908 (PMC10565636; doi:10.15252/emmm.202216908)
Supplement: Supplementary file 10 [file EMMM-15-e16908-s002.pdf]

EMBO Molecular Medicine

Metformin rescues migratory deficits of cells derived from patients with periventricular heterotopia

Cedric Bressan, Marta Snapyan, Marina Snapyan, Johannes Klaus, Francesco Di Matteo, Stephen Robertson, Barbara Treutlein, Martin Parent, Silvia Cappello, and Armen Saghatelyan
DOI: 10.15252/emmm.202216908

Corresponding author: Armen Saghatelyan (armen.saghatelyan@fmed.ulaval.ca)

Review Timeline:

Submission Date:	19th Sep 22
Editorial Decision:	13th Oct 22
Revision Received:	6th Jun 23
Editorial Decision:	7th Jul 23
Revision Received:	1st Aug 23
Accepted:	10th Aug 23

Editor: Zeljko Durdevic

Transaction Report:

(Note: With the exception of the correction of typographical or spelling errors that could be a source of ambiguity, letters and reports are not edited. Depending on transfer agreements, referee reports obtained elsewhere may or may not be included in this compilation. Referee reports are anonymous unless the Referee chooses to sign their reports.)

13th Oct 2022

Dear Dr. Saghatelyan,

Thank you for the submission of your manuscript to EMBO Molecular Medicine. We have now received feedback from the three reviewers who agreed to evaluate your manuscript. As you will see from the reports, all three referees recognize potential interest of the study but also raise seriuos and partially overlapping concerns, particularly regarding the biological replicates, limitations of cell lines derived from single patients and the lack of experiments using isogenic cell lines.

Further consideration of a revision that addresses reviewers' concerns in full will entail a second round of review. EMBO Molecular Medicine encourages a single round of revision only and therefore, acceptance or rejection of the manuscript will depend on the completeness of your responses included in the next, final version of the manuscript. For this reason, and to save you from any frustrations in the end, I would strongly advise against returning an incomplete revision.

We would welcome the submission of a revised version within six months for further consideration. Please let us know if you require longer to complete the revision.

I look forward to receiving your revised manuscript.

Yours sincerely,

Zeljko Durdevic

Zeljko Durdevic
Editor
EMBO Molecular Medicine

*****

When submitting your revised manuscript, please carefully review the instructions that follow below. We perform an initial quality control of all revised manuscripts before re-review; failure to include requested items will delay the evaluation of your revision.

We require:

1) A .docx formatted version of the manuscript text (including legends for main figures, EV figures and tables). Please make sure that the changes are highlighted to be clearly visible.

2) Individual production quality figure files as .eps, .tif, .jpg (one file per figure). For guidance, download the 'Figure Guide PDF': (https://www.embopress.org/page/journal/17574684/authorguide#figureformat).

3) A .docx formatted letter INCLUDING the reviewers' reports and your detailed point-by-point responses to their comments. As part of the EMBO Press transparent editorial process, the point-by-point response is part of the Review Process File (RPF), which will be published alongside your paper.

4) A complete author checklist, which you can download from our author guidelines (https://www.embopress.org/page/journal/17574684/authorguide#submissionofrevisions). Please insert information in the checklist that is also reflected in the manuscript. The completed author checklist will also be part of the RPF.

5) Please note that all corresponding authors are required to supply an ORCID ID for their name upon submission of a revised manuscript.

6) It is mandatory to include a 'Data Availability' section after the Materials and Methods. Before submitting your revision, primary datasets produced in this study need to be deposited in an appropriate public database, and the accession numbers and

database listed under 'Data Availability'. Please remember to provide a reviewer password if the datasets are not yet public (see https://www.embopress.org/page/journal/17574684/authorguide#dataavailability).

In case you have no data that requires deposition in a public database, please state so in this section. Note that the Data Availability Section is restricted to new primary data that are part of this study.

7) For data quantification: please specify the name of the statistical test used to generate error bars and P values, the number (n) of independent experiments (specify technical or biological replicates) underlying each data point and the test used to calculate p-values in each figure legend. The figure legends should contain a basic description of n, P and the test applied. Graphs must include a description of the bars and the error bars (s.d., s.e.m.). See also 'Figure Legend' guidelines: https://www.embopress.org/page/journal/17574684/authorguide#figureformat

8) At EMBO Press we ask authors to provide source data for the main and expanded view figures. Our source data coordinator will contact you to discuss which figure panels we would need source data for and will also provide you with helpful tips on how to upload and organize the files.

9) Our journal encourages inclusion of *data citations in the reference list* to directly cite datasets that were re-used and obtained from public databases. Data citations in the article text are distinct from normal bibliographical citations and should directly link to the database records from which the data can be accessed. In the main text, data citations are formatted as follows: "Data ref: Smith et al, 2001" or "Data ref: NCBI Sequence Read Archive PRJNA342805, 2017". In the Reference list, data citations must be labeled with "[DATASET]". A data reference must provide the database name, accession number/identifiers and a resolvable link to the landing page from which the data can be accessed at the end of the reference. Further instructions are available at .

10) We replaced Supplementary Information with Expanded View (EV) Figures and Tables that are collapsible/expandable online. A maximum of 5 EV Figures can be typeset. EV Figures should be cited as 'Figure EV1, Figure EV2" etc... in the text and their respective legends should be included in the main text after the legends of regular figures.

- For the figures that you do NOT wish to display as Expanded View figures, they should be bundled together with their legends in a single PDF file called *Appendix*, which should start with a short Table of Content. Appendix figures should be referred to in the main text as: "Appendix Figure S1, Appendix Figure S2" etc.

- Additional Tables/Datasets should be labeled and referred to as Table EV1, Dataset EV1, etc. Legends have to be provided in a separate tab in case of .xls files. Alternatively, the legend can be supplied as a separate text file (README) and zipped together with the Table/Dataset file.

See detailed instructions here:

.

11) The paper explained: EMBO Molecular Medicine articles are accompanied by a summary of the articles to emphasize the major findings in the paper and their medical implications for the non-specialist reader. Please provide a draft summary of your article highlighting

- the medical issue you are addressing,

- the results obtained and

- their clinical impact.

This may be edited to ensure that readers understand the significance and context of the research. Please refer to any of our published articles for an example.

12) For more information: There is space at the end of each article to list relevant web links for further consultation by our readers. Could you identify some relevant ones and provide such information as well? Some examples are patient associations, relevant databases, OMIM/proteins/genes links, author's websites, etc...

13) Author contributions: You will be asked to provide CRediT (Contributor Role Taxonomy) terms in the submission system. These replace a narrative author contribution section in the manuscript.

14) A Conflict of Interest statement should be provided in the main text.

15) Every published paper now includes a 'Synopsis' to further enhance discoverability. Synopses are displayed on the journal

webpage and are freely accessible to all readers. They include a short stand first (maximum of 300 characters, including space) as well as 2-5 one-sentences bullet points that summarizes the paper. Please write the bullet points to summarize the key NEW findings. They should be designed to be complementary to the abstract - i.e. not repeat the same text. We encourage inclusion of key acronyms and quantitative information (maximum of 30 words / bullet point). Please use the passive voice. Please attach these in a separate file or send them by email, we will incorporate them accordingly.

Please also suggest a striking image or visual abstract to illustrate your article as a PNG file 550 px wide x 300-600 px high.

EMBO Molecular Medicine has a "scooping protection" policy, whereby similar findings that are published by others during review or revision are not a criterion for rejection. Should you decide to submit a revised version, I do ask that you get in touch after three months if you have not completed it, to update us on the status.

Please note: When submitting your revision you will be prompted to enter your funding and payment information. This will allow Wiley to send you a quote for the article processing charge (APC) in case of acceptance. This quote takes into account any reduction or fee waivers that you may be eligible for. Authors do not need to pay any fees before their manuscript is accepted and transferred to the publisher.

EMBO Press participates in many Publish and Read agreements that allow authors to publish Open Access with reduced/no publication charges. Check your eligibility: https://authorservices.wiley.com/author-resources/Journal-Authors/open-access/affiliation-policies-payments/index.html

***** Reviewer's comments *****

Referee #1 (Comments on Novelty/Model System for Author):

This paper reports some interesting observations that indicate an involvement of disruption of the autophagy flux in neural migration disorders caused by mutations in two genes, DCHS1 and FAT4. While the presented work is of interest, I note several weaknesses that dampen my enthusiasm for this work.
- All experiments are based on hNPCs derived from 1 control, 1 patient with a homozygous DCHS1 mutation and 1 patient with compound heterozygous FAT4 mutations. It is not clear at all whether these single lines are representative for the PH disorders they represent. A multiple patient-derived lines as well as isogenic lines in which mutations were introduced (in a control) or rescued (in a patient line).
- Some experiments were done only for the DCHS1 line, not FAT4. In particular, the metformin experiment was conducted only for the DCHS1 and control hNPCs, which makes the suggestion that metformin has rescue effects in PH patients quite weak. In addition, how do the authors envisage a beneficial effect for metformin in a neurodevelopmental disorder with neural migration defects occurring in fetal development and numerous other clinical features?
- There is little data showing a mechanism underlying the autophagy defects. The authors exclude transcription deregulation, showing no significant consistent deregulation of autophagy-related genes. This is a negative exclusion. Have they looked at other genes than those listed in figure 2D?
- Some inconsistencies are noted in migration characteristics across the various figures (see notes to authors).

My low score for adequacy of the model system is related to my criticism on the first two bullet points. Extensive revisions would be required to consider publication, but at least these points need to be dealt with by the authors.

Referee #1 (Remarks for Author):

The novelty of this paper is that autophagy-linked defects are associated to mutations in two genes that have been linked periventricular heterotopia. This is a first in vivo demonstration that disrupted autophagy underly two related genetic neuronal migration disorders. To demonstrate this, the authors have use human neuronal progenitor cells xenografted in immunodeficient mice. Migration defects could be partially restored by modulating the autophagic flux, which may suggest a potential strategy for intervention. These results are exciting, however, there are several issues that need to be addressed:
- All experiments are based on hNPCs derived from 1 control, 1 patient with a homozygous DCHS1 mutation and 1 patient with compound heterozygous FAT4 mutations. It is not clear at all whether these single lines are representative for the PH disorders they represent. A multiple patient-derived lines as well as isogenic lines in which mutations were introduced (in a control) or rescued (in a patient line).
- Some experiments were done only for the DCHS1 line, not FAT4. In particular, the metformin experiment was conducted only for the DCHS1 and control hNPCs, which makes the suggestion that metformin has rescue effects in PH patients quite weak. In addition, how do the authors envisage a beneficial effect for metformin in a neurodevelopmental disorder with neural migration defects occurring in fetal development and numerous other clinical features?
- There is little data showing a mechanism underlying the autophagy defects. The authors exclude transcription deregulation, showing no significant consistent deregulation of a selection of autophagy-related genes (fig 2D). This is a negative exclusion. Is there deregulation of other genes, whether involved in autophagy flux or in other cellular processes, that may affect migration?
- Some inconsistencies are noted in migration characteristics across the various figures. For example, Based on fig 1D it is

concluded that there is no change in the speed of migration in DCHS1 and FAT4 hNPCs. However, in fig 3F there seems to be a clear reduction of speed for FAT4, while migration of DCHS1 is normal. This difference needs discussion.
- As expected, Bafilomycin has a clear effect on distance, speed and migratory phases of control hNPC grafts. The effects for DCHS1 and FAT4 seem to be more ambiguous. E.g. the speed of migration is reduced for DCHS1 and not for FAT4. How can this be explained? Moreover, the reductions of all parameters for control hNPCs reach levels that are significantly lower as the levels for treated and non-treated DCHS1 and FAT4. Even if fusion of autophagosomes and lysosomes is already reduced in mutant neurons, one wonders why bafilomycin does not more completely abolish migration parameters as for control cells. What are the reasons for this? This observation suggests other mechanisms in addition and perhaps more important than autophagy affecting migration.
- Autophagosome formation seems to be intact and even occupy a larger area in DCHS1/FAT4 hNPCs, suggesting a defective fusion with lysosomes. Lysosome area and size seems to be unaltered in these organoids (fig 3B/C), even in DCHS1 hNPCs treated with metformin, which stimulates fusion with lysomes and which reduces the autophagosome area in these hNPCs. Would one not expect to see any difference in lysosome area?
There are various typographical errors in the text and figure legends that need correction. Nomenclature of gene/protein names is inconsistent, especially in the Discussion section, and needs to be checked throughout.

Referee #2 (Remarks for Author):

The work of Bressan et al. investigates the mechanisms underlying the migration defects in patients with cortical malformation due to mutations in DCHS and FAT4. Using patient-derived organoids and grafting human neural progenitor cells (hNPCs) into the mouse brain, the authors focus on the in vivo context linking affected migration to autophagy defects. While the overall concept is interesting and well thought-through, the performed experiments are less convincing and lack clarity. For publication, the authors need to address to following points:

1. A major issue of this work are the biological replicates. While the authors always state how many cells have been analyzed, I did not find information on how many animals have been imaged. For example, for migration analysis, 21 control cells have been analyzed (Figure 1D). Do these 21 cells all come from one xenotransplanted animal? Then the biological replicates are too low with n=1. If 21 cells have been analyzed in different animals, the number of cells per animals is too low. And why is the cell number so different between experimental conditions (n=21 ctrl; n=47 for DCHS; n=17 FAT4)? The same applies for the time-lapse imaging of autophagy (Figure 3D, F).
2. Information on the control line is lacking. Here, the gold standard is isogene control via CRISPR correction. Does this apply to this control line?
3. The authors write that they performed single cell RNA sequencing (scRNA seq) of control, DCHS and FAT4 organoids, but I did not find any information on that in their material and methods. My guess is that they reanalyzed an existing data set (Klaus et al., 2019), which is totally fine, but should be clearly stated.
4. Figure 2a: Is the LC3-staining reliable? In the images shown, LC3 looks rather cytoplasmic. Here, high resolution images showing autophagosomal structures would be appreciated. Furthermore, why do the authors only show LC3-staining in control organoids? Given their results on the western blot, it would be particularly interesting to see this staining in DCHS and FAT4-derived organoids, and to also directly combine it with Paxillin immune staining. In addition, co-stainings and western blot analyses for lysosomal markers would also be interesting to further assess autophago-lysosomal degradation in control and mutant organoids as the authors could not detect differences in the autophago-lysosomal pathway on transcriptional level.
5. The authors report an increase in Paxillin and the lipidated form of LC3 in Western blot from DCHS and FAT4 mutant organoids. This increase is rather small than very large as stated in the text, but it is significant. An increase in LC3 can mean either more autophagy or impaired autophagy. Here, p62 expression on the same western blot would directly tell which one is the case as p62 would accumulate in case of autophagic defects. Furthermore, classical autophagy turnover assays using BafilomycinA1/Chloroquin are missing.
6. The EM pictures are nice, but it is impossible to judge how stabile the phenotype is. It is again necessary to report the biological replicates (as mentioned before). Maybe more images from different biological replicates in the Supplemental Material would help, too.
7. The BafilomycinA1 concentration used in the migration studies appears very high. Since Bafilomycin is toxic to the cells, I am concerned about cell death effects. Have the authors performed a dose response curve?
8. The in vivo rescue with Metformin is very interesting. However, also here, overall biochemical assays, such as Western blot analyses for LC3 and p62, lysosomal markers, autophagy turnover assays using BafilomycinA1/Chloroquin as well as immunostainings against LC3 and Paxillin would be needed to first verify that Metformin is actual rescuing the suggested autophagic defect by decreasing Paxillin levels. This would significantly strengthen their hypothesis that the accumulation of Paxillin due to impairments in autophagy dependent degradation might lead to the observed migration deficit.

Referee #3 (Remarks for Author):

Dear editor find attached my comments on manuscript EMM-2022-16908, entitled " Periventricular heterotopia induced by DCHS1 and FAT4 mutations is linked to autophagy-dependent alterations in neuronal migration and can be rescued by metformin" by Bressan et al.

Overal I found the paper very interesting, highly relevant, and technically very challenging, which the authors should be commended on their hard work. , and I think its worthy of publication in your journal. However, there are a few issues I would like the authors to address, before this can happen.

Figure 1, Legend section D , sentence starting " n=40 cells
for endogenous neuroblasts, n=21 cells for control hNPCs, n=47 cells for hNPCs with a mutation
in DCHS1, n=17 cells for hNPCs with a mutation in.* p < 0.05, ** p < 0.005, *** p < 0.001 with
a one-way ANOVA followed by a post hoc LSD Fisher." FAT4 is missing!

Figure 2 A-not sure if immunos are really showing or telling us very much here, as these are only performed on control organoids. Also they need to clarify what DCX is staining for, whether its migrating interneurons, or other types of cells?, as this is not clearly stated in text.
Figure 2B The author stress the increase in levels of LC3-II in the western blots, but there is also clearly an increase in LC3-I , which is not nemtioned? Also how do the levels compare against each other, so if authors could calulate the ratios between the 2, it would be interesting to see if this changes or stays the same!
It would also be great if the authors could also confirm their western blot findings by either show immunohstochemisrty or QPCR for LC3-I and LC3-II , and paxillin in control.FAT4 and DCHs1 cultures.

Figure 3. To be honest I found it very difficult to see what is going on in the EM pictures, and to see any obvious differences between the images shown. The only comment I can make is that there are no lysosomes highlighted inDCHs1 or FAT4 cultures, is this true, also same comment for 4D? So it would be better for the authors to remove white arrows pointing to gfp gold labelling and add more arrow heads pointing out autophagosomes and lysosomes, so the reader can make an informed decision about what is being shown. This also applies to EM in figure 4

Reviewers' Comments:

Reviewer #1: (Comments on Novelty/Model System for Author):

This paper reports some interesting observations that indicate an involvement of disruption of the autophagy flux in neural migration disorders caused by mutations in two genes, DCHS1 and FAT4. While the presented work is of interest, I note several weaknesses that dampen my enthusiasm for this work.

We thank the reviewer for his/her comments. We have revised the manuscript and have included the results of additional *in vivo* and *ex vivo* experiments and analyses to strengthen our observations.

- All experiments are based on hNPCs derived from 1 control, 1 patient with a homozygous DCHS1 mutation and 1 patient with compound heterozygous FAT4 mutations. It is not clear at all whether these single lines are representative for the PH disorders they represent. A multiple patient-derived lines as well as isogenic lines in which mutations were introduced (in a control) or rescued (in a patient line).

We have added several additional hNPCs lines to strengthen our observations. First, the results from the two additional lines from unaffected subjects (Figure 1D, hNPCs Control 1 and Figure 1E) showed that the migratory parameters of hNPCs derived from these lines are similar to those of the initial control line (Figure 1D, hNPCs Control 2) and are significantly different from those of the patient hNPCs. Second, we performed time-lapse imaging of isogenic lines with a DCHS1 or FAT4 knockdown that were xenotransplanted into the brains of immunodeficient mice. Our new data confirmed the initial analysis and showed that the migratory dynamics of hNPCs derived from the patients and the isogenic lines with either a DCHS1 or a FAT4 knockdown are affected compared to the three control lines. We also attempted to perform time-lapse imaging of isogenic knock-out lines that were generated previously (Klaus et al., 2019). Unfortunately, however, there was a major contamination in all these cultures and we had to rederive all the iPSC lines. This took us several months, and the accomplishment of these experiments was further delayed by the relocation of the two main labs involved in this project (Silvia Cappello

and Armen Saghatelyan) to Ludwig Maximilian University of Munich and University of Ottawa, respectively. Given the time required to set up the cell culture facility, perform the injections in immunodeficient mice, and carry out the time-lapse imaging of the brain slices we were unable to perform these experiments within the extended timeframe of revisions. We think, however, that our new data with isogenic knock-down lines (**Figure 1E**) as well as additional control lines support our previous observations and address the reviewer's concern.

The reviewer also suggested using multiple patient-derived lines. To the best of our knowledge, there are no additional iPSC lines with DCHS1 or FAT4 mutations. The other lines derived from PH patients harbor mutations in different genes. For example, mutations in filamin 1 (FLN1), endothelin-converting enzyme-2 (ECE2), and secreted LGALS3BP have all been linked to distinct forms of PH (Buchsbaum and Cappello, 2019; Buchsbaum et al., 2020; Kyrousi et al., 2021). The underlying mechanisms and the role of autophagy in these patient-derived lines would, however, have to be explored in follow-up studies, which were not possible within the revision timeline. We now state in our manuscript that the role of autophagy in other forms of PH needs to be explored further and emphasize that we have investigated a form of PH characterized by DCHS1 and FAT4 mutations.

- Some experiments were done only for the DCHS1 line, not FAT4. In particular, the metformin experiment was conducted only for the DCHS1 and control hNPCs, which makes the suggestion that metformin has rescue effects in PH patients quite weak. In addition, how do the authors envisage a beneficial effect for metformin in a neurodevelopmental disorder with neural migration defects occurring in fetal development and numerous other clinical features?

To address the reviewer's comment, we performed new experiments to test the role of metformin in rescuing migratory deficits of FAT4-derived hNPCs. We performed both time-lapse imaging of xenotransplanted hNPCs derived from a patient with a FAT4 mutation and an EM analysis of autophagosomes and lysosomes of grafted cells. Our new experiments and analysis (shown in **Figure 4**) revealed that i.p. injections of metformin also rescue the migratory abnormalities of hNPCs with the FAT4 mutation and concomitantly correct the autophagosomal area. These data reinforce our observations that metformin may be a promising avenue for treating migratory deficits of PH characterized by a DCHS1 or FAT4 mutation.

We thank the reviewer for the interesting question regarding a possible beneficial effect of metformin in neurodevelopmental disorders that occur during fetal development. We would like to address this issue in follow-up studies using both animal models and clinical data. First, we will test the role of metformin in mouse models with DCHS1 and FAT4 mutations that have been previously characterized (Cappello et al., 2013). Second, we aim to develop a xenotransplantation approach for use in the embryonic ventricular zone and inject metformin into pregnant females to assess its effect on hNPCs during fetal development. This will also allow us to characterize the time window and the concentration and number of metformin injections required to rescue, at least partially, migratory defects during fetal development. Third, we would like to point out that metformin is already in clinical use for the treatment of pregnancy-associated diabetes (Hyer et al., 2018; Benham et al., 2021; Buchanan et al., 2012; Newman and Dunne, 2022). We will, in collaboration with clinicians, collect and interrogate clinical data sets of PH patients to determine whether there are any cases where metformin has been given to mothers of PH patients during their pregnancy and, if so, assess the propensity and severity of the PH phenotype and other clinical features of the children. The time windows for the metformin treatment and the associated clinical features will also be evaluated.

- There is little data showing a mechanism underlying the autophagy defects. The authors exclude transcription deregulation, showing no significant consistent deregulation of autophagy-related genes. This is a negative exclusion. Have they looked at other genes than those listed in figure 2D?

We examined 22 autophagy and 16 lysosomal biogenesis/function-related genes. The expression of all these genes was largely unaffected. We thus concluded that the regulation of autophagy is transcription-independent. Several non-autophagy-related genes with affected expression have been reported in a previous publication using the same RNAseq dataset (Klaus et al., 2019). For example, the *ROBO3, NDNF, DCC, CNTN2, EPHB3, EFNA3,* and *ITGB1* genes are all involved in cell migration, and their dysregulation has been observed in organoids derived from hNPCs with DCHS1 and FAT4 mutations (Klaus et al., 2019). While all these genes may contribute to the migratory deficits observed in hNPCs with DCHS1 and FAT4 mutations, the aim of our study was to determine whether it was possible to rescue the migratory deficits of hNPCs with DCHS1

and FAT4 mutations by modulating migratory pathways not transcriptionally affected by PH-inducing genetic mutations. Since neuronal migration depends on multiple molecular pathways, we believe that this approach provides a valuable strategy for correcting the migratory defects that occur during fetal development.

We have previously shown that autophagy is homeostatically modulated in response to various migration-promoting and migration-inhibiting cues to cope with the pace and periodicity of neuronal migration (Bressan et al., 2020). In the present study, we focused on the role of autophagy in DCHS1 and FAT4 mutation-induced PH. Our Western blotting in organoids, immunohistochemistry for autophagy and lysosomal proteins in grafted cells (new data in the revised manuscript), time-lapse imaging with and without Bafilomycin A1, and EM analyses of grafted cells with and without the metformin treatment, all suggest that the autophagy process is affected. In line with our previous data from mouse neuroblasts (Bressan et al., 2020), we propose that a decrease in autophagic flux in hNPCs is a homeostatic adaptation in cells with affected neuronal migration. In other words, a low pace of migratory cells with DCHS1 and FAT4 mutations would require a lower autophagic flux for recycling focal adhesion molecules. However, as the expression of autophagy-related genes is unaffected, bolstering the autophagy process fosters neuronal migration by triggering recycling of focal adhesions, as our experiments with metformin suggest. We have revised the Discussion to reflect these points.

- Some inconsistencies are noted in migration characteristics across the various figures (see notes to authors).

Please see our response below.

My low score for adequacy of the model system is related to my criticism on the first two bullet points. Extensive revisions would be required to consider publication, but at least these points need to be dealt with by the authors.

We have performed new experiments and have made extensive revisions to address the reviewer's concerns.

Reviewer #1 (Remarks for Author):

The novelty of this paper is that autophagy-linked defects are associated to mutations in two genes that have been linked periventricular heterotopia. This is a first in vivo demonstration that disrupted autophagy underly two related genetic neuronal migration disorders. To demonstrate this, the authors have use human neuronal progenitor cells xenografted in immunodeficient mice. Migration defects could be partially restored by modulating the autophagic flux, which may suggest a potential strategy for intervention. These results are exciting, however, there are several issues that need to be addressed:

We thank the reviewer for his/her positive comments and suggestions. Based on the reviewer's comments we performed additional experiments and analyses to strengthen our manuscript.

- All experiments are based on hNPCs derived from 1 control, 1 patient with a homozygous DCHS1 mutation and 1 patient with compound heterozygous FAT4 mutations. It is not clear at all whether these single lines are representative for the PH disorders they represent. A multiple patient-derived lines as well as isogenic lines in which mutations were introduced (in a control) or rescued (in a patient line).

- Some experiments were done only for the DCHS1 line, not FAT4. In particular, the metformin experiment was conducted only for the DCHS1 and control hNPCs, which makes the suggestion that metformin has rescue effects in PH patients quite weak. In addition, how do the authors envisage a beneficial effect for metformin in a neurodevelopmental disorder with neural migration defects occurring in fetal development and numerous other clinical features?

- There is little data showing a mechanism underlying the autophagy defects. The authors exclude transcription deregulation, showing no significant consistent deregulation of a selection of autophagy-related genes (fig 2D). This is a negative exclusion. Is there deregulation of other genes, whether involved in autophagy flux or in other cellular processes, that may affect migration?

Please see our responses above. We have addressed all these points in the section "*Comments on Novelty/Model System for Author.*"

- Some inconsistencies are noted in migration characteristics across the various figures. For example, Based on fig 1D it is concluded that there is no change in the speed of migration in DCHS1 and FAT4 hNPCs. However, in fig 3F there seems to be a clear reduction of speed for FAT4, while migration of DCHS1 is normal. This difference needs discussion.

Indeed, the speed of migration of FAT4 hNPCs is lower in Figure 3. This difference is largely due to the presence of two cells that did not migrate (showing 0 values) under baseline conditions and migrated slightly after the addition of Bafilomycin A1. We included all cells in our analysis. As such, the inclusion of these two cells decreased the average values for the speed of migration of FAT4 hNPCs. We also performed new experiments with FAT4 hNPCs following i.p. injections of metformin and did not observe any changes in the speed of migration. Furthermore, our new experiments with FAT4 miRNA support our observations that there is no difference in the speed of migration.

- As expected, Bafilomycin has a clear effect on distance, speed and migratory phases of control hNPC grafts. The effects for DCHS1 and FAT4 seem to be more ambiguous. E.g. the speed of migration is reduced for DCHS1 and not for FAT4. How can this be explained? Moreover, the reductions of all parameters for control hNPCs reach levels that are significantly lower as the levels for treated and non-treated DCHS1 and FAT4. Even if fusion of autophagosomes and lysosomes is already reduced in mutant neurons, one wonders why bafilomycin does not more completely abolish migration parameters as for control cells. What are the reasons for this? This observation suggests other mechanisms in addition and perhaps more important than autophagy affecting migration.

We think that since the autophagy process is stalled in DCHS1 and FAT4 hNPCs due to the fact that the fusion of autophagosomes with lysosomes is affected, Bafilomycin A1 has a stronger effect in control hNPCs that display high levels of autophagic flux. Importantly, however, the difference between control hNPCs + Bafilomycin A1 and DCHS1 and FAT4 hNPCs with or without Bafilomycin A1 is not significant, precluding us from suggesting the engagement of other pathways/mechanisms in regulating the migration of DCHS1 and FAT4 hNPCs. We have discussed this in the manuscript.

- Autophagosome formation seems to be intact and even occupy a larger area in DCHS1/FAT4 hNPCs, suggesting a defective fusion with lysosomes. Lysosome area and size seems to be unaltered in these organoids (fig 3B/C), even in DCHS1 hNPCs treated with metformin, which stimulates fusion with lysomes and which reduces the autophagosome area in these hNPCs. Would one not expect to see any difference in lysosome area?

This is a very good point. Indeed, we did not detect any significant differences in the area or size of lysosomes following an EM analysis of hNPCs with either DCHS1 or FAT4 mutation. However, there was always a trend, albeit not significant, toward higher values for the size of lysosomes in hNPCs with DCHS1 and FAT4 mutations, as shown in Figures 3 & 4. It should be noted that this analysis was performed using ultrathin sections of hNPC cell soma that contained 2-3 times fewer lysosomes than autophagosomes. It would be interesting, in future studies, to perform this type of analysis on fully reconstructed 3D EM images of hNPCs to assess lysosomal area and size in the entire cell and leading process of hNPCs. To address this drawback with EM analysis and the reviewer's comment, we performed other experiments by immunolabeling grafted hNPCs for LAMP1. We calculated the overall intensity of the LAMP+ signal in multiple optical sections to capture the entire cell soma and leading process. Our results revealed that there is an increase in the intensity of the immunofluorescent signal for LAMP1 in hNPCs with DCHS1 and FAT4 mutations. These new data are shown in Figure 3 and are discussed in the manuscript.

There are various typographical errors in the text and figure legends that need correction. Nomenclature of gene/protein names is inconsistent, especially in the Discussion section, and needs to be checked throughout.

We apologize for the typos and inconsistencies. The manuscript has now been thoroughly revised.

Reviewer #2:

The work of Bressan et al. investigates the mechanisms underlying the migration defects in patients with cortical malformation due to mutations in DCHS and FAT4. Using patient-derived organoids and grafting human neural progenitor cells (hNPCs) into the mouse brain, the authors focus on the in vivo context linking affected migration to autophagy defects. While the overall concept is interesting and well thought-through, the performed experiments are less convincing and lack clarity. For publication, the authors need to address to following points:

We thank the reviewer for his/her comments and suggestions. Based on the reviewer's comments we performed additional experiments and analyses to strengthen our manuscript.

Major points:

1. A major issue of this work are the biological replicates. While the authors always state how many cells have been analyzed, I did not find information on how many animals have been imaged. For example, for migration analysis, 21 control cells have been analyzed (Figure 1D). Do these 21 cells all come from one xenotransplanted animal? Then the biological replicates are too low with n=1. If 21 cells have been analyzed in different animals, the number of cells per animals is too low. And why is the cell number so different between experimental conditions (n=21 ctrl; n=47 for DCHS; n=17 FAT4)? The same applies for the time-lapse imaging of autophagy (Figure 3D, F).

We apologize for not reporting the numbers of animals in our original manuscript. This has now been added to the manuscript. We performed time-lapse imaging from multiple animals per condition. We would like to note that we grafted cells into the SVZ and imaged them in the migratory stream many mm away from the injection site. Not all hNPCs integrate into the SVZ niche and the cells that do and initiate their migration are dispersed along the entire migratory stream. While there are normally between 5-10 cells per field of view (see corresponding panels in Figures for time-lapse imaging), not all cells migrate during the 1 h imaging time (some stationary phases may be more than 1 h). As we analyzed only migratory cells, this decreased the number of analyzed cells per animal. We think that analyzing all cells across many animals (as compared to imaging multiple cells from a single animal) provides a better representation of the

dynamics of cell migration and alleviates animal-to-animal variability in the integration of xenotranplanted cells *in vivo*.

With regard to the cell numbers reported in Figures 1 & 3, we generally examined 20-30 cells per condition (Figure 1: n=21 and 17 cells for the control and FAT4 hNPCs, respectively; Figure 3: n=17, 31 and 17 cells for control cells and hNPCs with the DCHS1 or FAT4 mutation, respectively). In Figure 1 we show 47 DCHS1 hNPC cells. This higher number of imaged cells for DCHS1 hNPCs did not affect our conclusions and values. The values for DCHS1 hNPCs in Figure 1 are very similar to those in Figure 3 under baseline conditions (distance of migration 20.4 ± 1.4 µm and 19 ± 1.5 µm for Fig. 1 & Fig. 3, respectively; speed of migration 76.9 ± 4.5 µm/h and 76.8 ± 6.1 µm/h for Fig. 1 & Fig. 3, respectively; and percentage of migratory phases 28.7 ± 1.3% and 26.5 ± 1.5% for Fig. 1 & Fig. 3, respectively).

2. Information on the control line is lacking. Here, the gold standard is isogene control via CRISPR correction. Does this apply to this control line?

We have added this information to the Methods section and have imaged more control lines. These new data are shown in **Figure 1** of the revised manuscript. In total, we imaged three different control lines, one of which is an isogenic control for the DCHS1 and FAT4 knock-down lines. The information for all the control lines has been added to the Materials and Methods sections.

3. The authors write that they performed single cell RNA sequencing (scRNA seq) of control, DCHS and FAT4 organoids, but I did not find any information on that in their material and methods. My guess is that they reanalyzed an existing data set (Klaus et al., 2019), which is totally fine, but should be clearly stated.

Yes, we analyzed the scRNAseq dataset reported by Klaus et al., 2019. This is now clearly stated in the manuscript.

4. Figure 2a: Is the LC3-staining reliable? In the images shown, LC3 looks rather cytoplasmic. Here, high resolution images showing autophagosomal structures would be appreciated. Furthermore, why do the authors only show LC3-staining in control organoids? Given their

results on the western blot, it would be particularly interesting to see this staining in DCHS and FAT4-derived organoids, and to also directly combine it with Paxillin immune staining. In addition, co-stainings and western blot analyses for lysosomal markers would also be interesting to further assess autophago-lysosomal degradation in control and mutant organoids as the authors could not detect differences in the autophago-lysosomal pathway on transcriptional level.

We thank the reviewer for suggesting these experiments. We have included examples of DCHS1- and FAT4-derived organoid immunolabeling in the **Figure EV1**. With regard to the quantification of immunolabeling in organoids, we would like to point out that as most of our experiments were performed *in vivo*, we think that immunolabeling and quantifications for LC3, LAMP1, and paxillin in the fixed brain tissues derived from mice injected with control, DCHS1. and FAT4 hNPCs would be more representative. We thus performed these experiments and have also stained for Atg12 as a marker for the autophagosome initiation process. Higher resolution images and quantifications are now shown in Figures 2 & 3 and show an accumulation of paxillin and LC3, as well as LAMP1, at the level of individual DCHS1 and FAT4 hNPCs grafted into the brains of immunodeficient mice.

5. The authors report an increase in Paxillin and the lipidated form of LC3 in Western blot from DCHS and FAT4 mutant organoids. This increase is rather small than very large as stated in the text, but it is significant. An increase in LC3 can mean either more autophagy or impaired autophagy. Here, p62 expression on the same western blot would directly tell which one is the case as p62 would accumulate in case of autophagic defects. Furthermore, classical autophagy turnover assays using BafilomycinA1/Chloroquin are missing.

We observed a 2-fold increase in paxillin and a 2- to 3-fold increase in lipidated LC3 in FAT4- and DCHS1-derived organoids, respectively (Fig. 2B-C). As these are very large changes, we are not sure why the reviewer considers them as small changes. We also performed Western blotting for p62 as the reviewer requested and did not observe any differences. These new data (**Figure 2**) and a discussion of these results are now included in the revised version of the manuscript. We would like to emphasize that while both p62 and paxillin are direct targets of LC3, previous work has shown that paxillin turnover by autophagy is independent of p62 (Sharifi et al., 2016, Kenific et al., 2016). This indicates that autophagy can modulate cellular dynamics through both p62-

independent and p62-dependent mechanisms (Sharifi et al., 2016, Kenific et al., 2016). Our previous work on migrating neuroblasts in the postnatal mouse brain has also shown that the recycling of paxillin by autophagy is p62-independent (Bressan et al., 2020). Since paxillin is a direct target of LC3-II, we think that the changes in the paxillin signal in the Western blots provide the information requested by the reviewer as to whether the increase in LC3 reflects autophagy or impaired autophagy.

We also appreciate the reviewer's suggestion for the BafilomycinA1/Chloroquine assays. As our work focused on *in vivo/ex vivo* tracking of cell migration, we performed Bafilomycin A1 experiments in live brain slices and tracked the cell migration of hNPCs derived from unaffected subjects and from patients with a DCHS1 or FAT4 mutation. These experiments are shown in Figure 3. Based on the reviewer's comments, we also performed additional experiments to assess the expression levels of Atg12, LC3, LAMP, and paxillin *in vivo* in fixed brain tissues. All these experiments, together with our EM analysis of autophagosomes and lysosomes, indicate that hNPCs with a DCHS1 or FAT4 mutation exhibit an impaired autophagy process.

6. The EM pictures are nice, but it is impossible to judge how stabile the phenotype is. It is again necessary to report the biological replicates (as mentioned before). Maybe more images from different biological replicates in the Supplemental Material would help, too.

The phenotype reported from the EM images was assessed by a detailed analysis of approximately 30 cells from 3-4 animals. We now report the number of mice and also show more EM images in **Figure EV2** and **Figure EV3**, as requested by the reviewer.

7. The BafilomycinA1 concentration used in the migration studies appears very high. Since Bafilomycin is toxic to the cells, I am concerned about cell death effects. Have the authors performed a dose response curve?

We have performed a dose-response curve for our previous work (Bressan et al., 2020) and tested three different concentrations. These data are shown below.

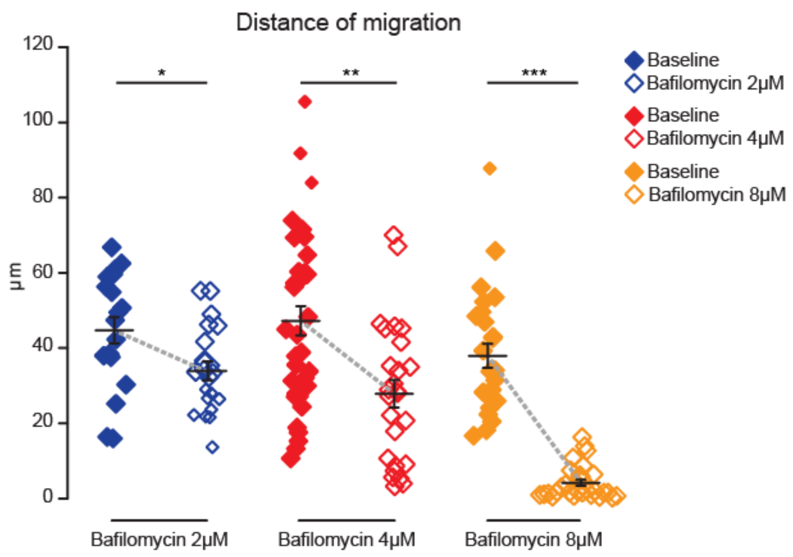

8. The in vivo rescue with Metformin is very interesting. However, also here, overall biochemical assays, such as Western blot analyses for LC3 and p62, lysosomal markers, autophagy turnover assays using BafilomycinA1/Chloroquin as well as immunostainings against LC3 and Paxillin would be needed to first verify that Metformin is actual rescuing the suggested autophagic defect by decreasing Paxillin levels. This would significantly strengthen their hypothesis that the accumulation of Paxillin due to impairments in autophagy dependent degradation might lead to the observed migration deficit.

Unfortunately, these experiments are not feasible given that hNPCs represent a minority of cells in the mouse brain. As such, the Western blots and the autophagy turnover assay will not yield results representative of hNPCs but will rather reflect the autophagy dynamic of mouse neuroblasts. FACS sorting hNPCs from mouse brains would be extremely challenging and would require pooling many animals to increase the hNPC-specific protein yield required for Western blots and the autophagy turnover assay. We thank the reviewer for suggesting immunostaining for LC3 and paxillin. We think that our EM analysis provides the information requested by the reviewer at a better resolution and shows the rescue effect on autophagy.

Reviewer #3:

Dear editor find attached my comments on manuscript EMM-2022-16908, entitled " Periventricular heterotopia induced by DCHS1 and FAT4 mutations is linked to autophagy-

dependent alterations in neuronal migration and can be rescued by metformin" by Bressan et al.

Overal I found the paper very interesting, highly relevant, and technically very challenging, which the authors should be commended on their hard work. , and I think its worthy of publication in your journal. However, there are a few issues I would like the authors to address, before this can happen.

We thank the reviewer for his/her positive comments and suggestions regarding our manuscript. We have strengthened our manuscript by performing new experiments/analyses.

Figure 1, Legend section D , sentence starting " n=40 cells
for endogenous neuroblasts, n=21 cells for control hNPCs, n=47 cells for hNPCs with a mutation in DCHS1, n=17 cells for hNPCs with a mutation in.* $p < 0.05$, ** $p < 0.005$, *** $p < 0.001$ with a one-way ANOVA followed by a post hoc LSD Fisher." FAT4 is missing!

Thank you for noticing this. We have corrected the text and have added FAT4.

Figure 2 A-not sure if immunos are really showing or telling us very much here, as these are only performed on control organoids. Also they need to clarify what DCX is staining for, whether its migrating interneurons, or other types of cells?, as this is not clearly stated in text.

We now show immunolabeling for organoids derived from FAT4 and DCHS1 hNPCs in **Figure EV1** and have clarified the purpose of DCX staining. We have also performed additional *in vivo* immunostaining and quantification experiments in xenotransplanted hNPCs to strengthen our conclusions (please see our response below and the response to point #4 of reviewer #2).

Figure 2B The author stress the increase in levels of LC3-II in the western blots, but there is also clearly an increase in LC3-I , which is not nemtioned? Also how do the levels compare against each other, so if authors could calulate the ratios between the 2, it would be interesting to see if this changes or stays the same!

LC3-I levels are much higher in neural tissue compared to LC3-II (Klionsky et al., 2012). In addition, LC3-I levels are less representative of changes in autophagy. In our experiments, LC3-I

signals were overexposed to more accurately reveal LC3-II levels (**Figure 2**). This makes the quantification of LC3-I levels and the ratio between LC3-II and LC3-I inaccurate. Because of this and other caveats associated with the LC3-I analysis, it has been also suggested in the guidelines for assessment of autophagy (Klionsky et al., 2008; 2012; 2021) to normalize LC3-II levels to houskeeping genes rather than LC3-I levels. We thus used this approach and normalized LC3-II levels to housekeeping gene levels.

It would also be great if the authors could also confirm their western blot findings by either show immunohstochemisrty or QPCR for LC3-I and LC3-II , and paxillin in control.FAT4 and DCHs1 cultures.

We confirmed our Western blot data by immunolabeling for LC3, Atg12, and paxillin *in vivo* in xenotransplanted hNPCs.

Figure 3. To be honest I found it very difficult to see what is going on in the EM pictures, and to see any obvious differences between the images shown. The only comment I can make is that there are no lysosomes highlighted inDCHs1 or FAT4 cultures, is this true, also same comment for 4D? So it would be better for the authors to remove white arrows pointing to gfp gold labelling and add more arrow heads pointing out autophagosomes and lysosomes, so the reader can make an informed decision about what is being shown. This also applies to EM in figure 4

Indeed, some EM images of hNPCs contained autophagosomes but not lysosomes, which are present at a smaller density as shown by our quantification. We have now revised these figures to *i)* show that there are more autophagosomes in hNPCs with DCHS1 and FAT4 mutations than in the control hNPCs and *ii)* demonstrate both autophagosomes and lysosomes, indicated by white and red arrowheads, respectively. We have also kept only one white arrow pointing to gold particles on only one image. Finally, we have included additional EM images in **Figure EV2** and **Figure EV3**.

7th Jul 2023

Dear Dr. Saghatelyan,

Thank you for the submission of your revised manuscript to EMBO Molecular Medicine. I am pleased to inform you that we will be able to accept your manuscript pending the following final amendments:

1) Please address the referee's #2 minor points by discussion.
2) Please reformat your manuscript to "Research article". All EV figures should be made main figures, with their callouts updated in the main manuscript text. Please check "Author Guidelines" for more information:
https://www.embopress.org/page/journal/17574684/authorguide#researcharticleguide
3) In the main manuscript file, please do the following:
- Correct/answer the track changes suggested by our data editors by working from the attached document.
- Add up to 5 keywords.
- Remove font color.
- Add callouts for individual panels in Fig. EV1.
- Remove all figures and move figure legends to the end of the manuscript after "References".
- Remove movie legends. A movie legend should be in a READ ME file and zipped together with its corresponding movie.
- Please rename "Competing Interest" to "Disclosure Statement & Competing Interests". We updated our journal's competing interests policy in January 2022 and request authors to consider both actual and perceived competing interests. Please review the policy https://www.embopress.org/competing-interests and update your competing interests if necessary.
- Author contributions: Please specify author contributions in our submission system. CRediT has replaced the traditional author contributions section because it offers a systematic machine-readable author contributions format that allows for more effective research assessment. You are encouraged to use the free text boxes beneath each contributing author's name to add specific details on the author's contribution. More information is available in our guide to authors:
https://www.embopress.org/page/journal/17574684/authorguide#authorshipguidelines
- Please place following sections after "Materials and Methods" in the subsequent order: data availability section, acknowledgments, disclosure statement and competing interests, references, main figure legends, (expanded figure legends).
4) Funding: Please make sure that information about all sources of funding are complete in both our submission system and in the manuscript. Currently ERA-Net Neuron (nEUrotalk | 01EW1907) is missing in our submission system.
5) Synopsis:
- Synopsis text: Please remove it from the main manuscript file and upload it as a separate .doc file.
- Synopsis image: Please provide a striking image or visual abstract as a high-resolution jpeg file 550 px-wide x (250-400)-px high to illustrate your article.
- Please check your synopsis text and image before submission with your revised manuscript. Please be aware that in the proof stage minor corrections only are allowed (e.g., typos).
6) For more information: This space should be used to list relevant web links for further consultation by our readers. Could you identify some relevant ones and provide such information as well? Some examples are patient associations, relevant databases, OMIM/proteins/genes links, author's websites, etc...
7) Source data: Please upload source data as clearly labelled one (zipped) file per figure (please also check panel labels).
8) As part of the EMBO Publications transparent editorial process initiative (see our Editorial at http://embomolmed.embopress.org/content/2/9/329), EMBO Molecular Medicine will publish online a Review Process File (RPF) to accompany accepted manuscripts. This file will be published in conjunction with your paper and will include the anonymous referee reports, your point-by-point response and all pertinent correspondence relating to the manuscript. Let us know whether you agree with the publication of the RPF and as here, if you want to remove or not any figures from it prior to publication. Please note that the Authors checklist will be published at the end of the RPF.
9) Please provide a point-by-point letter INCLUDING my comments as well as the reviewer's reports and your detailed responses (as Word file).

I look forward to reading a new revised version of your manuscript as soon as possible.

Yours sincerely,

Zeljko Durdevic

Zeljko Durdevic
Editor
EMBO Molecular Medicine

*** Instructions to submit your revised manuscript ***

*** PLEASE NOTE *** As part of the EMBO Publications transparent editorial process initiative (see our Editorial at https://www.embopress.org/doi/pdf/10.1002/emmm.201000094), EMBO Molecular Medicine will publish online a Review Process File to accompany accepted manuscripts.

In the event of acceptance, this file will be published in conjunction with your paper and will include the anonymous referee reports, your point-by-point response and all pertinent correspondence relating to the manuscript. If you do NOT want this file to be published, please inform the editorial office at contact@embomolmed.org.

When submitting your revised manuscript, please include:

1) a .docx formatted version of the manuscript text (including Figure legends and tables)

2) Separate figure files*

3) supplemental information as Expanded View and/or Appendix. Please carefully check the authors guidelines for formatting Expanded view and Appendix figures and tables at https://www.embopress.org/page/journal/17574684/authorguide#expandedview

4) a letter INCLUDING the reviewer's reports and your detailed responses to their comments (as Word file).

5) The paper explained: EMBO Molecular Medicine articles are accompanied by a summary of the articles to emphasize the major findings in the paper and their medical implications for the non-specialist reader. Please provide a draft summary of your article highlighting
- the medical issue you are addressing,
- the results obtained and
- their clinical impact.
This may be edited to ensure that readers understand the significance and context of the research.
Please refer to any of our published articles for an example.

6) For more information: There is space at the end of each article to list relevant web links for further consultation by our readers. Could you identify some relevant ones and provide such information as well? Some examples are patient associations, relevant databases, OMIM/proteins/genes links, author's websites, etc...

7) Author contributions: the contribution of every author must be detailed in a separate section.

8) EMBO Molecular Medicine now requires a complete author checklist (https://www.embopress.org/page/journal/17574684/authorguide) to be submitted with all revised manuscripts. Please use the checklist as guideline for the sort of information we need WITHIN the manuscript. The checklist should only be filled with page numbers were the information can be found. This is particularly important for animal reporting, antibody dilutions (missing) and exact values and n that should be indicted instead of a range.

9) Every published paper now includes a 'Synopsis' to further enhance discoverability. Synopses are displayed on the journal webpage and are freely accessible to all readers. They include a short stand first (maximum of 300 characters, including space) as well as 2-5 one sentence bullet points that summarise the paper. Please write the bullet points to summarise the key NEW findings. They should be designed to be complementary to the abstract - i.e. not repeat the same text. We encourage inclusion of key acronyms and quantitative information (maximum of 30 words / bullet point). Please use the passive voice. Please attach these in a separate file or send them by email, we will incorporate them accordingly.

You are also welcome to suggest a striking image or visual abstract to illustrate your article. If you do please provide a jpeg file 550 px-wide x 400-px high.

10) A Conflict of Interest statement should be provided in the main text

11) Please note that we now mandate that all corresponding authors list an ORCID digital identifier. This takes <90 seconds to

complete. We encourage all authors to supply an ORCID identifier, which will be linked to their name for unambiguous name identification.

Currently, our records indicate that the ORCID for your account is 0000-0003-4962-0465.

Please click the link below to modify this ORCID:
Link Not Available

12) The system will prompt you to fill in your funding and payment information. This will allow Wiley to send you a quote for the article processing charge (APC) in case of acceptance. This quote takes into account any reduction or fee waivers that you may be eligible for. Authors do not need to pay any fees before their manuscript is accepted and transferred to our publisher.

*Additional important information regarding Figures

Each figure should be given in a separate file and should have the following resolution:
Graphs 800-1,200 DPI
Photos 400-800 DPI
Colour (only CMYK) 300-400 DPI"

Figures are not edited by the production team. All lettering should be the same size and style; figure panels should be indicated by capital letters (A, B, C etc). Gridlines are not allowed except for log plots. Figures should be numbered in the order of their appearance in the text with Arabic numerals. Each Figure must have a separate legend and a caption is needed for each panel.

*Additional important information regarding figures and illustrations can be found at
https://bit.ly/EMBOPressFigurePreparationGuideline. See also figure legend preparation guidelines:
https://www.embopress.org/page/journal/17574684/authorguide#figureformat

The system will prompt you to fill in your funding and payment information. This will allow Wiley to send you a quote for the article processing charge (APC) in case of acceptance. This quote takes into account any reduction or fee waivers that you may be eligible for. Authors do not need to pay any fees before their manuscript is accepted and transferred to our publisher.

***** Reviewer's comments *****

Referee #2 (Remarks for Author):

In the revised version of their manuscript, the authors have addressed most of my points raised and I appreciated the inclusion of additional control lines.
My questions and comment concerning the autophagy part have been only partially answered: (1) The quantification of LC3, LAMP1 and paxillin in vivo is fine, however, the images shown of LC3 immunostaining are not very convincing as I cannot really identify autophagosomes. The LC3 signal looks rather cytoplasmic. Furthermore, separate stainings of LC3 and Paxillin are not helping to see their co-localization. Instead, my original request was on co-stainings with LC3 and Paxillin. (2) The authors did not perform the suggested autophagic flux turnover assays via Western blot. The in vivo Bafilomycin injections do not address the autophagic flux either, but cell migration. Hence, autophagy flux has not been investigated. Since the authors did not make a major point on autophagic flux and also changed the title according and in light of the additional experiments that have been performed as well as the general improvement of the manuscript, I suggest accepting the work for publication in EMBO Journal.

Referee #3 (Comments on Novelty/Model System for Author):

This is a technological difficult study, which has now been massively improved from the original submission

Referee #3 (Remarks for Author):

I have read through the revised manuscript, and response to all the reviewers comments. Most of the issues raised by all , appear to have been carried out. The manuscript is much improved over the initial submission, by all the extra experiments carried out.
Im happy to recommend acceptance of this mansucript for publication

Reviewer #2:

In the revised version of their manuscript, the authors have addressed most of my points raised and I appreciated the inclusion of additional control lines.
My questions and comment concerning the autophagy part have been only partially answered: (1) The quantification of LC3, LAMP1 and paxillin in vivo is fine, however, the images shown of LC3 immunostaining are not very convincing as I cannot really identify autophagosomes. The LC3 signal looks rather cytoplasmic. Furthermore, separate stainings of LC3 and Paxillin are not helping to see their co-localization. Instead, my original request was on co-stainings with LC3 and Paxillin. (2) The authors did not perform the suggested autophagic flux turnover assays via Western blot. The in vivo Bafilomycin injections do not address the autophagic flux either, but cell migration. Hence, autophagy flux has not been investigated. Since the authors did not make a major point on autophagic flux and also changed the title according and in light of the additional experiments that have been performed as well as the general improvement of the manuscript, I suggest accepting the work for publication in EMBO Journal.

We thank the reviewer for his/her comments. To address the reviewer's comments, we added arrowheads pointing to punctuate labeling of LC3 (**Figure 2D**) and discussed paxillin and LC3 co-labeling in the discussion.

Reviewer #3:
(Comments on Novelty/Model System for Author):

This is a technological difficult study, which has now been massively improved from the original submission

(Remarks for Author):

I have read through the revised manuscript, and response to all the reviewers comments. Most of the issues raised by all , appear to have been carried out. The manuscript is much improved over the initial submission, by all the extra experiments carried out.
Im happy to recommend acceptance of this mansucript for publication

We thank the reviewer for his/her positive comments and suggestions regarding our manuscript.

10th Aug 2023

Dear Dr. Saghatelyan,

We are pleased to inform you that your manuscript is accepted for publication and is now being sent to our publisher to be included in the next available issue of EMBO Molecular Medicine.

Please read below for additional IMPORTANT information regarding your article, its publication and the production process.

Congratulations on your interesting work,

Zeljko Durdevic

Zeljko Durdevic
Editor
EMBO Molecular Medicine

Follow us on Twitter @EmboMolMed
Sign up for eTOCs at embopress.org/alertsfeeds

*** *** *** IMPORTANT INFORMATION *** *** ***

SPEED OF PUBLICATION
The journal aims for rapid publication of papers, using using the advance online publication "Early View" to expedite the process: A properly copy-edited and formatted version will be published as "Early View" after the proofs have been corrected. Please help the Editors and publisher avoid delays by providing e-mail address(es), telephone and fax numbers at which author(s) can be contacted.

Should you be planning a Press Release on your article, please get in contact with embomolmed@wiley.com as early as possible, in order to coordinate publication and release dates.

LICENSE AND PAYMENT:

All articles published in EMBO Molecular Medicine are fully open access: immediately and freely available to read, download and share.

EMBO Molecular Medicine charges an article processing charge (APC) to cover the publication costs. You, as the corresponding author for this manuscript, should have already received a quote with the article processing fee separately. Please let us know in case this quote has not been received.

Once your article is at Wiley for editorial production you will receive an email from Wiley's Author Services system, which will ask you to log in and will present you with the publication license form for completion. Within the same system the publication fee can be paid by credit card, an invoice, pro forma invoice or purchase order can be requested.

Payment of the publication charge and the signed Open Access Agreement form must be received before the article can be published online.

PROOFS

You will receive the proofs by e-mail approximately 2 weeks after all relevant files have been sent o our Production Office. Please return them within 48 hours and if there should be any problems, please contact the production office at embopressproduction@wiley.com.

Please inform us if there is likely to be any difficulty in reaching you at the above address at that time. Failure to meet our

deadlines may result in a delay of publication.

All further communications concerning your paper proofs should quote reference number EMM-2022-16908-V3 and be directed to the production office at embopressproduction@wiley.com.

Thank you,

Zeljko Durdevic
Editor
EMBO Molecular Medicine

EMBO Press Author Checklist

Corresponding Author Name: Armen Saghatelyan	
Journal Submitted to: EMBO Molecular Medicine	
Manuscript Number: EMM-2022-16908	

USEFUL LINKS FOR COMPLETING THIS FORM
The EMBO Journal - Author Guidelines
EMBO Reports - Author Guidelines
Molecular Systems Biology - Author Guidelines
EMBO Molecular Medicine - Author Guidelines

Reporting Checklist for Life Science Articles (updated January
This checklist is adapted from Materials Design Analysis Reporting (MDAR) Checklist for Authors. MDAR establishes a minimum set of requirements in transparent reporting in the life sciences (see Statement of Task: 10.31222/osf.io/9sm4x). Please follow the journal's guidelines in preparing your
Please note that a copy of this checklist will be published alongside your article.

Abridged guidelines for figures
1. Data
The data shown in figures should satisfy the following conditions:

- the data were obtained and processed according to the field's best practice and are presented to reflect the results of the experiments in an accurate and unbiased manner.
- ideally, figure panels should include only measurements that are directly comparable to each other and obtained with the same assay.
- plots include clearly labeled error bars for independent experiments and sample sizes. Unless justified, error bars should not be shown for technical
- if n<5, the individual data points from each experiment should be plotted. Any statistical test employed should be justified.
- Source Data should be included to report the data underlying figures according to the guidelines set out in the authorship guidelines on Data

2. Captions
Each figure caption should contain the following information, for each panel where they are relevant:

- a specification of the experimental system investigated (eg cell line, species name).
- the assay(s) and method(s) used to carry out the reported observations and measurements.
- an explicit mention of the biological and chemical entity(ies) that are being measured.
- an explicit mention of the biological and chemical entity(ies) that are altered/varied/perturbed in a controlled manner.
- the exact sample size (n) for each experimental group/condition, given as a number, not a range;
- a description of the sample collection allowing the reader to understand whether the samples represent technical or biological replicates (including how many animals, litters, cultures, etc.).
- a statement of how many times the experiment shown was independently replicated in the laboratory.
- definitions of statistical methods and measures:
  - common tests, such as t-test (please specify whether paired vs. unpaired), simple χ2 tests, Wilcoxon and Mann-Whitney tests, can be unambiguously identified by name only, but more complex techniques should be described in the methods section;
  - are tests one-sided or two-sided?
  - are there adjustments for multiple comparisons?
  - exact statistical test results, e.g., P values = x but not P values < x;
  - definition of 'center values' as median or average;
  - definition of error bars as s.d. or s.e.m.

Please complete ALL of the questions below.
Select "Not Applicable" only when the requested information is not relevant for your study.

Materials

Newly Created Materials	Information included in the manuscript?	In which section is the information available? (Reagents and Tools Table, Materials and Methods, Figures, Data Availability Section)
New materials and reagents need to be available; do any restrictions apply?	Not Applicable	

Antibodies	Information included in the manuscript?	In which section is the information available? (Reagents and Tools Table, Materials and Methods, Figures, Data Availability Section)
For **antibodies** provide the following information:<br>- Commercial antibodies: RRID (if possible) or supplier name, catalogue number and or/clone number<br>- Non-commercial: RRID or citation	Yes	Materials and Methods

DNA and RNA sequences	Information included in the manuscript?	In which section is the information available? (Reagents and Tools Table, Materials and Methods, Figures, Data Availability Section)
**Short novel DNA or RNA including primers, probes**: provide the sequences.	Not Applicable	

Cell materials	Information included in the manuscript?	In which section is the information available? (Reagents and Tools Table, Materials and Methods, Figures, Data Availability Section)
**Cell lines:** Provide species information, strain. Provide accession number in repository **OR** supplier name, catalog number, clone number, and/**OR** RRID.	Yes	Materials and Methods. We referenced Klaus et al., 2019; Zaslavsky et al., 2019; and Deneault et al., 2019 that established and characterized iPSCs used in our study
**Primary cultures:** Provide species, strain, sex of origin, genetic modification status.	Not Applicable	
Report if the cell lines were recently **authenticated** (e.g., by STR profiling) and tested for mycoplasma contamination.	Not Applicable	

Experimental animals	Information included in the manuscript?	In which section is the information available? (Reagents and Tools Table, Materials and Methods, Figures, Data Availability Section)
**Laboratory animals or Model organisms:** Provide species, strain, sex, age, genetic modification status. Provide accession number in repository **OR** supplier name, catalog number, clone number, **OR** RRID.	Yes	Materials and Methods
**Animal observed in or captured from the field:** Provide species, sex, and age where possible**.**	Not Applicable	
Please detail **housing and husbandry conditions**.	Yes	Materials and Methods

Plants and microbes	Information included in the manuscript?	In which section is the information available? (Reagents and Tools Table, Materials and Methods, Figures, Data Availability Section)
**Plants:** provide species and strain, ecotype and cultivar where relevant, unique accession number if available, and source (including location for collected wild specimens).	Not Applicable	
**Microbes:** provide species and strain, unique accession number if available, and source.	Not Applicable	

Human research participants	Information included in the manuscript?	In which section is the information available? (Reagents and Tools Table, Materials and Methods, Figures, Data Availability Section)
If collected and within the bounds of privacy constraints report on age, sex and gender or ethnicity for all study participants.	Not Applicable	

Core facilities	Information included in the manuscript?	In which section is the information available? (Reagents and Tools Table, Materials and Methods, Figures, Data Availability Section)
If your work benefited from core facilities, was their service mentioned in the acknowledgments section?	Not Applicable	

Design

Study protocol	Information included in the manuscript?	In which section is the information available? (Reagents and Tools Table, Materials and Methods, Figures, Data Availability Section)
If study protocol has been **pre-registered, provide DOI in the manuscript**. For clinical trials, provide the trial registration number **OR** cite DOI.	Not Applicable	
Report the **clinical trial registration number** (at ClinicalTrials.gov or equivalent), where applicable.	Not Applicable	

Laboratory protocol	Information included in the manuscript?	In which section is the information available? (Reagents and Tools Table, Materials and Methods, Figures, Data Availability Section)
Provide DOI OR other citation details if **external detailed step-by-step protocols** are available.	Yes	Material and Methods. Bakhshetyan, K., and Saghatelyan, A. 2015 for time-lapse imaging of cell migration

Experimental study design and statistics	Information included in the manuscript?	In which section is the information available? (Reagents and Tools Table, Materials and Methods, Figures, Data Availability Section)
Include a statement about **sample size** estimate even if no statistical methods were used.	Yes	Material and Methods
Were any steps taken to minimize the effects of subjective bias when allocating animals/samples to treatment (e.g. **randomization procedure**)? If yes, have they been described?	Not Applicable	
Include a statement about **blinding** even if no blinding was done.	Yes	Material and Methods
Describe **inclusion/exclusion criteria** if samples or animals were excluded from the analysis. Were the criteria pre-established?<br><br>If sample or data points were omitted from analysis, report if this was due to attrition or intentional exclusion and provide justification.	Yes	Material and Methods
For every figure, are **statistical tests** justified as appropriate? Do the data meet the assumptions of the tests (e.g., normal distribution)? Describe any methods used to assess it. Is there an estimate of variation within each group of data? Is the variance similar between the groups that are being statistically compared?	Yes	Figure Legends for statistical tests and Material and Methods

Sample definition and in-laboratory replication	Information included in the manuscript?	In which section is the information available? (Reagents and Tools Table, Materials and Methods, Figures, Data Availability Section)
In the figure legends: state number of times the experiment was **replicated** in laboratory.	Yes	corresponding Figure Legends
In the figure legends: define whether data describe **technical or biological replicates**.	Yes	we report both cell and animal numbers in theFigure legends

Ethics

Ethics	Information included in the manuscript?	In which section is the information available? (Reagents and Tools Table, Materials and Methods, Figures, Data Availability Section)
Studies involving **human participants**: State details of **authority granting ethics approval** (IRB or equivalent committee(s), provide reference number for approval.	Yes	Materail and Methods
Studies involving **human participants**: Include a statement confirming that **informed consent** was obtained from all subjects and that the experiments conformed to the principles set out in the WMA Declaration of Helsinki and the Department of Health and Human Services Belmont Report.	Yes	Materials and Methods. We referenced Klaus et al., 2019; Zaslavsky et al., 2019; and Deneault et al., 2019 that established and characterized iPSCs used in our study
Studies involving **human participants:** For publication of **patient photos**, include a statement confirming that consent to publish was obtained.	Not Applicable	
Studies involving experimental **animals**: State details of **authority granting ethics approval** (IRB or equivalent committee(s), provide reference number for approval. Include a statement of compliance with ethical regulations.	Yes	Materail and Methods
Studies involving **specimen and field samples:** State if relevant **permits** obtained, provide details of authority approving study; if none were required, explain why.	Not Applicable	

Dual Use Research of Concern (DURC)	Information included in the manuscript?	In which section is the information available? (Reagents and Tools Table, Materials and Methods, Figures, Data Availability Section)
Could your study fall under dual use research restrictions? Please check biosecurity documents and list of **select agents and toxins** (CDC): https://www.selectagents.gov/sat/list.htm	Not Applicable	
If you used a select agent, is the security level of the lab appropriate and reported in the manuscript?	Not Applicable	
If a study is subject to dual use research of concern regulations, is the name of the **authority granting approval and reference number** for the regulatory approval provided in the manuscript?	Not Applicable	

Reporting

The MDAR framework recommends adoption of discipline-specific guidelines, established and endorsed through community initiatives. Journals have their own policy about requiring specific guidelines and recommendations to complement MDAR.

Adherence to community standards	Information included in the manuscript?	In which section is the information available? (Reagents and Tools Table, Materials and Methods, Figures, Data Availability Section)
State if relevant guidelines or checklists (e.g., **ICMJE, MIBBI, ARRIVE, PRISMA**) have been followed or provided.	Not Applicable	
For **tumor marker prognostic studies,** we recommend that you follow the **REMARK** reporting guidelines (see link list at top right). See author guidelines, under 'Reporting Guidelines'. Please confirm you have followed these guidelines.	Not Applicable	
For **phase II and III randomized controlled trials**, please refer to the **CONSORT** flow diagram (see link list at top right) and submit the CONSORT checklist (see link list at top right) with your submission. See author guidelines, under 'Reporting Guidelines'. Please confirm you have submitted this list.	Not Applicable	

Data Availability

Data availability	Information included in the manuscript?	In which section is the information available? (Reagents and Tools Table, Materials and Methods, Figures, Data Availability Section)
Have **primary datasets** been deposited according to the journal's guidelines (see 'Data Deposition' section) and the respective accession numbers provided in the Data Availability Section?	Not Applicable	
Were **human clinical and genomic datasets** deposited in a public access-controlled repository in accordance to ethical obligations to the patients and to the applicable consent agreement?	Not Applicable	
Are **computational models** that are central and integral to a study available without restrictions in a machine-readable form? Were the relevant accession numbers or links provided?	Not Applicable	
If publicly available data were reused, provide the respective **data citations in the reference list**.	Yes	Klaus et al, 2019 in the Reference list
